# Widespread natural variation of DNA methylation within angiosperms

**DOI:** 10.1186/s13059-016-1059-0

**Published:** 2016-09-27

**Authors:** Chad E. Niederhuth, Adam J. Bewick, Lexiang Ji, Magdy S. Alabady, Kyung Do Kim, Qing Li, Nicholas A. Rohr, Aditi Rambani, John M. Burke, Joshua A. Udall, Chiedozie Egesi, Jeremy Schmutz, Jane Grimwood, Scott A. Jackson, Nathan M. Springer, Robert J. Schmitz

**Affiliations:** 1 Department of Genetics, University of Georgia, 120 East Green Street, Athens, GA 30602 USA; 2 Institute of Bioinformatics, University of Georgia, Athens, GA 30602 USA; 3 Department of Plant Biology, University of Georgia, Athens, GA 30602 USA; 4 Center for Applied Genetic Technologies, University of Georgia, Athens, GA 30602 USA; 5 Department of Plant Biology, Microbial and Plant Genomics Institute, University of Minnesota, Saint Paul, MN 55108 USA; 6 Plant and Wildlife Science Department, Brigham Young University, Provo, UT 84602 USA; 7 National Root Crops Research Institute (NRCRI), Umudike, Km 8 Ikot Ekpene Road, PMB 7006, Umuahia, 440001 Nigeria; 8 HudsonAlpha Institute for Biotechnology, Huntsville, AL 35806 USA; 9 Department of Energy Joint Genome Institute, Walnut Creek, CA USA

## Abstract

**Background:**

DNA methylation is an important feature of plant epigenomes, involved in the formation of heterochromatin and affecting gene expression. Extensive variation of DNA methylation patterns within a species has been uncovered from studies of natural variation. However, the extent to which DNA methylation varies between flowering plant species is still unclear. To understand the variation in genomic patterning of DNA methylation across flowering plant species, we compared single base resolution DNA methylomes of 34 diverse angiosperm species.

**Results:**

By analyzing whole-genome bisulfite sequencing data in a phylogenetic context, it becomes clear that there is extensive variation throughout angiosperms in gene body DNA methylation, euchromatic silencing of transposons and repeats, as well as silencing of heterochromatic transposons. The Brassicaceae have reduced CHG methylation levels and also reduced or loss of CG gene body methylation. The Poaceae are characterized by a lack or reduction of heterochromatic CHH methylation and enrichment of CHH methylation in genic regions. Furthermore, low levels of CHH methylation are observed in a number of species, especially in clonally propagated species.

**Conclusions:**

These results reveal the extent of variation in DNA methylation in angiosperms and show that DNA methylation patterns are broadly a reflection of the evolutionary and life histories of plant species.

**Electronic supplementary material:**

The online version of this article (doi:10.1186/s13059-016-1059-0) contains supplementary material, which is available to authorized users.

## Background

Biological diversity is established at multiple levels. Historically this has focused on studying the contribution of genetic variation. However, epigenetic variations manifested in the form of DNA methylation [1–3], histones and histone modifications [4], which together make up the epigenome, might also contribute to biological diversity. These components are integral to proper regulation of many aspects of the genome; including chromatin structure, transposon silencing, regulation of gene expression, and recombination [5–8]. Significant amounts of epigenomic diversity are explained by genetic variation [2, 3, 9–13], however, a large portion remains unexplained and in some cases these variants arise independently of genetic variation and are thus defined as “epigenetic” [2, 10–12, 14, 15]. Moreover, epigenetic variants can be heritable and also lead to phenotypic variation [16–19]. To date, most studies of epigenomic variation in plants are based on a handful of model systems. Current knowledge is, in particular, based upon studies in *Arabidopsis thaliana*, which is tolerant to significant reductions in DNA methylation, a feature that enabled the discovery of many of the underlying mechanisms. However, *A. thaliana* has a particularly compact genome, when most plant genomes are much larger [20, 21]. The extent of natural variation of mechanisms that lead to epigenomic variation in plants, such as cytosine DNA methylation, is unknown and understanding this diversity is important to understanding the potential of epigenetic variation to contribute to phenotypic variation [22].

In plants, cytosine methylation occurs in three sequence contexts; CG, CHG, and CHH (H = A, T, or C), and are under control by distinct mechanisms [23]. Methylation at CG (mCG) and CHG (mCHG) sites is typically symmetrical across the Watson and Crick strands [24]. mCG is maintained by methyltransferase 1 (MET1), which is recruited to hemi-methylated CG sites and methylates the opposing strand [25, 26], whereas mCHG is maintained by the plant specific chromomethylase 3 (CMT3) [27], and is strongly associated with dimethylation of lysine 9 on histone 3 (H3K9me2) [28]. The BAH and CHROMO domains of CMT3 bind to H3K9me2, leading to methylation of CHG sites [28]. In turn, the histone methyltransferases kryptonite (KYP), and Su(var)3-9 homologue 5 (SUVH5) and SUVH6 recognize methylated DNA and methylate H3K9 [29], leading to a self-reinforcing loop [30]. Asymmetrical methylation of CHH sites (mCHH) is established and maintained by another member of the CMT family, CMT2 [31, 32]. CMT2, like CMT3, also contains BAH and CHROMO domains and methylates CHH in H3K9me2 regions [31, 32]. Additionally, all three sequence contexts are methylated de novo via RNA-directed DNA methylation (RdDM) [33]. Short-interfering 24 nucleotide (nt) RNAs (siRNAs) guide the de novo methyltransferase domains rearranged methyltransferase 2 (DRM2) to target sites [34, 35]. The targets of CMT2 and RdDM are often complementary, as CMT2 in *A. thaliana* primarily methylate regions of deep heterochromatin, such as transposons bodies [31]. RdDM regions, on the other hand, often have the highest levels of mCHH methylation and primarily target the edges of transposons and the more recently identified mCHH islands [31, 32, 36] The mCHH islands in *Zea mays* are associated with upstream and downstream of more highly expressed genes where they might function to prevent transcription of neighboring transposons [36, 37]. The establishment, maintenance, and consequences of DNA methylation are therefore highly dependent upon the species and upon the particular context in which it is found.

Sequencing and array-based methods allow for studying DNA methylation across entire genomes and within species [1, 3, 13, 15, 38]. Whole-genome bisulfite sequencing (WGBS) is particularly powerful, as it reveals genome-wide single nucleotide resolution of DNA methylation [39–41]. WGBS has been used to sequence an increasing number of plant methylomes, ranging from model plants like *A. thaliana* [39, 40] to economically important crops like *Z. mays* [2, 11, 36, 42]. This has enabled a new field of comparative epigenomics, which places DNA methylation within an evolutionary context [43–46]. The use of WGBS together with de novo transcript assemblies has provided an opportunity to monitor the changes in DNA methylation of gene bodies among species [47] but does not provide a full view of changes in the patterns of context-specific DNA methylation at different types of genomic regions [48].

Here, we report a comparative epigenomics study of 34 angiosperms (flowering plants). Differences in mCG and mCHG are in part driven by repetitive DNA and genome size, whereas in the Brassicaceae there are lower mCHG levels and lower numbers or even losses of CG gene body methylation (gbM) when compared to other species. The Poaceae are distinct from other lineages, having low mCHH levels and a lineage-specific distribution of mCHH in the genome. Additionally, species that have been clonally propagated often have low levels of mCHH. Although some features, such as mCHH islands, are found in all species, their association with effects on gene expression is not universal. The extensive variation found suggests that both genomic, life history, and mechanistic differences between species contribute to this variation.

## Results

### Genome-wide DNA methylation variation across angiosperms

**Fig. 1:**
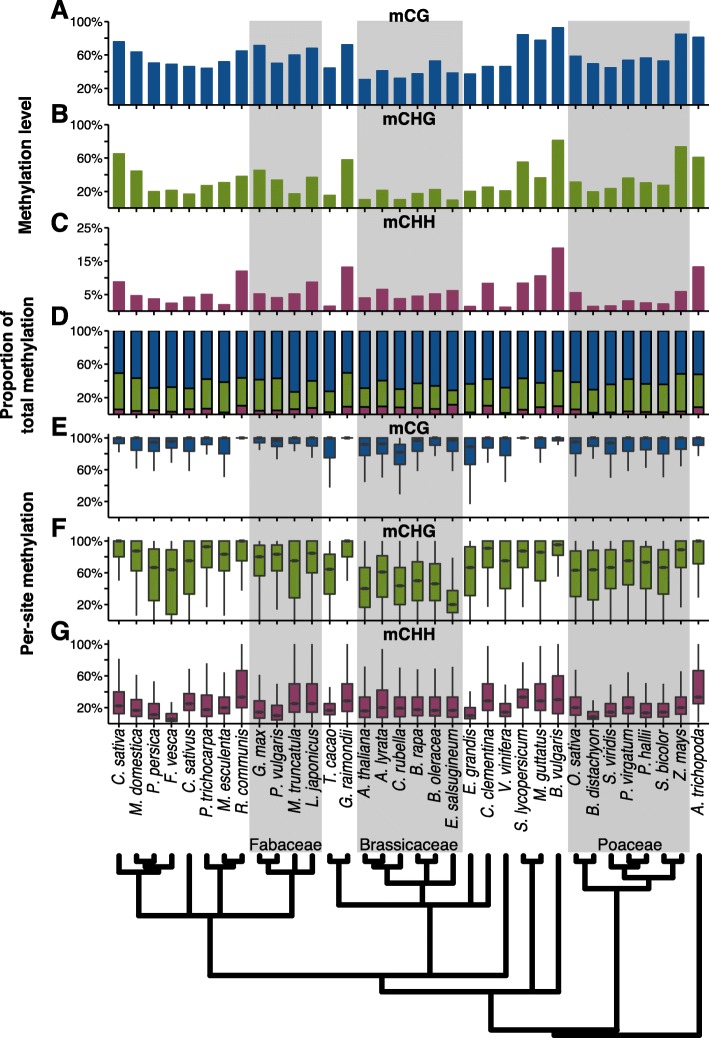
Genome-wide methylation levels for **a** mCG, **b** mCHG, and **c** mCHH. **d** Using the genome-wide methylation levels, the proportion that each context contributes towards the total methylation (mC) was calculated. **e** The distribution of per-site methylation levels for mCG, **f** mCHG, and **g** mCHH. Species are organized according to their phylogenetic relationship

We compared single-base resolution methylomes from the leaves of 34 angiosperm species that have genome assemblies [49–52] (Additional file 1: Table S1). MethylC-seq [40, 53] was used to sequence 26 species and an additional eight species with previously published methylomes were downloaded and reanalyzed [12, 15, 36, 48, 54–56]. Different metrics were used to make comparisons at a whole-genome level. The genome-wide weighted DNA methylation level [57] combines data from the number of instances of methylated cytosine sites relative to all sequenced cytosine sites, giving a single value for each context that can be compared across species (Fig. 1a–c). The proportion that each DNA methylation context makes up of all DNA methylation indicates the predominance of specific DNA methylation pathways (Fig. 1d). The per-site DNA methylation level is the distribution of DNA methylation levels at individual methylated sites and indicates within a population of cells, the proportion that are methylated (Fig. 1e–g, Additional file 1: Figure S1). Symmetry is a comparison of per-site DNA methylation levels at cytosines on the Watson versus the Crick strand for the symmetrical CG and CHG contexts (Additional file 1: Figures S2 and S3). As CMT3 is responsible for maintaining the symmetrical DNA methylation of CHG sites [27], we can use *A. thaliana cmt3* mutants to establish thresholds with which to identify sites as symmetrical or asymmetrical and [58] quantify the asymmetry of mCHG sites (Additional file 1: Figure S4). Per-site DNA methylation and symmetry provide information into how well DNA methylation is maintained and how ubiquitously the sites are methylated across cell types within sequenced tissues [59].

There is extensive variation between species. Within each species, mCG had the highest levels of DNA methylation genome-wide (Fig. 1a, Additional file 2: Table S2). Between species, levels ranged as much as three-fold, from a low of ~30.5 % in *A. thaliana* to a high of ~92.5 % in *Beta vulgaris*. Levels of mCHG varied as much as approximately eight-fold between species, from only ~9.3 % in *Eutrema salsugineum* to ~81.2 % in *B. vulgaris* (Fig. 1b, Additional file 2: Table S2). mCHH levels were universally the lowest, but also the most variable with as much as an ~16-fold difference, the highest being ~18.8 % is in *B. vulgaris*. This was unusually high, as 85 % of species had less than 10 % mCHH and half had less than 5 % mCHH (Fig. 1c, Additional file 2: Table S2). The lowest mCHH level was found in *Vitis vinifera* with only ~1.1 % mCHH. mCG is the most predominant type of DNA methylation making up the largest proportion of the total DNA methylation in all examined species (Fig. 1d). *B. vulgaris* was a notable outlier, having the highest levels of DNA methylation in all contexts and having particularly high mCHH levels. The between-species variation observed was much greater than within species variation, when compared to *A. thaliana* accessions from the 1001 Epigenomes Project (Additional file 1: Figure S5) [60]. Multiple factors may be contributing to the differences between species observed, ranging from genome size and architecture, to differences in the activity of DNA methylation targeting pathways.

We examined these methylomes in a phylogenetic framework, which led to several novel findings and hypotheses regarding the evolution of DNA methylation pathways across flowering plants. In general, the Brassicaceae (mustard) family, which includes *A. thaliana*, has lower median levels of per-site mCHG methylation when compared to other species (Fig. 1f). Furthermore, symmetrical mCHG sites have a wider range of DNA methylation levels and increased asymmetry, whereas non-Brassicaceae species have very highly methylated symmetrical sites (Additional file 1: Figures S3 and S4b), suggesting that the CMT3 pathway is less effective in Brassicaceae genomes or that it operates in a cell-specific manner. This is further evidenced by *E. salsugineum*, with the lowest mCHG levels (Fig. 1b), which is a natural *cmt3* mutant, whereas *CMT3* is under relaxed selection in other Brassicaceae [61, 62]. Methylation of CG sites is also less well maintained in the Brassicaceae, with *Capsella rubella* showing the lower levels of per-site mCG methylation (Fig. 1e, Additional file 1: Figure S1).

Within the Fabaceae (legume) family, *Glycine max* and *Phaseolus vulgaris*, show considerably lower per-site mCHH levels as compared to *Medicago truncatula* and *Lotus japonicus*, even though they have equivalent levels of genome-wide mCHH (Fig. 1c and g). The Poaceae (grass) family, in general, have much lower levels of mCHH (~1.4–5.8 %), both in terms of total DNA methylation level and as a proportion of total methylated sites across the genome. Per-site mCHH level distributions varied, with species like *Brachypodium distachyon* having some of the lowest of all species, whereas others like *Oryza sativa* and *Z. mays* have levels comparable to *A. thaliana*. In *Z. mays*, CMT2 has been lost [31], and it may be that in other Poaceae, mCHH pathways are less efficient even though CMT2 is present. Collectively, these results indicate that different DNA methylation pathways may predominate in different lineages, with ensuing genome-wide consequences.

Several dicot species showed very low levels of mCHH (<2 %): *V. vinifera*, *Theobroma cacao*, *Manihot esculenta*, *Eucalyptus grandis*. No causal factor based on examined genomic features or examined DNA methylation pathways was identified; however, these plants are commonly propagated via clonal methods [63]. Among non-Poaceae species, the six lowest mCHH levels were found in species with histories of clonal propagation (Additional file 1: Figure S6). Effects of micropropagation on DNA methylation in *M. esculenta* using DNA methylation-sensitive amplified polymorphisms have been observed before [64], so has altered expression of methyltransferases due to micropropagation in *Fragaria* x *ananassa* (common garden strawberry) [65]. If repeated rounds of clonal propagation were responsible for low mCHH, we hypothesized that going through a single round of sexual reproduction might result in increased mCHH levels, as work in *A. thaliana* suggests that mCHH is re-established during reproduction [66, 67]. To test this hypothesis, we examined a DNA methylome of a parental *M. esculenta* plant that had previously undergone clonal propagation and a DNA methylome of its offspring that was germinated from seed. Additionally, the original *F. vesca* plant used for this study had been micro-propagated for four generations. We germinated seeds from these plants, as they would have undergone sexual reproduction and examined these as well. Differences were slight, showing little substantial evidence of genome-wide changes in a single generation of sexual reproduction (Additional file 1: Figure S7). As both of these results are based on one generation of sexual reproduction, it may be that this is insufficient to fully restore DNA methylation or that clonal propagation is not causal for the low levels of mCHH observed. This will require further studies of samples collected over multiple generations from matching lines that have been either clonally propagated or propagated through seed for numerous generations.

### Genome architecture of DNA methylation

**Fig. 2:**
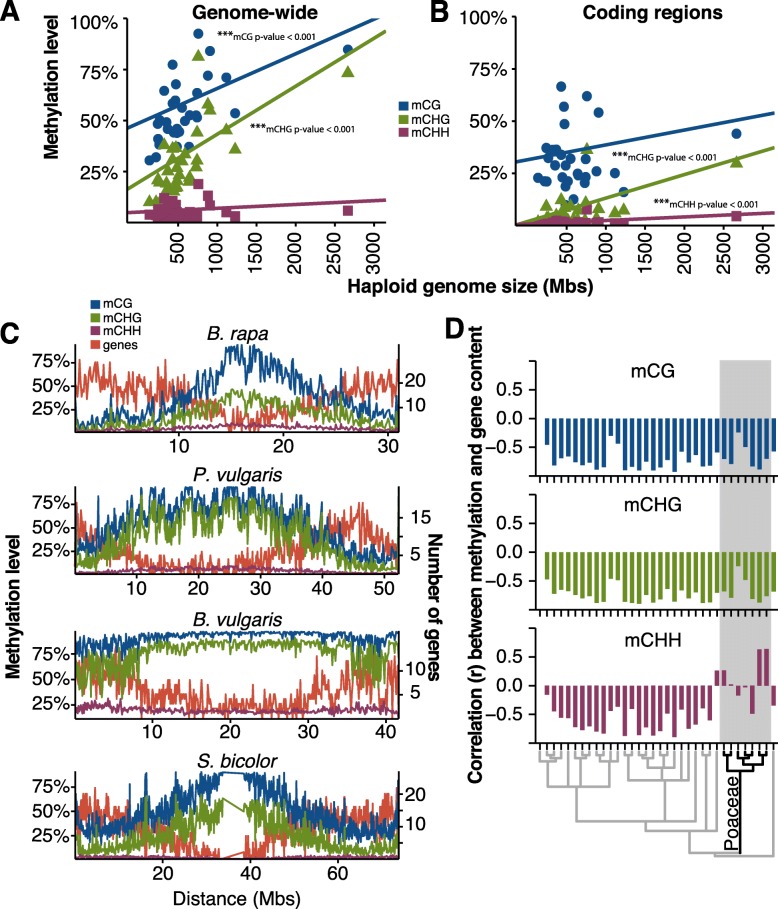
**a** Genome-wide methylation levels are correlated to genome size for mCG (*blue*) and mCHG (*green*), but not for mCHH (*maroon*). Significant relationships are indicated. **b** Coding region (CDS) methylation levels is not correlated to genome size for mCG (*blue*), but is for mCHG (*green*) and mCHH (*maroon*). Significant relationships are indicated. **c** *Chromosome plots* show the distribution of mCG (*blue*), mCHG (*green*), and mCHH (*maroon*) across the chromosome (100 kb windows) in relationship to genes. **d** For each species, the correlation (Pearson’s correlation) in 100 kb windows between gene number and mCG (*blue*), mCHG (*green*), and mCHH (*maroon*)

DNA methylation is often associated with heterochromatin. Two factors can drive increases in genome size, whole genome duplication (WGD) events, and in the copy number for repetitive elements. The majority of changes in genome size among the species we examined are due to changes in repeat content as the total gene number in these species only varies two-fold, whereas the genome size exhibits ~8.5-fold change. As genomes increase in size due to increased repeat content, it is expected that DNA methylation levels will increase as well. This was tested using phylogenetic generalized least squares (PGLS) [68] which takes into account the phylogenetic relationship and non-independence of species as more closely related species are more alike (Additional file 1: Table S3). Phylogenetic relationships were inferred from a species tree constructed using 50 single copy loci for use in PGLS (Additional file 1: Figure S8) [69]. A previous report had found a relationship between total methylation and genome size, but did not take into account the sequence context of that methylation [70]. Positive correlations were found between mCG and genome size (*p* value = 2.9 × 10^–3^) and between mCHG and genome size (*p* value = 2.2 × 10^–6^) (Fig. 2a), but no correlation was found with mCHH and genome size (Fig. 2a). This dataset was limited to one larger genome greater than 2 Gb, *Z. mays*, so we tested the effect that this had on the results. After removal of *Z. mays*, genome-wide mCHG methylation remained correlated with genome size, whereas mCG and mCHH showed no correlation (Additional file 1: Figure S9).

Similarly, a relationship between genic methylation level and genome size in plants has also been previously reported [47]. We found that within coding sequences (CDS) methylation levels were correlated with genome size for both mCHG (*p* value = 5 × 10^–6^) and mCHH (*p* value = 1.4 × 10^–5^), but in contrast found no correlation for mCG (*p* value > 0.18) (Fig. 2b). This prior study included many non-Angiosperm species and a limited set of Angiosperms and had also found that the correlation with mCG disappeared after removal of non-Angiosperm species [47]. Our observed correlations between CDS methylation and genome size were strongly driven by the large genome of *Z. mays*, and after its removal, no correlation was observed for any methylation context (Additional file 1: Figure S9). These results and those others [47, 70] suggest that the relationship between DNA methylation, both across the genome and within genes, and genome size is still not fully resolved and will require more extensive studies to resolve.

The highest levels of DNA methylation are typically found in centromeres and pericentromeric regions [39, 40, 48]. The distributions of DNA methylation at chromosomal levels were examined in 100 kb sliding windows (Fig. 2c, Additional file 1: Figure S10). The number of genes per window was used as a proxy to differentiate euchromatin and heterochromatin. Both mCG and mCHG have negative correlations between DNA methylation level and gene number, indicating that these two DNA methylation types are mostly found in gene-poor heterochromatic regions (Fig. 2d). Most species also show a negative correlation between mCHH and gene number, even in species with very low mCHH levels like *V. vinifera*. However, several Poaceae species show no correlation or even positive correlations between gene number and mCHH levels. Only two grass species showed negative correlations, *Setaria viridis* and *Panicum hallii*, which fall in the same clade (Fig. 2d). This suggests that heterochromatic mCHH is significantly reduced in many lineages of the Poaceae.

The methylome will be a composite of methylated and unmethylated regions. We implemented an approach (see “Methods”) to identify methylated regions within a single sample to discern the average size of methylated regions and their level of DNA methylation for each species in each sequence context (Additional file 3: Figure S11). For most species, regions of higher DNA methylation are often smaller in size, with regions of low or intermediate DNA methylation being larger (Additional file 3: Figure S12). More small RNAs, in particular 24 nt siRNAs map to regions of higher mCHH methylation (Additional file 3: Figure S13) and these regions of high 24 nt siRNAs tend to be smaller in size (Additional file 3: Figure S14). This may be because RdDM is primarily found on the edges of transposons whereas other mechanisms predominate in regions of deep heterochromatin [31]. Using these results, we can make inferences into the architecture of the methylome.

mCHG and mCHH regions are more variable in both size and DNA methylation levels than mCG regions, as little variability in mCG regions was found between species (Additional file 3: Figure S11). For mCHG regions, the Brassicaceae differed the most having lower DNA methylation levels and *E. salsugineum* the lowest. This fits with *E. salsugineum* being a *cmt3* mutant and RdDM likely being responsible for residual mCHG [62]. However, the sizes of these regions are similar to other species, indicating that this has not resulted in fragmentation of these regions (Additional file 3: Figure S11). The most variability was found in mCHH regions. Within the Fabaceae, the bulk of mCHH regions in *G. max* and *P. vulgaris* are of lower DNA methylation in contrast to *M. truncatula* and *L. japonicus* (Additional file 3: Figure S11). As these lower methylated mCHH regions are larger in size (Additional file 3: Figure S12) and less targeted by 24 nt siRNAs (Additional file 3: Figure S13), it would appear that deep heterochromatin mechanisms, like those mediated by CMT2, are more predominant than RdDM in these species as compared to *M. truncatula* and *L. japonicus*. Indeed, the genomes of *G. max* and *P. vulgaris* are also larger than *M. truncatula* and *L. japonicus* (Additional file 2: Table S2). In the Poaceae, we also find that mCHH regions are more highly methylated, even though genome-wide, mCHH levels are lower (Additional file 3: Figure S11). This indicates that much of the mCHH in these genomes comes from smaller regions targeted by RdDM (Additional file 3: Figures S12 and S13), which is supported by RdDM mutants in *Z. mays* [42]. In contrast, previously discussed species like *M. esculenta*, *T. cacao*, and *V. vinifera* had mCHH regions of both low DNA methylation and small size which could indicate that effect of all mCHH pathways have been limited in these species (Additional file 3: Figure S12 and S13).

### DNA methylation of repeats

**Fig. 3:**
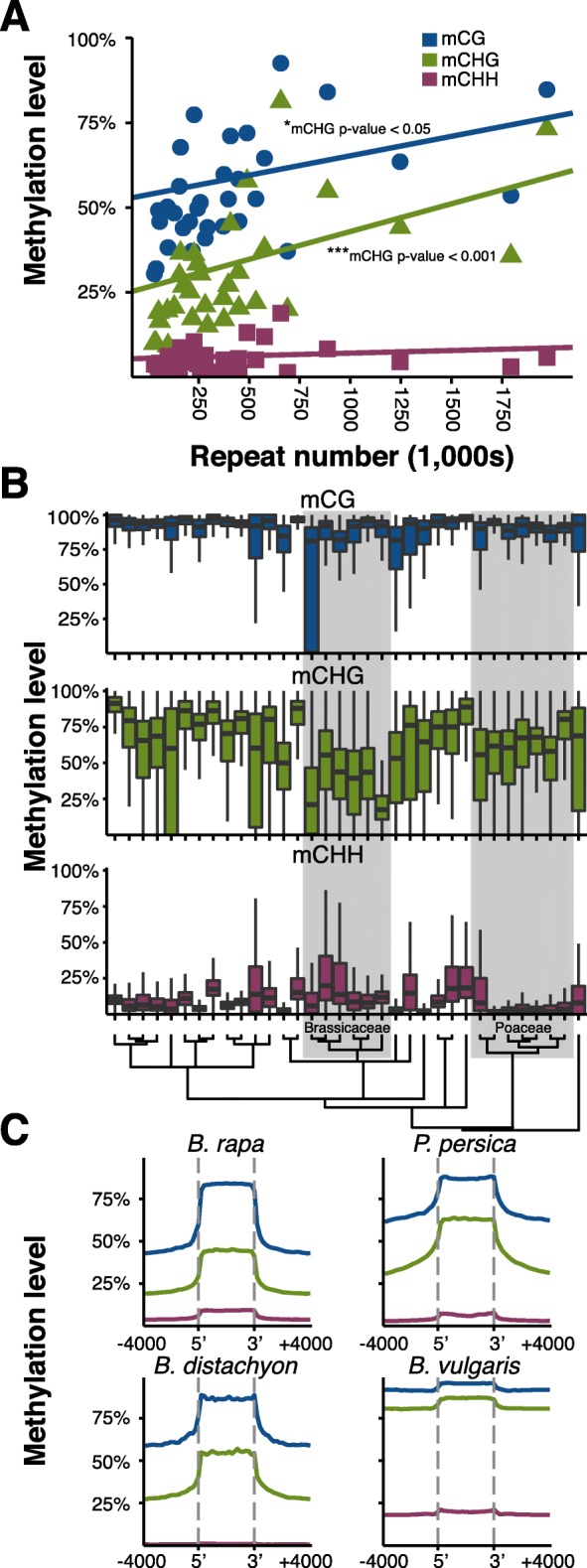
**a** Genome-wide methylation levels were correlated with repeat numbers for mCG (*blue*) and mCHG (*green*), but not for mCHH (*maroon*). Significant relationships are indicated. **b** Distribution of methylation levels for repeats in each species. **c** Patterns of methylation upstream, across, and downstream of repeats for mCG (*blue*), mCHG (*green*), and mCHH (*maroon*)

Genome-wide mCG and mCHG levels are related to the proliferation of repetitive elements. The extent which heterochromatin and repeats are represented among the genomes studied does vary with the completeness of the assembled genomes. Despite this, however, correlations were found between repeat number and mCG (*p* value = 3.0 × 10^–2^) and mCHG levels (*p* value = 4.9 × 10^–4^) (Fig. 3a, Additional file 1: Table S3). This likely explains the correlation of DNA methylation with genome size, as large genomes often have more repetitive elements [71, 72]. No such correlation between mCHH levels and repeat numbers was found (*p* value = 1) (Fig. 3a). This was unexpected given that mCHH is generally associated with repetitive sequences in many plant species [32, 73]. Both CDS mCHG and mCHH correlated with the total number of repeats (*p* value = 8.7 × 10^–3^, *p* value = 1.5 × 10^–2^, respectively), but CDS mCG did not (*p* value = 1) (Additional file 3: Figure S15A). CDS mCHG and mCHH were also correlated with the presence of repeats within gene bodies (exons, introns, and untranslated regions: mCHG *p* value = 1.6 × 10^–3^, mCHH *p* value = 2.0 × 10^–3^), whereas mCG was not (*p* value = 1) (Additional files 1 and 3: Table S3 and Figure S15b). Plotting the percentage of genes containing repeats against the total number of repeats showed a relationship between the percentage of repeat content in genes and total number of repeats (*p* value = 2.4 × 10^–6^) (Additional file 3: Figure S15C). After *Z. mays*, *B. vulgaris* has the highest percentage of genes containing repeats, much more so than expected given the total repeat content. This may explain in part why it has the highest CDS methylation levels.

Considerable variation exists in DNA methylation patterns within repeats. Across all species, repeats were heavily methylated at CG sequences, but were more variable in CHG and CHH methylation (Fig. 3b). mCHG was typically high at repeats in most species, with the exception of the Brassicaceae, in particular *E. salsugineum*. Similarly, low levels of mCHH were found in most Poaceae. Across the body of the repeat, most species show elevated levels in all three DNA methylation sequence contexts as compared to outside the repeat (Fig. 3c, Additional file 3: S16). Again, several Poaceae species stood out, as *B. distachyon* and *Z. mays* showed little change in mCHH within repeats, fitting with the observation that mCHH is depleted in deep heterochromatic regions of the Poaceae.

### CG gene body methylation

DNA methylation within genes in all three contexts is associated with suppressed gene expression [33], whereas genes that are only mCG methylated within the gene body are often constitutively expressed genes [74–76]. We classified genes using a modified version of the binomial test described by Takuno and Gaut [45] into one of four categories: CG gene body methylated (hereafter gbM), mCHG, mCHH, and unmethylated (UM) (Additional files 3, 4, and 5: Figure S17 and Table S4). This approach enables a consistent and statistically based classification of genes, but cannot fully capture finer details such as the pattern of methylation. GbM genes are methylated at CG sites, but not at CHG or CHH. Non-CG contexts are often coincident with mCG, for example RdDM regions are methylated in all three contexts. We further classified non-CG methylated genes as mCHG genes (mCHG and mCG, no mCHH) or mCHH genes (mCHH, mCHG, and mCG). Genes with insignificant amounts of DNA methylation were classified as unmethylated.

**Fig. 4:**
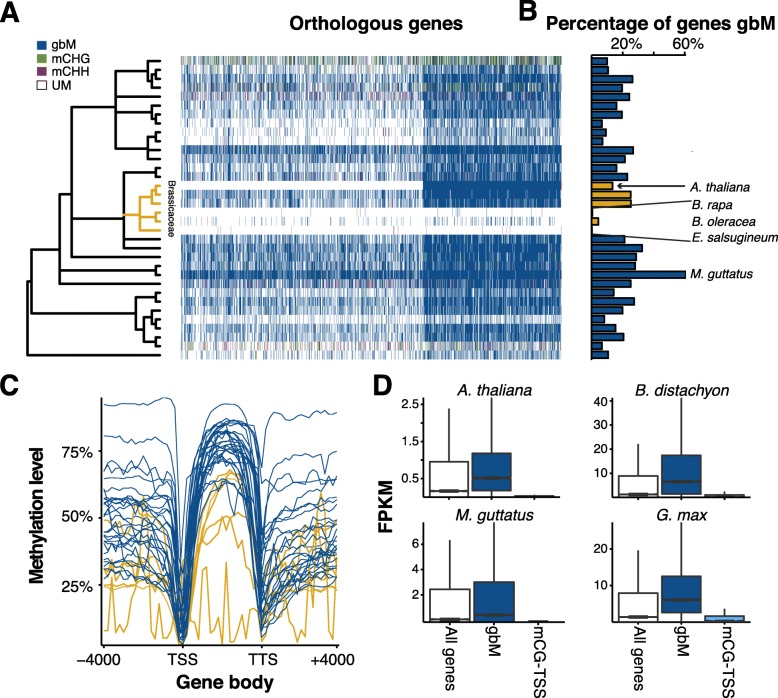
**a** *Heatmap* showing methylation state of orthologous genes (horizontal axis) to *A. thaliana* for each species (*vertical axis*). Species are organized according to phylogenetic relationship. **b** Percentage of genes in each species that are gbM (mCG only in coding sequences). The Brassicaceae are highlighted in *gold*. **c** The levels of mCG in upstream, across, and downstream of gbM genes for all species. Species in *gold* belong to the Brassicaceae and illustrate the decreased levels and loss of mCG. **d** gbM genes are more highly expressed, whereas mCG over the TSS (mCG-TSS) has reduced gene expression

Between species, the DNA methylation status of gbM can be conserved across orthologs [46]. The DNA methylation state of orthologous genes across all species was compared using *A. thaliana* as an anchor (Fig. 4a). *A. lyrata* and *C. rubella* are the most closely related to *A. thaliana* and also have the greatest conservation of DNA methylation status, with many *A. thaliana* gbM gene orthologs also being gbM genes in these species (~86.3 % and ~79.8 % of *A. thaliana* gbM genes, respectively). However, they also had many gbM genes that had unmethylated *A. thaliana* orthologs (~18.6 % and ~13.9 % of *A. thaliana* genes, respectively). Although gbM is generally “conserved” between species, this conservation breaks down over evolutionary distance with gains and losses of gbM in different lineages. In terms of total number of gbM genes, *M. truncatula* and *Mimulus guttatus* had the greatest number (Additional file 2: Table S2). However, when the percentage of gbM genes in the genome is taken into account (Fig. 4b), *M. truncatula* appeared similar to other species, whereas *M. guttatus* remained an outlier with ~60.7 % of all genes classified as gbM genes. The reason why *M. guttatus* has unusually large numbers of gbM loci is unknown and will require further investigation. In contrast, there has been considerable loss of gbM genes in *Brassica rapa* and *Brassica oleracea*, and a complete loss in *E. salsugineum*. This suggests that over longer evolutionary distance, the DNA methylation status of gbM varies considerably and is dispensable as it is lost entirely in *E. salsugineum*.

GbM is characterized by a sharp decrease of DNA methylation around the transcriptional start site (TSS), increasing mCG throughout the gene body and a sharp decrease at the transcriptional termination site (TTS) [75, 76]. GbM genes identified in most species show this same trend and even have comparable levels of DNA methylation (Fig. 4c, Additional file 3: Figure S18). Here, too, the decay and loss of gbM in the Brassicaceae is observed as *B. rapa* and *B. oleracea* have the second and third lowest DNA methylation levels, respectively, in gbM genes; and *E. salsugineum* shows no canonical gbM having only a few genes that passed statistical tests for having enrichment of mCG in gene bodies. As has previously been found [75, 76], gbM genes are more highly expressed as compared to UM and non-CG (mCHG and mCHH) genes (Fig. 4d, Additional file 3: Figure S19). The exception to this is *E. salsugineum* where the few genes that showed statistically significant amounts of mCG have almost no expression, supporting that they are not truly gbM genes but instead statistical anomalies associated with high numbers of statistical tests. A subset of unexpressed genes with mCG methylation was found, and in some cases, had higher mCG methylation around the TSS (mCG-TSS). Using previously identified mCG regions we identified genes with mCG overlapping the TSS, but lacking either mCHG or mCHH regions within or near genes. These genes had suppressed expression (Fig. 4d, Additional file 3: Figure S19) showing that although mCG is not repressive in gene-bodies, it can be when found around the TSS.

GbM genes are known to have many distinct features in comparison to UM genes. They are typically longer, have more exons, the observed number of CG dinucleotides in a gene are lower than expected given the GC content of the gene ([O/E]), and have previously been reported to evolve more slowly [45, 46]. We compared gbM genes to UM genes for each of these characteristics, using *A. thaliana* as the base for pairwise comparison for all species except the Poaceae where *O. sativa* was used (Additional files 3 and 6: Tables S5 and S6). With the exception of *E. salsugineum*, which lacks canonical gbM, these genes were longer and had more exons than UM genes (Additional files 3 and 6: Tables S5 and S6). Most gbM genes also had a lower CG [O/E] than UM genes, except for six species, four of which had a greater CG [O/E]. These included both *M. guttatus* and *M. truncatula*, which had the greatest number of gbM genes of any species. Recent conversion of previously UM genes to a gbM status could in part explain this effect. Previous studies have shown that gbM orthologs between *A. thaliana* and *A. lyrata* [45] and between *B. distachyon* and *O. sativa* [46] are more slowly evolving than UM orthologs. We verified this result for *A. thaliana* and *A. lyrata*. Within dicots, this result remains over short evolutionary distances, but it breaks down over greater distances with gbM genes typically evolving at equivalent rates as UM and, in some cases, faster rates (Additional files 3 and 6: Tables S5 and S6). Between *B. distachyon* and *O. sativa*, and across the Poaceae, we found the opposite result. GbM genes typically were evolving at faster rates (Additional files 3 and 6: Tables S5 and S6). To increase the robustness of our analyses across such diverse species, we incorporated several differences in our methods and choice of molecular evolution model, which could account for these discrepancies (see “Methods”). Why gbM genes are evolving at faster rates in the Poaceae is unknown and future studies will be needed to resolve this.

### Non-CG methylated genes

**Fig. 5:**
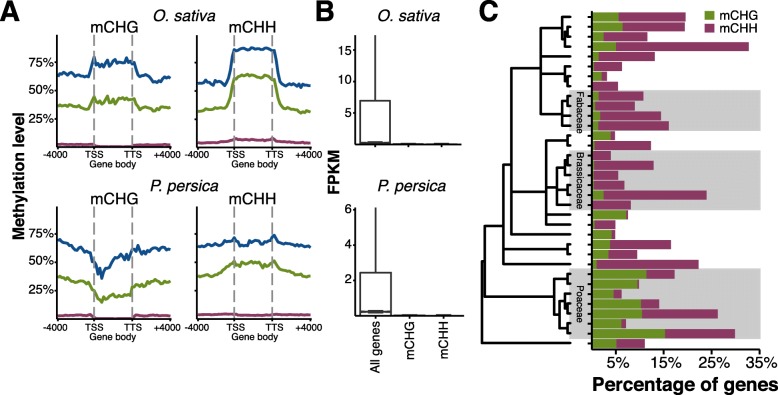
**a** Methylation levels for mCG (*blue*) (mCG only in coding sequences), mCHG (*green*) (mCG and mCHG in coding sequences), and mCHH (*maroon*) (mCG, mCHG, and mCHH in coding sequences) were plotted upstream, across, and downstream of mCHG and mCHH genes. **b** Gene expression of mCHG and mCHH genes vs. all genes. **c** The percentage of mCHG and mCHH genes per species. Species are arranged by phylogenetic relationship

Non-CG methylation exists within genes and is known to suppress gene expression [16, 18, 77–79]. Differences in annotation quality could lead to some transposons being misannotated as genes and thus as targets of non-CG methylation. However, work in both *A. thaliana* and *G. max* have shown that some percentage of protein-coding genes do indeed contain non-CG methylation [3, 12]. In many species there were genes with significant amounts of mCHG and little to no mCHH. High levels of mCHG within *Z. mays* genes is known to occur, especially in intronic sequences due in part to the presence of transposons [80]. Based on this difference in DNA methylation, mCHG and mCHH genes were maintained as separate categories (Additional files 4 and 5: Table S4). The DNA methylation profiles of mCHG and mCHH genes often resembled that of repeats (Fig. 5a, Additional file 3: Figure S18). Both mCHG and mCHH genes are associated with reduced expression levels (Fig. 5b, Additional file 3: Figure S19). As mCHG methylation is present in mCHH genes, this may indicate that mCHG alone is sufficient for reduced gene expression. It was also observed that *Cucumis sativus* has an unusual pattern of mCHH in many highly expressed genes, although this pattern was not observed in a second *C. sativus* sample and will require further study to understand the basis for this difference (Additional file 3: Figure S20). The number of genes possessing non-CG types of DNA methylation ranged from as low as ~3 % of genes (*M. esculenta*) to as high as ~32 % of genes (*F. vesca*) (Fig. 5c). In all the Poaceae, mCHG genes made up at least ~5 % of genes and typically more. In contrast, mCHG genes were relatively rare in the Brassicaceae where mCHH genes were the predominant type of non-CG genes.

Unlike gbM genes, there was no conservation of DNA methylation status across orthologs of mCHG and mCHH genes (Additional file 3: Figure S21). For many non-CG methylated genes, orthologs were not identified based on our approach of reciprocal best BLAST hit. For example, orthologs were found for only 488 of 999 of *A. thaliana* mCHH genes across all species. Previous comparisons of *A. thaliana*, *A. lyrata*, and *C. rubella* have shown no conservation of non-CG methylation between orthologs within the Brassicaceae [48]. However, we did observe some conservation based on gene ontology (GO). The same GO terms were often enriched in multiple species (Additional files 3 and 7: Figure S22 and Table S7). The most commonly enriched terms were involved in proteolysis, cell death, and defense responses; these processes could have profound effects on normal growth and development and may be developmentally or environmentally regulated. There was also enrichment in many species for genes related to electron-transport chain processes, photosynthetic activity, and other metabolic processes. Further investigation of these genes revealed that many are orthologs to chloroplast or mitochondrial genes, suggesting that they may be recent transfers from the organellar genome. The transfer of organellar genes to the nucleus is a frequent and ongoing process [81, 82]. Although DNA methylation is not found in chloroplast genomes, transfer to the nucleus places them in a context where they can be methylated, contributing to the mutational decay of these genes via deamination of methylated cytosines [83].

Transposable element insertions near or within genes can be one cause of non-CG methylated genes. To test this, we looked for enrichment of TEs upstream, within, and downstream of gbM, mCHG, and mCHH genes (Additional file 3: Figure S23). For the majority of species, TEs were indeed enriched near or within non-CG methylated genes. There were exceptions, however, as in both *S. lycopersicum* and *Z. mays*, there was no enrichment of TEs associated with these genes. Surprisingly, there was enrichment for TEs associated with gbM genes in many species. In large genomes like *Z. mays*, nearly every gene is associated with a TE in some way, indicating that the presence of an associated TE alone is not the only cause of non-CG methylation within genes.

### Non-coding sequences and regulatory regions

**Fig. 6:**
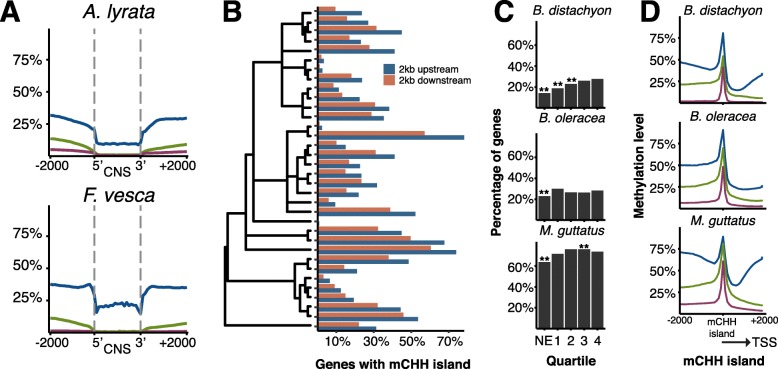
**a** Patterns of methylation across conserved non-coding sequences (CNS) for mCG (*blue*), mCHG (*green*), and mCHH (*maroon*). **b** Percentage of genes with mCHH islands 2 kb upstream or downstream. **c** Association of upstream mCHH islands with gene expression. Genes are divided into not-expressed (NE) and quartiles of increasing expression. ** indicates a difference in proportion from the fourth quartile at *p* < 0.01. **d** Patterns of upstream mCHH islands for mCG (*blue*), mCHG (*green*), and mCHH (*maroon*)

Outside of the gene body, DNA methylation might have an impact on gene expression through the DNA methylation of neighboring transcription factor binding sites (TFBS) or other regulatory elements. To date, there is limited in vivo evidence of such effects in plants, although the recent example of *repressor of silencing 1* (*ROS1*) hints at this possibility [84, 85]. In vitro evidence also supports the possibility of DNA methylation inhibiting and in some cases, promoting, transcription factor binding [86]. Conserved non-coding sequences contain many important regulatory elements, including TFBS [87, 88]. We identified CNS regions for a sample of species across the phylogeny and plotted DNA methylation levels (Fig. 6a, Additional file 3: Figure S24). DNA methylation in all three contexts was depleted across these regions, compared to outside. Locations of CNS regions were defined as either proximal (within 1 kbps), distal (>1 kbps), within untranslated regions (UTR), or within introns. Similar patterns were observed for CNS regions whether they were located proximally or distally to a gene (Additional file 3: Figure S24). UTR and intronic CNS sequences do show elevated levels of mCG in comparison, which might result from elevated mCG levels across the gene bodies of gbM genes. In *Z. mays*, high mCHH is enriched in the upstream and downstream regions of highly expressed genes and are termed mCHH islands [36, 37]. We identified mCHH islands 2 kb upstream and downstream of annotated genes for each species, finding that the percentage of genes with such regions varied considerably across species (Fig. 6b). Although some species other than *Z. mays* also show an association between mCHH islands and gene expression, many showed no such association, indicating no universal causal relationship between the two (Fig. 6c, Additional file 3: Figure S25). As has been observed previously in *Z. mays*, mCG and mCHG levels are generally higher on the distal side of the mCHH island to the gene (Fig. 6d, Additional file 3: Figure S26) [37]. However, this difference in DNA methylation level is much less pronounced in most other species as compared to *Z. mays* (Additional file 3: Figure S26). It is thought that these differences in DNA methylation on proximal versus distal sides of mCHH islands mark euchromatin-heterochromatin boundaries [37]. Indeed, mCHH islands are often associated with transposons [36, 37], however, there was no correlation found between the total number of repeats in the genome and the number of genes with mCHH islands (Additional files 1 and 3: Table S3 and Figure S27a). When correlated to the percentage of genes with repeats 2 kb upstream or downstream, both upstream and downstream mCHH islands are correlated (upstream *p* value = 4.5 × 10^–5^, downstream *p* value = 9.3 × 10^–4^) (Additional files 1 and 3: Table S3 and Figure S27b). While there was a correlation between the total repeat content and the percentage of upstream and downstream repeats (upstream *p* value = 1.3 × 10^–2^, downstream *p* value = 1.6 × 10^–3^), there were numerous outlying species which may explain the lack of correlation between mCHH islands and total repeat content (Additional files 1 and 3: Table S3 and Figure S27c). This supports a hypothesis that transposon distribution as opposed to transposon load alone is critical in shaping the epigenome.

## Discussion and conclusions

We present the methylomes of 34 angiosperm species in a phylogenetic framework using comparative epigenomics, which enables the study of DNA methylation in an evolutionary context. Extensive variation was found between species, both in levels of DNA methylation and distribution of DNA methylation, with the greatest variation being observed in non-CG contexts. The Brassicaceae show overall lower mCHG levels and reduced numbers of gbM genes, leading to a complete loss in *E. salsugineum*, that is associated with loss of CMT3 [62]. Whereas in the Poaceae, mCHH levels are typically lower than that in other species. The Poaceae have a distinct epigenomic architecture compared to eudicots, with mCHH often depleted in deep heterochromatin and enriched in genic regions. We also observed that many species with a history of clonal propagation tend to have lower mCHH levels, suggesting a potential effect. Epigenetic variation induced by propagation techniques can be of agricultural and economic importance [89], and understanding the effects of clonal propagation will require future studies over multiple generations. Evaluation of per-site DNA methylation levels, methylated regions, their structure, and association with small RNAs suggests that there are differences in the predominance of various molecular pathways.

Variation exists within features of the genome. Repeats and transposons show variation in their DNA methylation level and distribution with impacts on DNA methylation within genes and regulatory regions. Although gbM genes do show many conserved features, this breaks down with increasing evolutionary distance and as gbM is gained or lost in some species. GbM is known to be absent in the basal plant species *Selaginella moellendorffii* (lycophyte) [44], *Physcomitrella patens* (moss) [44], and *Marchantia polymorpha* (liverwort) [47]. That it has also been lost in the angiosperm *E. salsugineum* indicates that it is dispensable over evolutionary time [58]. Non-CG methylation shows no conservation at the level of individual genes, which indicates that it is gained and lost in a lineage specific manner. It is an open question as to the evolutionary origins of non-CG methylation within genes. This type of DNA methylation within in genes is typically associated with the presence of upstream or downstream TEs. However, species like *Z. mays* are an exception, with nearly every gene associated with TEs, suggesting that other causes might also exist. Many non-CG genes lack orthologous genes, which could indicate a preferential targeting of de novo genes, as in the case of the *qua-quine starch* (*QQS*) gene in *A. thaliana* [19]. At a higher order level, there appears to be a commonality in what categories of genes are targeted, as many of the similar functions are enriched across species. Other features, such as conserved non-coding sequences, and mCHH islands are also examined. CNS regions show depletion of all type of DNA methylation, hinting that DNA methylation may have an inhibitory role at regulatory regions. While mCHH islands are not conserved and show extensive variation that is associated with the distribution of repeats upstream and downstream of genes.

This study demonstrates that widespread variation in DNA methylation exists between flowering plant species. For many species, this is the first reported methylome and methylome browsers for each species have been made available to serve as a resource (http://schmitzlab.genetics.uga.edu/plantmethylomes). Historically, our understanding has come primarily from *A. thaliana*, which has served as a great model for studying the mechanistic nature of DNA methylation. However, the extent of variation observed previously [47, 48] and now shows that there is still much to be learned about underlying causes of variation in this molecular trait. Due to its role in gene expression and its potential to vary independently of genetic variation, understanding these causes will be necessary to a more complete understanding of the role of DNA methylation underlying biological diversity.

## Methods

### MethylC-seq and analysis

In plants, DNA methylation is highly stable between tissues and across generations [15, 48], showing little variation between replicates. DNA was isolated from leaf tissue and MethylC-seq libraries for each species were prepared as previously described [53]. Previously published datasets were obtained from public databases and reanalyzed [12, 15, 36, 48, 54–56, 62, 90]. Genome sequences and annotations for most species were downloaded from Phytozome 10.1 (http://phytozome.jgi.doe.gov/pz/portal.html) [51]. The *L. japonicus* genome was downloaded from the *Lotus japonicus* Sequencing Project (http://www.kazusa.or.jp/lotus/) [49], the *B. vulgaris* genome was downloaded from the *Beta vulgaris* Resource (bvseq.molgen.mpg.de/) [52], and the *C. sativa* genome from the *C. sativa* (*Cannabis*) Genome Browser Gateway (http://genome.ccbr.utoronto.ca/cgi-bin/hgGateway) [50]. As gene annotations for *S. viridis* were not available, gene models from the closely related *S. italica* were mapped onto the *S. viridis* genome using Exonerate [91] and the best hits retained. As repeat annotations were unavailable for 12 of the species studied, RepeatMasker [92] was used to annotate repetitive elements and transposons using plant repetitive element sequences downloaded from Repbase [93] and *A. thaliana* transposable element sequences [20].

Sequencing data for each species was aligned to their respective genome (Additional file 1: Table S1) [20, 49–52, 54, 94–116] and methylated sites called using previously described methods [117]. In brief, reads were trimmed for adapters and quality using Cutadapt [118] and then mapped to both a converted forward strand (all cytosines to thymines) and converted reverse strand (all guanines to adenines) using bowtie [119]. Reads that mapped to multiple locations and clonal reads were removed. The non-conversion rate (rate at which unmethylated cytosines failed to be converted to uracil) was calculated by using reads mapping to the lambda genome or the chloroplast genome if available (Additional file 1: Table S1). Cytosines were called as methylated using a binomial test using the non-conversion rate as the expected probability followed by multiple testing correction using Benjamini–Hochberg false discovery rate (FDR). A minimum of three reads mapping to a site was required to call a site as methylated. Data are available at the Plant Methylome DB http://schmitzlab.genetics.uga.edu/plantmethylomes.

### Phylogenetic tree

A species tree was constructed using BEAST2 [120] on a set of 50 previously identified single copy loci [69]. Protein sequences were aligned using PASTA [121] and converted into codon alignments using custom Perl scripts. Gblocks [122] was used to identify conserved stretches of amino acids and then passed to JModelTest2 [123, 124] to assign the most likely nucleotide substitution model.

### Genome-wide analyses

Genome-wide weighted methylation was calculated from all aligned data by dividing the total number of aligned methylated reads to the genome by the total number of methylated plus unmethylated reads [57]. To determine per-site methylation levels, the weighted methylation for each cytosine with at least 3 reads of coverage was calculated and this distribution plotted. Symmetry plots were constructed by identifying paired symmetrical cytosines with sequencing coverage and plotting the per-site methylation level of the cytosine on the Watson strand against the per-site methylation level of the Crick strand. An *A. thaliana cmt3* mutant was used to empirically determine the per-site methylation level at which symmetrical methylation disappeared [62] at 40 %. Methylated symmetrical pairs above this level were considered to be symmetrically methylated, while those below as asymmetrical. Correlations between methylation levels, genome sizes, and gene numbers were done in R and corrected for phylogenetic signal using the APE [125], phytools [126], and NLME packages assuming a model of Brownian motion. In total, 22 comparisons were conducted (Additional file 1: Table S3) and a *p* value < 0.05 after Bonferroni correction. Distribution of methylation levels and genes across chromosomes was conducted by dividing the genome into 100 kb windows, sliding every 50 kb using BedTools [127] and custom scripts. Pearson’s correlation between gene number and methylation level in each window was conducted in R. Weighted methylation levels for each repeat were calculated using custom python and R scripts.

### Methylated regions

Methylated regions were defined independent of genomic feature by methylation context (CG, CHG, or CHH) using BEDTools [127] and custom scripts. For each context, only methylated sites in that respective context were considered and used to define the region. The genome was divided into 25 bp windows and all windows that contained at least one methylated cytosine in the context of interest were retained. Windows were merged if they were within 100 bp of each other. The merged windows were then refined so that the first methylated cytosine became the new start position and the last methylated cytosine new end position. Number of methylated sites and methylation levels for that region was then recalculated for the refined regions. A region was retained if it contained at least five methylated cytosines and then split into one of four groups based on the methylation levels of that region: group 1, < 0.05 %; group 2, 5–15 %; group 3, 15–25 %; group 4, > 25 %. Size of methylated regions were determined using BedTools.

### Small RNA (sRNA) cleaning and filtering

Libraries for *B. distachyon*, *C. sativus*, *E. grandis*, *E. salsugineum*, *M. truncatula*, *P. hallii*, and *R. communis* were constructed using the TruSeq Small RNA Library Preparation Kit (Illumina Inc). Small RNA-sequencing (RNA-seq) datasets for additional species were downloaded from GEO and the SRA and reanalyzed [15, 54, 55, 101, 128, 129]. The small RNA toolkit from the UEA computational Biology lab was used to trim and clean the reads [130]. For trimming, 8 bp of the 3’ adapter was trimmed. Trimmed and cleaned reads were aligned using PatMan allowing for zero mismatches [131]. BedTools [127] and custom scripts were used to calculate overlap with mCHH regions.

### Gene-level analyses

Genes were classified as gbM, mCHG, or mCHH by applying a binomial test to the number of methylated sites in a gene [45] (Additional files 3, 4 and 5: Figure S17 and Table S4). The total number of cytosines and the methylated cytosines were counted for each context for the coding sequences (CDS) of the primary transcript for each gene. A single expected methylation rate was estimated for all species by calculating the percentage of methylated sites for each context from all sites in all coding regions from all species. We restricted the expected methylation rate to only coding sequences as the species study differ greatly in genome size, repeat content, and other factors that impact genome-wide methylation. Furthermore, it is known that some species have an abundance of transposons in UTRs and intronic sequences, which could lead to misclassification of a gene. A single value was calculated for all species to facilitate comparisons between species and to prevent setting the expected methylation level to low, as in the case of *E. salsugineum*, or to high, as in the case of *B. vulgaris*, which would further lead to misclassifications.

A binomial test was applied to each gene for each sequence context and *q*-values calculated by adjusting *p* values by Benjamini–Hochberg FDR. Genes were classified as gbM if they had reads mapping to at least 20 CG sites and has *q*-value < 0.05 for mCG and a *q*-value > 0.05 for mCHG and mCHH. Genes were classified as mCHG if they had reads mapping to at least 20 CHGs, a mCHG *q*-value < 0.05, and a mCHH *q*-value > 0.05. As mCG is commonly associated with mCHG, the *q*-value for mCG was allowed to be significant or insignificant in mCHG genes. Genes were classified as mCHH if they had reads mapping to at least 20 mCHH sites and a mCHH *q*-value < 0.05. *Q*-values for mCG and mCHG were allowed to be anything as both types of methylation are associated with mCHH. mCG-TSS genes were identified by overlap of mCG regions with the TSS of each gene and the absence of any mCHG or mCHH regions within the gene or 1000 bp upstream or downstream.

TEs mapping to within 2000 bp upstream, within, or 2000 bp downstream of a gene were identified using BedTools. GbM, mCHG, and mCHH genes were then tested for enrichment of TEs upstream, within, or downstream using Fisher’s exact test against the background of all the genes in a genome. GO terms for each gene were downloaded from phytozome 10.1 (http://phytozome.jgi.doe.gov/pz/portal.html) [51]. GO term enrichment was performed using the parentCHILD algorithm [132] with the F-statistic as implemented in the topGO module in R. Multiple testing correction was then applied using the Benjamini–Hochberg procedure. GO terms were considered significant with a *q*-value < 0.05.

### Exon number, gene length, and [O/E]

For each species, the general feature format 3 (gff3) file from phytozome 10.1 [51] was used to determine exon number and coding sequence length (base pairs, bp) for each annotated gene (hereafter referred to as CDS). Additionally, for each full length CDS (starting with the start codon ATG and ending with one of the three stop codons TAA/TGA/TAG), from the phytozome 10.1 [51] primary CDS fasta file, the CG [O/E] ratio was calculated, which is the observed number of CG dinucleotides relative to that expected given the overall G + C content of a gene. Differences for these genic features between gbM and UM genes were assessed using permutation tests (100,000 replicates) in R, with the null hypothesis being no difference between the gbM and UM methylated genes.

### Identifying orthologs and estimating evolutionary rates

Substitution rates were calculated between CDS pairs of monocots to *O. sativa* and dicots to *A. thaliana*. Reciprocal best BLAST with an e-value cutoff of ≤ 1E-08 was used to identify orthologs between dicot-*A. thaliana* and monocot-*O. sativa* pairs. Individual CDS pairs were aligned using MUSCLE [133], insertion-deletion (indel) sites were removed from both sequences, and the remaining sequence fragments were shifted into frame and concatenated into a contiguous sequence. A ≥ 30 bp and ≥ 300 bp cutoff for retained fragment length after indel removal and concatenated sequence length was implemented, respectively. Coding sequence pairs were separated into each combination of methylation (i.e. gbM-gbM and UM-UM). The *yn00* (Yang-Neilsen) [134] model in the program PAML for pairwise sequence comparison was used to estimate synonymous substitution rates, non-synonymous substitution rates, and adaptive evolution (*dS*, *dN*, and ω, respectively) [135]. Differences in rates of evolution between methylated and unmethylated pairs were assessed using permutation tests (100,000 replicates) in R, with the null hypothesis being no difference between the gbM and UM methylated genes.

### RNA-seq mapping and analysis

RNA-seq datasets [12, 15, 48, 54, 62, 101, 109, 129, 136–140] were downloaded from the Gene Expression Omnibus (GEO) and the NCBI Short Read Archive (SRA) for reanalysis. *B. distachyon* and *C. sativus* RNA-seq libraries were constructed using Illumina TruSeq Stranded mRNA Library Preparation Kit (Illumina Inc.) and sequenced on a NextSeq500 at the Georgia Genomics Facility. Reads were aligned using Tophat v2.0.13 [141] supplied with a reference genome feature file (GFF) with the following arguments -I 50000 --b2-very-sensitive --b2-D 50 (Additional file 1: Table S1). Transcripts were then quantified using Cufflinks v2.2.1 [142] supplied with a reference GFF.

### Conserved non-coding sequences

The CNS Discovery Pipeline 3.0 [88] was used to call conserved non-coding sequences through pair-wise comparison of closely related species (*A. lyrata-A. thaliana*, *B. distachyon-O. sativa*, *F. vesca-P. persica*, *G. raimondii-T. cacao*, *M. esculenta-P. trichocarpa*). As the genomes of some species analyzed here are as yet unpublished, we restricted our analysis to a representative subset of species with published genomes taken from across the phylogeny. CNS regions were defined as 5’ distal, 5’ proximal, intronic, 3’ proximal, and 3’ distal by the CNS Discovery Pipeline 3.0. Coordinates for CNS regions were extracted and methylation levels calculated across 2 kb upstream, across the CNS, and 2 kb downstream. BED files of called CNS regions are available at GitHub (https://github.com/chadn737/Widespread-natural-variation-of-DNA-methylation-within-angiosperms).

### mCHH islands

mCHH islands were identified for both upstream and downstream regions as previously described [37]. Briefly, methylation levels were determined for 100 bp windows across the genome. Windows of 25 % or greater mCHH with at least five methylated CHH sites, were identified 2 kb upstream and downstream of genes. Genes with more missing data in more than half the neighboring windows were removed. Methylation levels were then plotted centered on the window of highest mCHH, extending 2 kb in both directions. Genes associated with mCHH islands were categorized as non-expressed (NE) or divided into one of four quartiles based on their expression level. Differences in the proportions of each expression quartiles were determined in a pair-wise manner using prop.test in R with *p* value < 0.01 [37].

## Additional files

Additional file 1: Supplemental Tables and Figures. **Table S1**, **Table S3**, and **Figures S1**–**10**. (PDF 16677 kb) Additional file 2: Table S2. Trait and feature information for each species. (XLSX 15 kb) Additional file 3: Supplemental Table and Figures. **Table S5** and **Figures S11**-**27**
Additional file 4: Table S4. Classification of gene methylation status: part A species *A. thaliana* through *L. japonicus*. (XLSX 10029 kb) Additional file 5: Table S4. Classification of gene methylation status: part B species *M. domestica* through *Z. mays*. (XLSX 10996 kb) Additional file 6: Table S6. Summary statistics for evolutionary analysis of gbM genes. (XLSX 31 kb) Additional file 7: Table S7. GO term enrichment for each gene class and the species for which that GO term is enriched in. (XLSX 68 kb)
