# Supplementary material for: Widespread natural variation of DNA methylation within angiosperms
Source: Genome Biol. 2016 Sep 27;17:194. doi: 10.1186/s13059-016-1059-0 (PMC5037628; doi:10.1186/s13059-016-1059-0)
Supplement: Additional file 1: — Supplemental Tables and Figures. Table S1, Table S3, and Figures S1–10. (PDF 16677 kb) [file 13059_2016_1059_MOESM1_ESM.pdf]

Table S1: Sample descriptions and alignment statistics

Sample (*previously published)	Accession	Genome version (Ref chr, C = chloro-plast, L = lambda)	Non-clonal uniqe reads	Non-conversion rate (%)	Coverage (per-strand)
**Methylomes**					
A. lyrata*	MN47	v1.0 (L)	44,841,137	0.32	10.9
A. thaliana*	Col-0	TAIR10 (C)	47,350,526	0.49	17.5
A. trichopoda*	Santa Cruz 75	v1.0 (L)	49,237,779	1.64	1.7
B. distachyon	Bd21	v2.1 (C)	64,662,118	0.57	11.9
B. oleracea	TO1000	v1.0 (L)	70,770,309	0.34	7.2
B. rapa	FPsc	FPsc V1.3 (C)	43,120,536	0.33	7.6
B. vulgaris	KWS2320	v1.1 (L)	33,057,401	0.51	4.4
C. clementina	Clemenules (haploid)	v1.0 (L)	34,028,800	0.47	5.6
C. rubella*	MTE	v1.0 (L)	28,247,233	0.35	10.6
C. sativa	Purple Kush	canSat3 (L)	94,348,984	0.51	8.8
C. sativus	Gy14	v1.0 (L)	18,452,977	0.49	4.5
E. grandis	BRAZUS1	v1.1 (L)	77,856,975	0.50	5.6
E. salsugineum	Shandong	v1.0 (L)	52,132,901	0.40	10.7
F. vesca	Hawaii 4	v1.1 (C)	134,885,832	0.31	28.1
G. max*	LD	w82.a2.v1 (C)	134,770,631	0.61	6.9
G. raimondii	Ulbrich JFW	v2.1 (L)	164,295,534	0.34	10.8
L. japonicus	Gifu	v2.5 (L)	34,194,946	0.45	5.4
M. domestica	Borkh	v1.0 (L)	78,811,790	0.29	4.5
M. esculenta	AM560-2	v4.1 (L)	38,260,723	0.48	3.3
M .guttatus	IM62	v2.0 (L)	26,280,831	0.34	4.1
M. truncatula	R108	Mt4.0v1 (L)	28,692,412	0.45	4.0
O. sativa*	Nipponbare	v7.0 (C)	105,357,143	3.78	14.2
P. hallii	FIL2	v0.5 (L)	66,499,977	0.46	6.0
P. persica	Lovell	v1.0 (C)	59,995,728	0.46	13.2
P. trichocarpa	Nisquilly	v3.0 (L)	92,546,206	0.35	10.9
P. virgatum	Kanlow	v1.1 (C)	70,817,983	0.53	2.9
P. vulgaris*	G19833	v1.0 (C)	182,535,499	0.53	17.5
R. communis	–	v0.1 (C)	26,157,232	1.95	3.3
S. bicolor	Btx623	v2.1 (C)	98,950,391	0.32	6.8
S. lyscopersicum*	Ailsa Craig	iTAG2.3 (C)	43,901,067	0.39	2.4
S. viridis	A10	*unpublished (L)	70,720,073	0.44	9.0
T. cacao	Matina	V1.1 (L)	35,260,841	0.39	5.1
V. vinifera	Pinot Noir	GENOSCOPE.12X (L)	68,430,347	0.45	7.0
Z. mays*	B73	AGPv3.21 6a (C)	58,603,275	1.99	1.4
**Other Methylomes**					
***C. sativus* (rep 2)**	Gy14	V1.0 (L)	58,567,429	0.14	10.8
***F. vesca* from seed**	Hawaii 4	V1.1 (C)	21,045,198	0.28	6.6
***M. esculenta* clonal parent**	–	V4.1 (L)	41,544,847	0.20	5.4
***M. esculenta* from seed**	–	V4.1 (L)	32,771,281	0.22	4.2

RNA-seq

Sample	Left reads	Percent mapped	Right reads	Percent mapped	Accession
A. lyrata	6,919,759	96.5	NA	NA	ERX534148
A. thaliana	136,916,207	96.1	136,916,207	94.3	SRX540755
A. trichopoda	37,466,208	95.5	37,466,208	94.3	PRJNA212863*
B. distachyon	25,869,724	80.8	NA	NA	This study
B. oleracea	10,744,753	78.4	10,744,753	85.1	SRX423924
B. rapa	26,046,712	89.8	NA	NA	This study
C. rubella	7,336,100	96.3	NA	NA	ERX534160
C. sativus	22,455,762	90.7	NA	NA	This study
E. salsugineum	48,362,506	96.6	NA	NA	This study
G. max	71,477,958	95.2	NA	NA	SRX216871
G. raimondii	34,233,729	94.1	34,233,729	93.5	SRX111367
M. guttatus	68,433,843	61.3	68,433,843	59.2	SRX125342
O. sativa	70,167,065	98.3	NA	NA	SRX151644
P. persica	21,018,886	89.4	21,018,886	82.8	SRX173254
P. trichocarpa	65,387,890	94.1	65,387,890	92.9	SRX377987
R. communis	17,968,366	90.9	17,968,366	86.3	ERX021378
S. lycopersicum	22,136,306	96.4	NA	NA	SRX118613
T. cacao	132,298,128	88.5	132,298,128	83.0	SRX278006
V. vinifera	20,165,610	81.1	20,165,610	81.3	SRX155385
Z. mays	24,525,101	92.0	NA	NA	SRX151744

small RNA-seq

Sample	Raw Reads	Mapped reads	Mapped hits	Accession
A. thaliana	7,514,197	2,971,154	10,137,546	SRX096398
A. trichopoda	20,551,508	6,373,751	30,632,050	SRX201184
B. distachyon	29,356,604	5,137,721	27,346,090	This study
C. sativus	111,071,989	7,954,356	17,979,983	This study
E. grandis	943,145	253,050	2,614,647	This study
E. salsugineum	36,907,790	8,866,995	132,271,615	This study
G. max	6,098,005	444,785	241,709,144	SRX020503
G. raimondii	14,852,027	2,624,609	48,884,484	SRX203410
M. truncatula	34,526,436	7,357,566	73,438,007	This study
O. sativa	20,075,564	5,820,101	73,059,577	SRX235848
P. hallii	41,217,064	11,004,696	160,311,702	This study
P. persica	1,263,170	416,949	10,638,008	SRX155591
P. trichocarpa	4,472,811	2,297,172	31,865,340	GSM717875
R. communis	3,016,878	991,552	27,346,090	This study
S. bicolor	5,979,996	2,873,154	259,177,389	GSM803128
S. lycopersicum	4,763,701	3,298,255	34,986,792	GSM803579
V. vinifera	3,810,622	1,931,228	10,597,523	GSM803800
Z. mays	3,796,402	1,875,523	376,581,742	GSM433620

Figure S1: Distribution of per-site methylation levels for mCG (blue), mCHG (green), and mCHH (maroon) for each species.

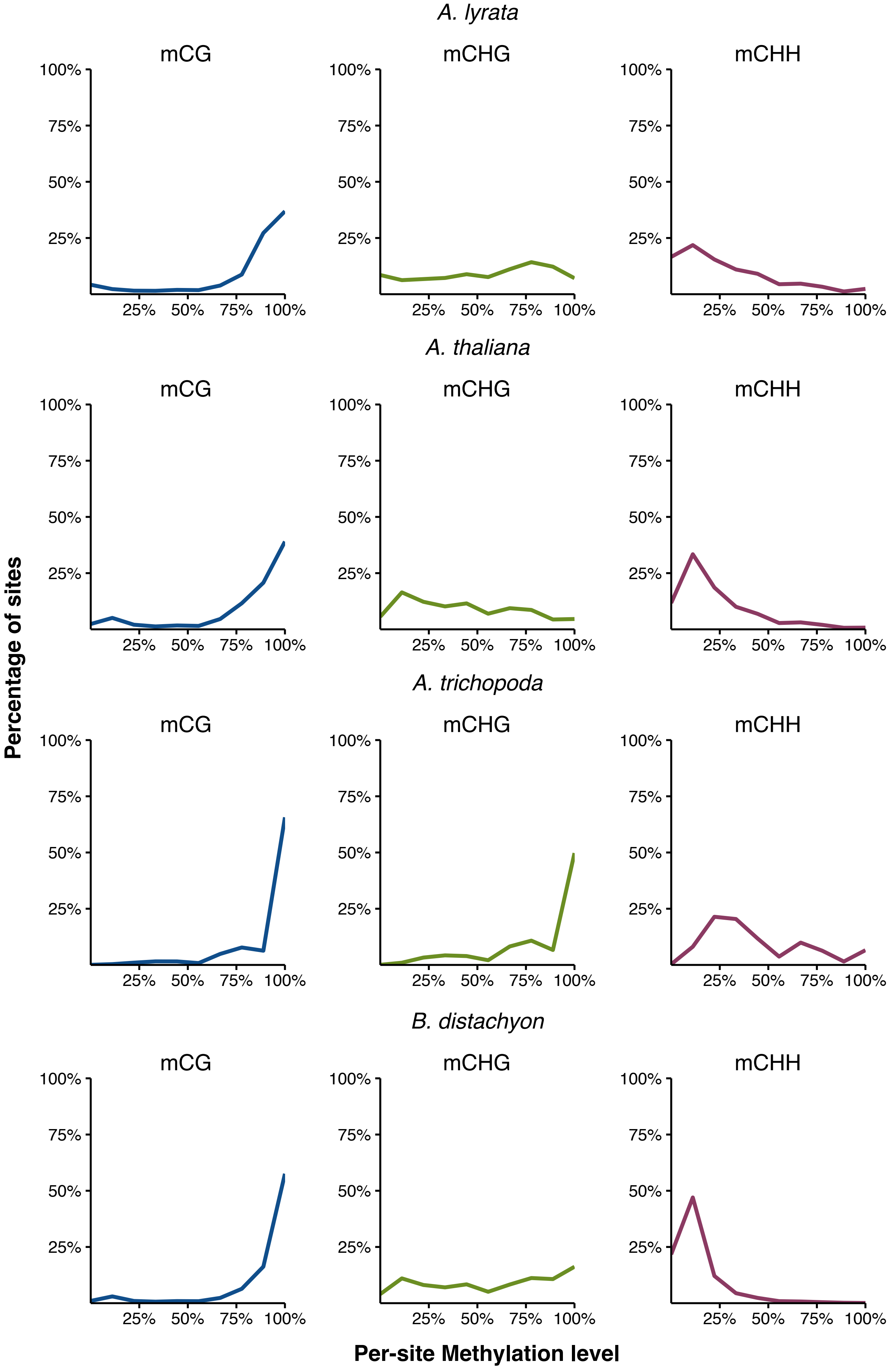

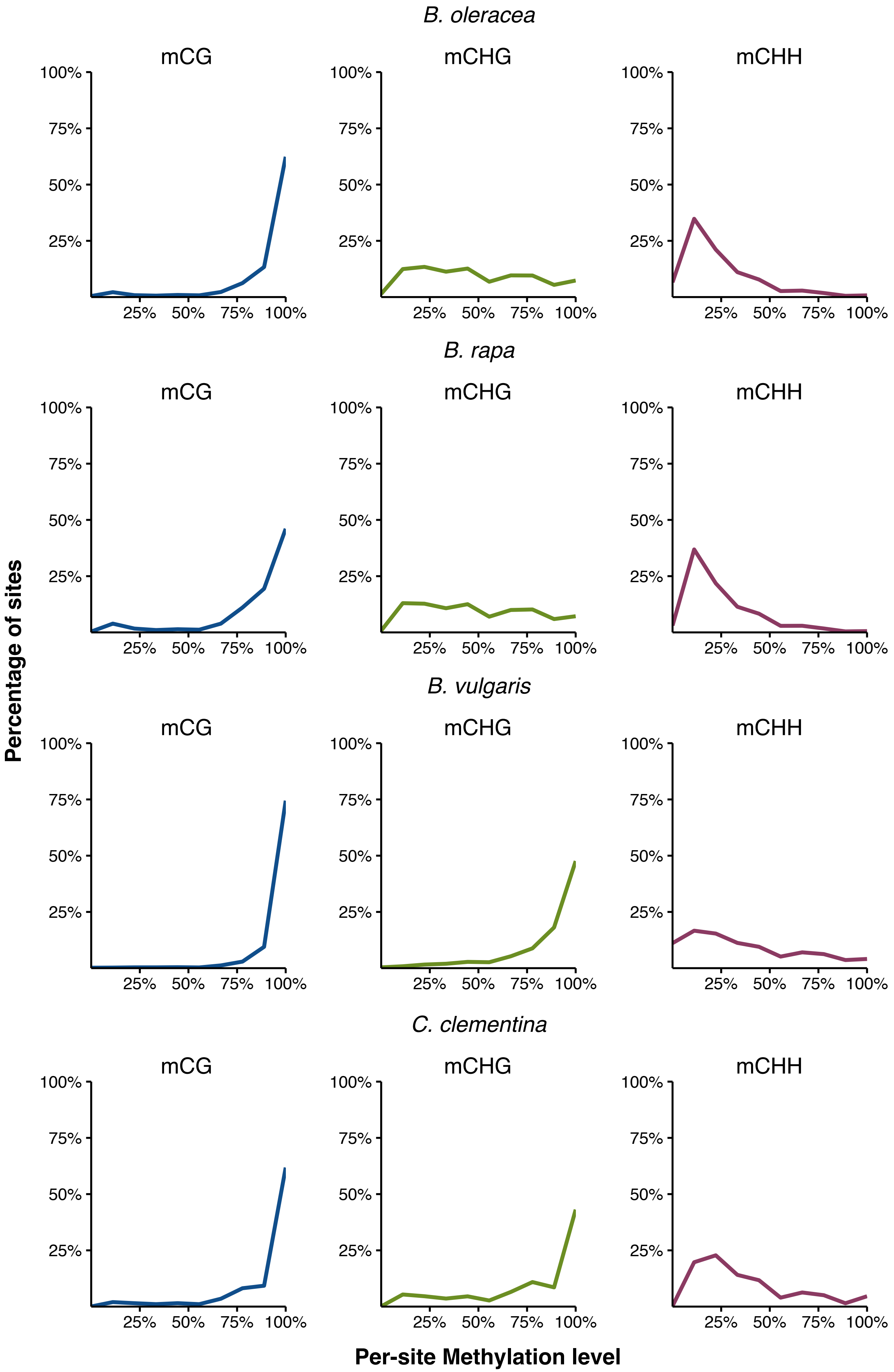

B. oleracea
B. rapa
B. vulgaris
C. clementina
mCG
mCHG
mCHH
100%
75%
50%
25%
Percentage of sites
Per-site Methylation level

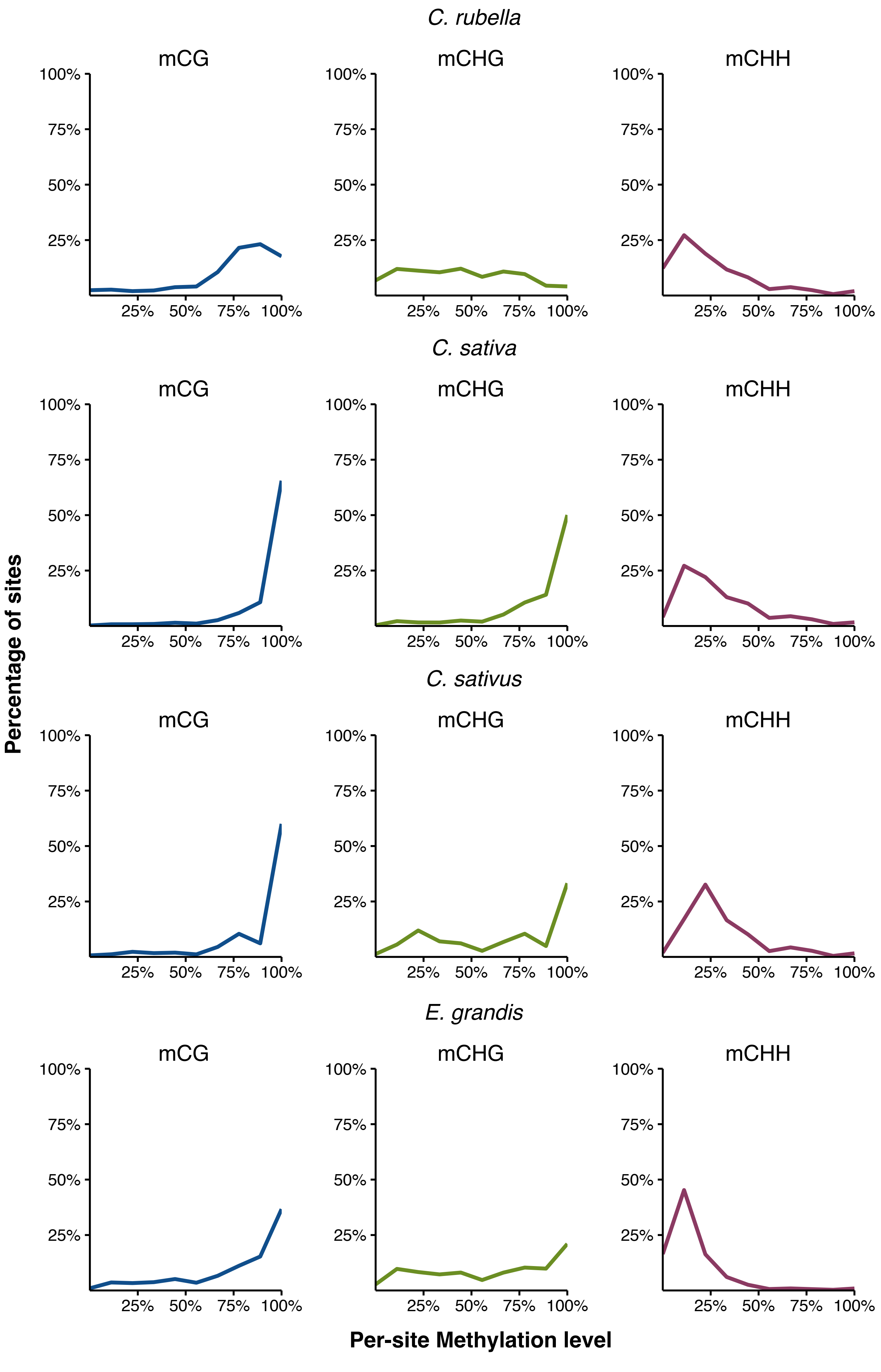

C. rubella
C. sativa
C. sativus
E. grandis
mCG
mCHG
mCHH
100%
75%
50%
25%
Percentage of sites
Per-site Methylation level

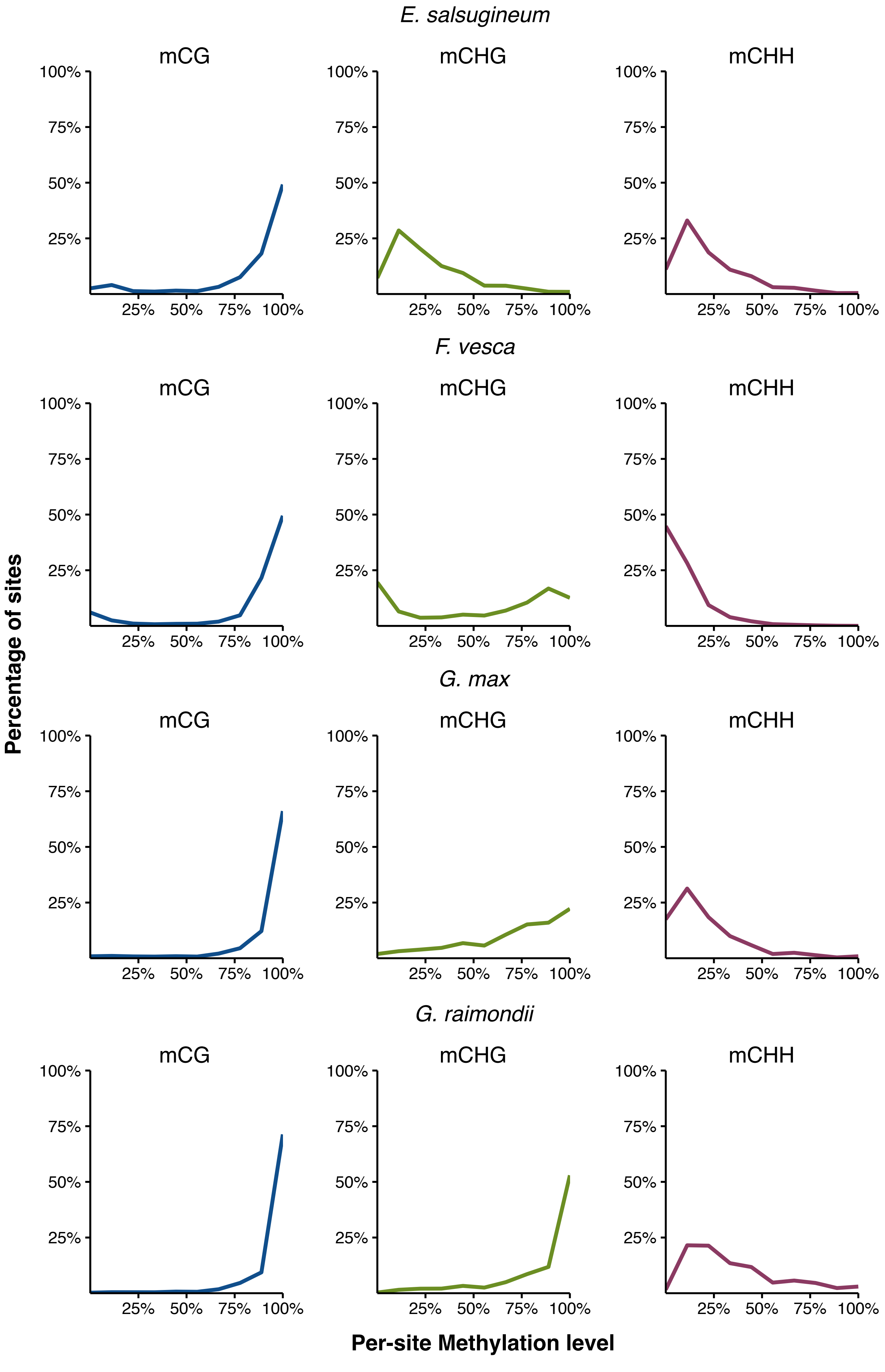
E. salsugineum
F. vesca
G. max
G. raimondii
mCG
mCHG
mCHH
100%
75%
50%
25%
Percentage of sites
Per-site Methylation level

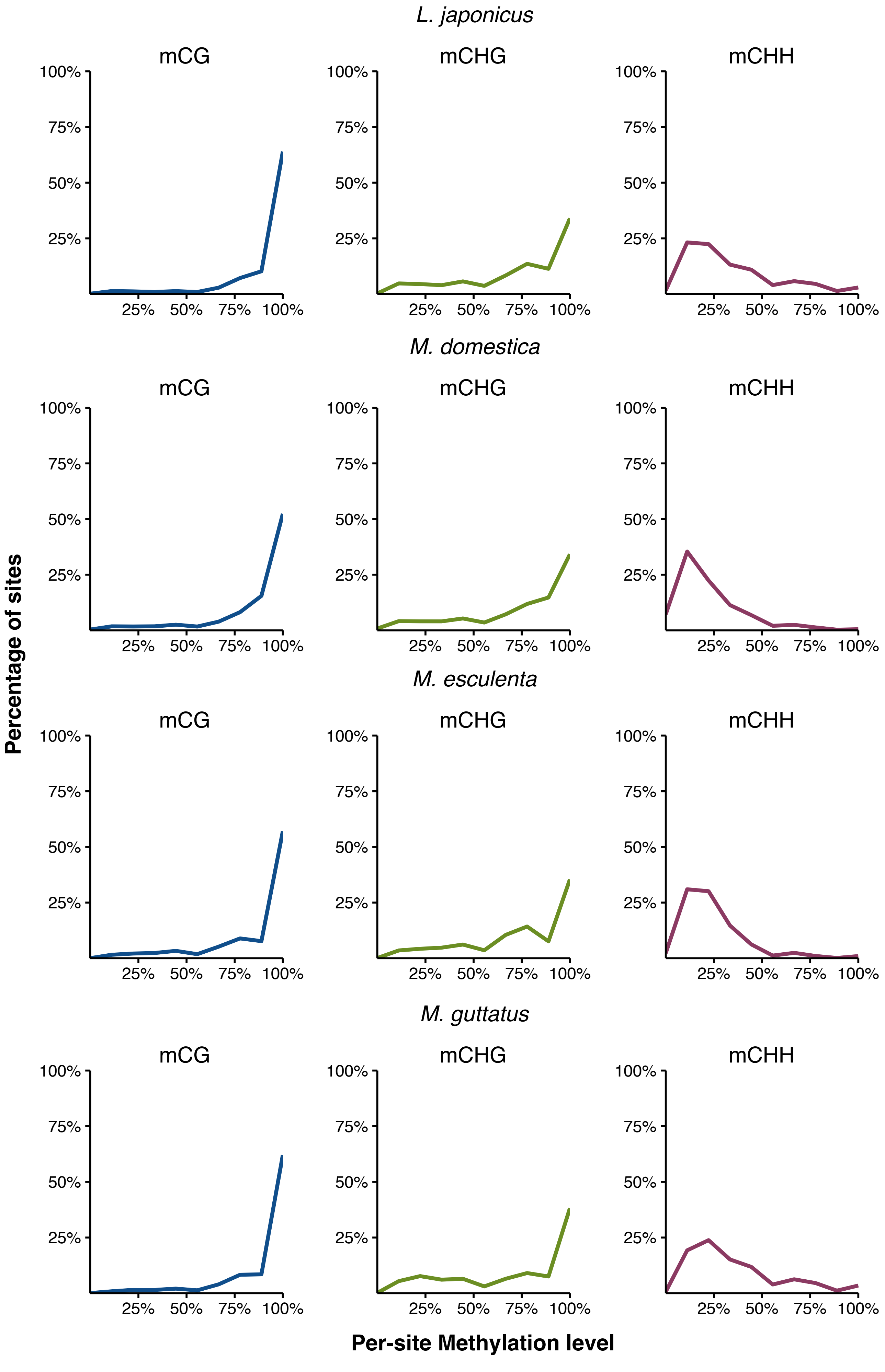

L. japonicus
M. domestica
M. esculenta
M. guttatus
mCG
mCHG
mCHH
100%
75%
50%
25%
Percentage of sites
Per-site Methylation level

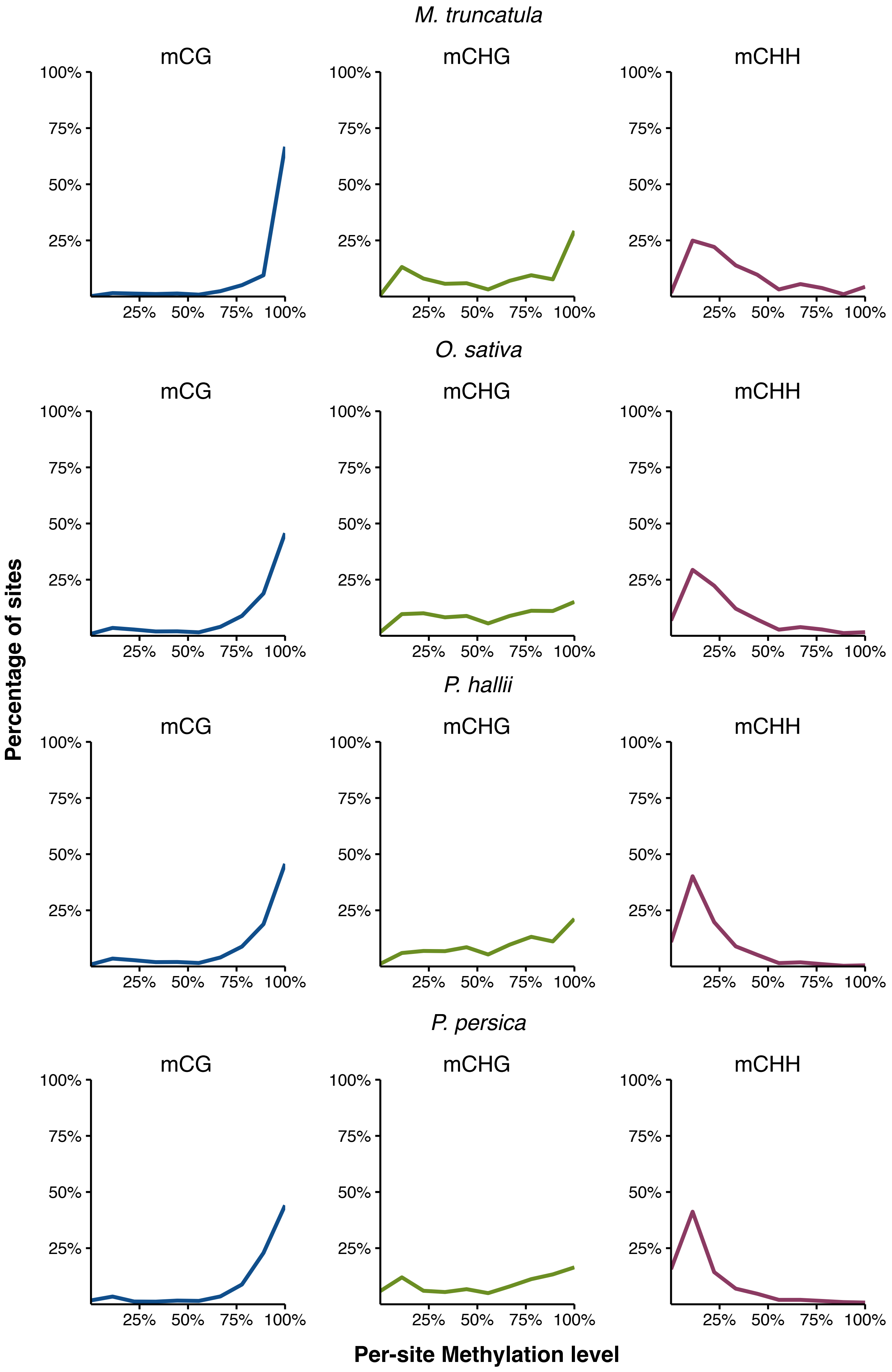
M. truncatula
O. sativa
P. hallii
P. persica
mCG
mCHG
mCHH
100%
75%
50%
25%
Percentage of sites
Per-site Methylation level

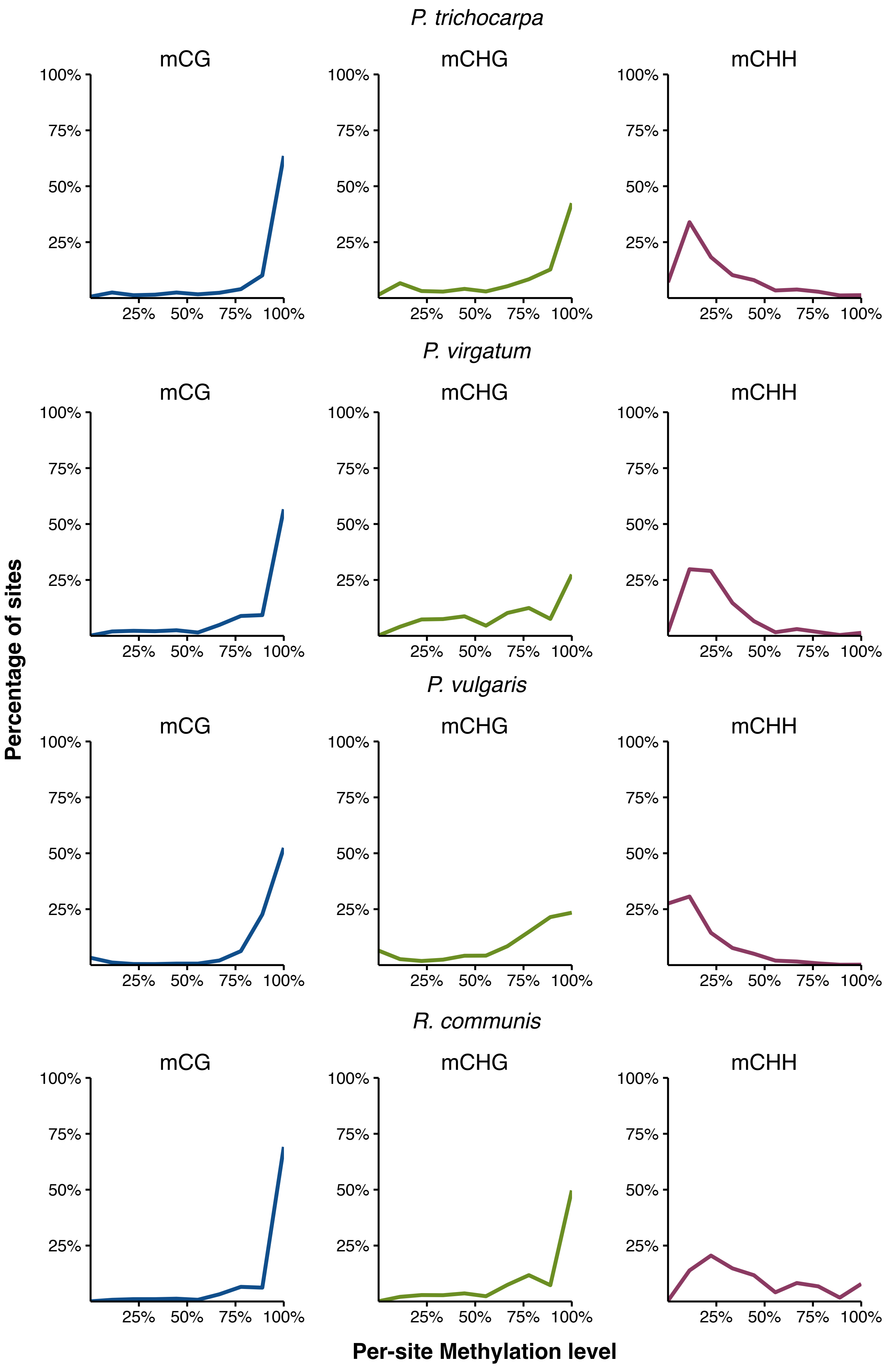
P. trichocarpa
P. virgatum
P. vulgaris
R. communis
mCG
mCHG
mCHH
100%
75%
50%
25%
Percentage of sites
Per-site Methylation level

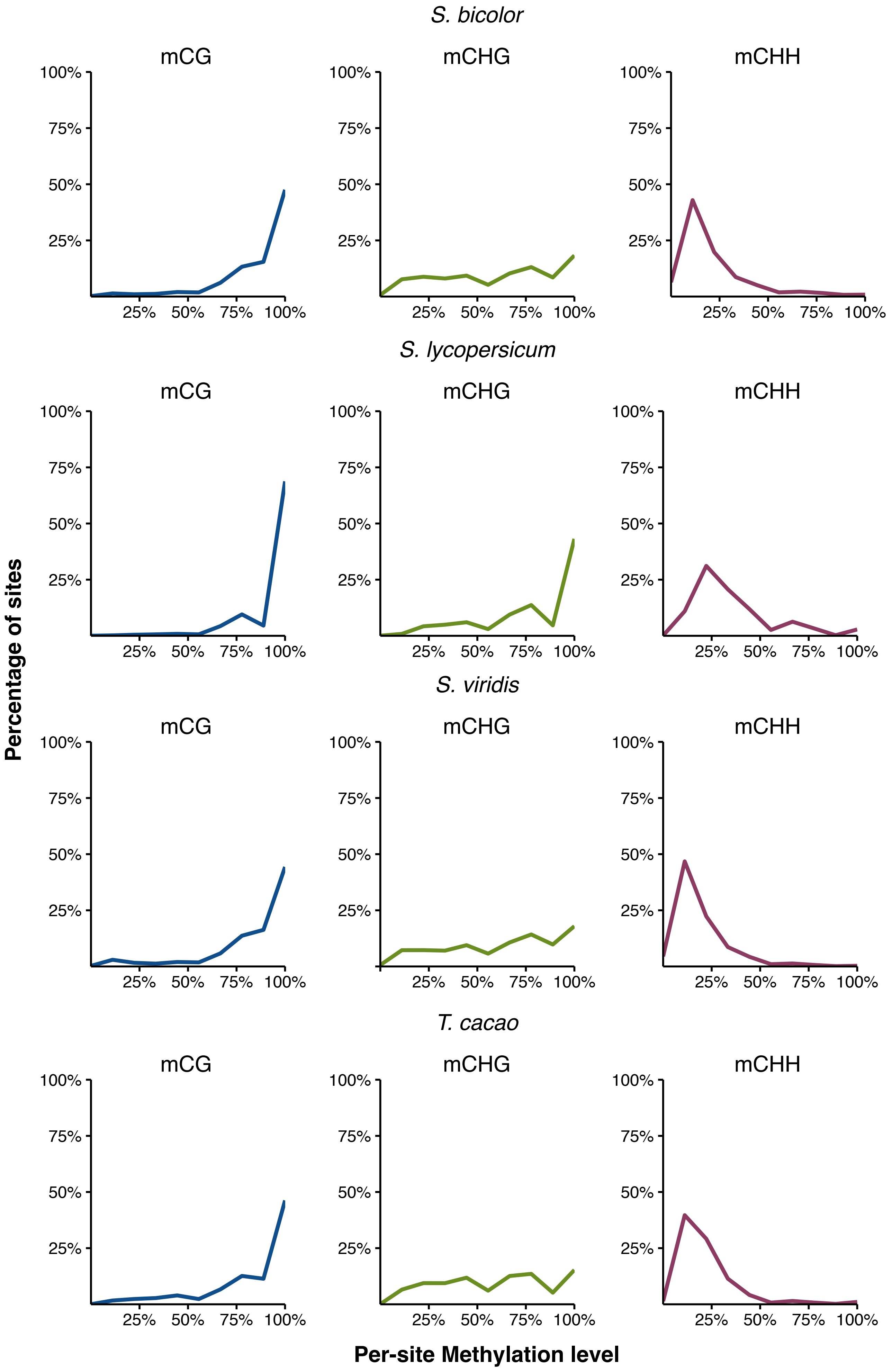
S. bicolor
S. lycopersicum
S. viridis
T. cacao
mCG
mCHG
mCHH
Percentage of sites
Per-site Methylation level
25%
50%
75%
100%

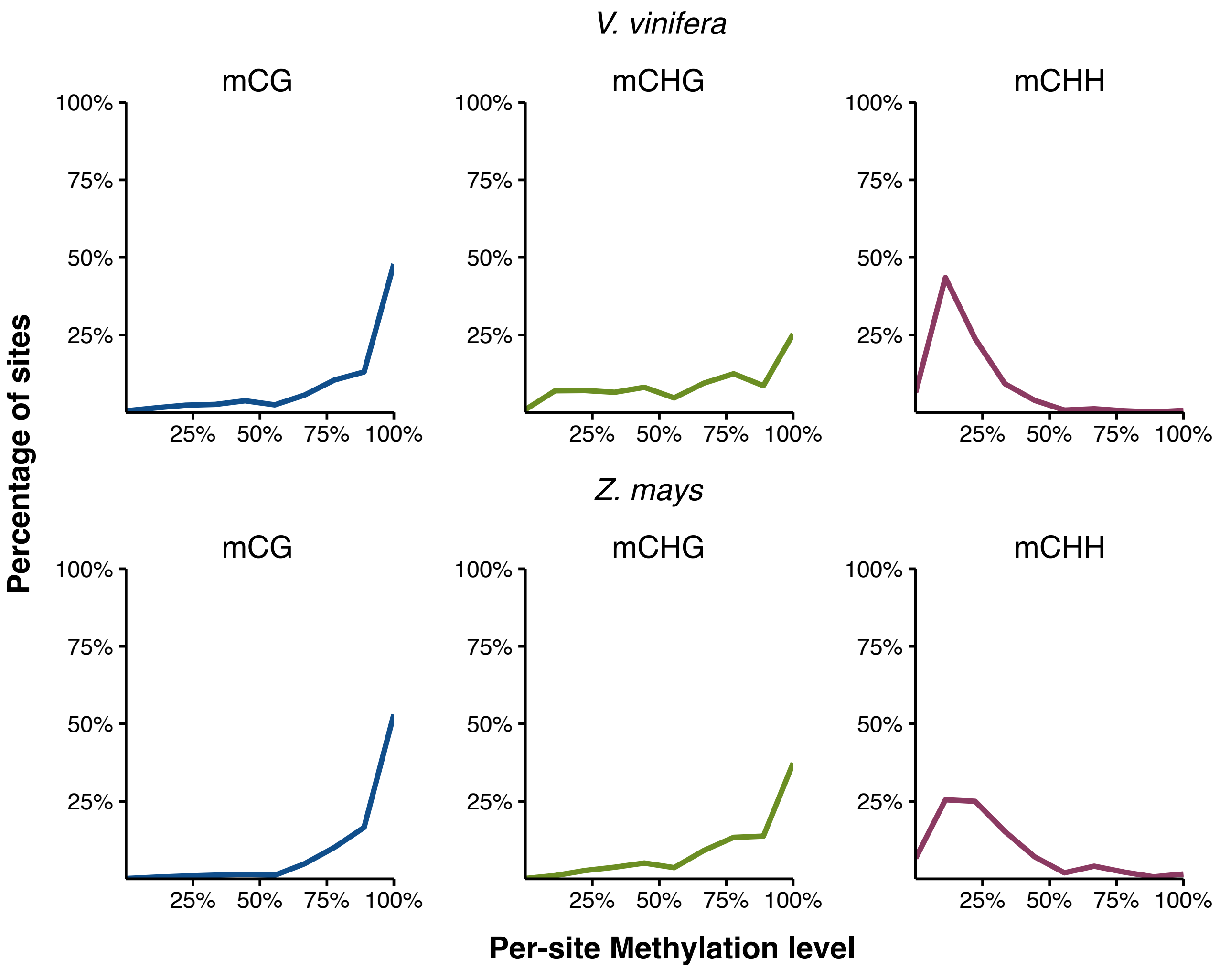
V. vinifera
Z. mays
mCG
mCHG
mCHH
mCG
mCHG
mCHH
100%
75%
50%
25%
25%
50%
75%
100%
Percentage of sites
Per-site Methylation level

Figure S2: Density plots showing the methylation levels of for the Watson strand (y axis) and the symmetrical site on the Crick strand (x axis) for mCG sites

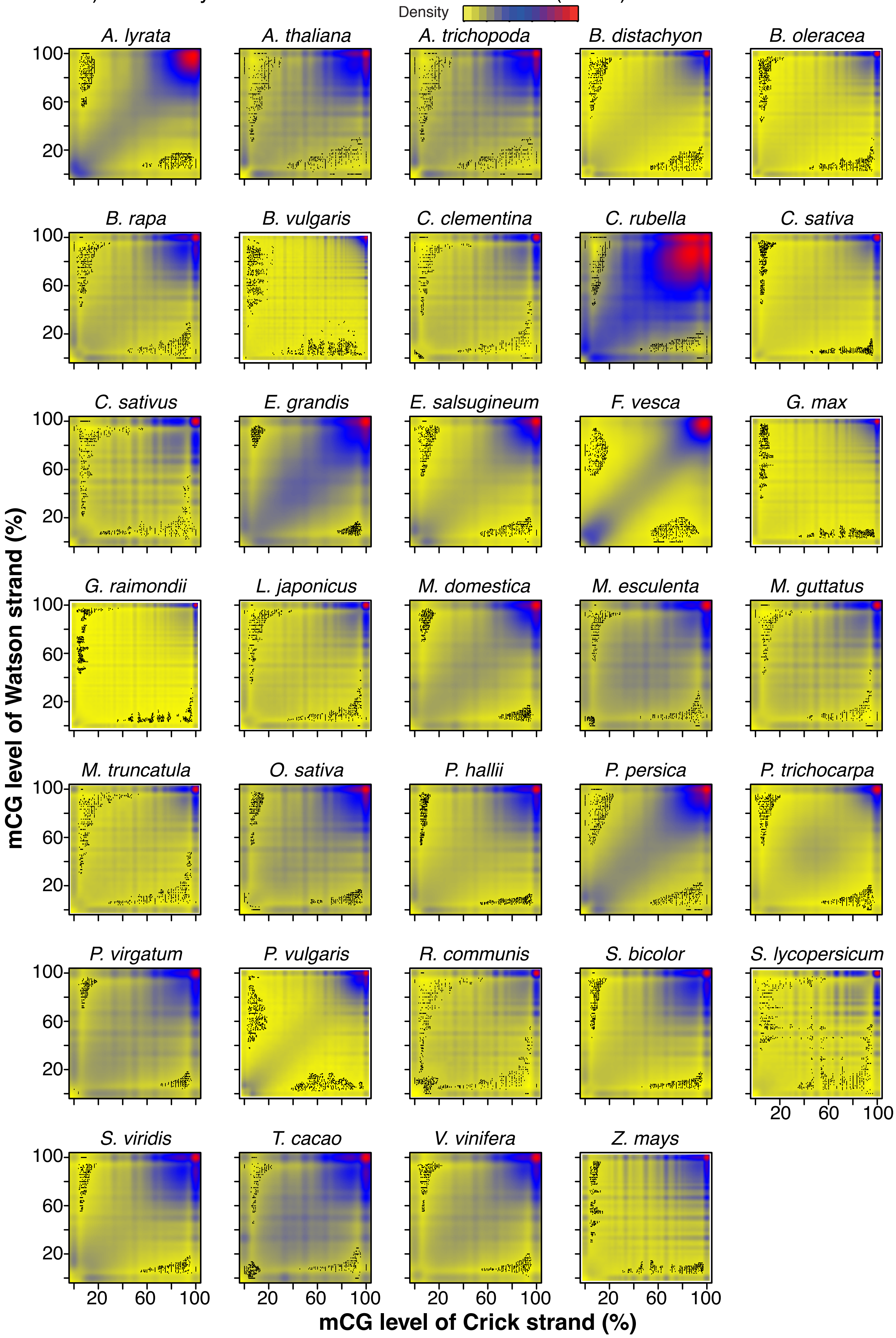

Figure S3: Density plots showing the methylation levels of for the Watson strand (y axis) and the symmetrical site on the Crick strand (x axis) for mCHG sites

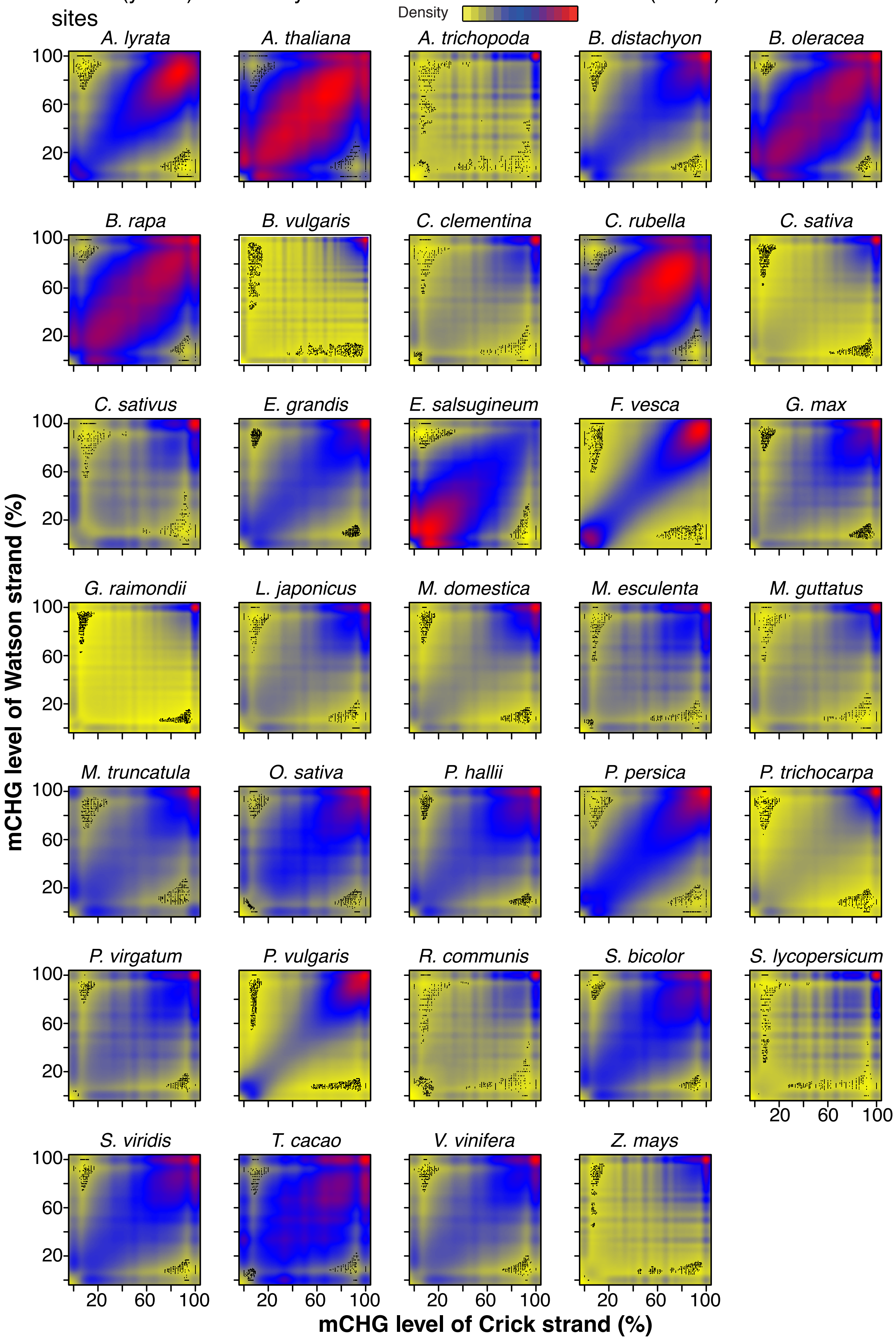

Figure S4: (A) Percentage of mCG and **(B)** mCHG sites where both strands are highly methylated (greater maintenance) or lowly methylated (less maintenance).

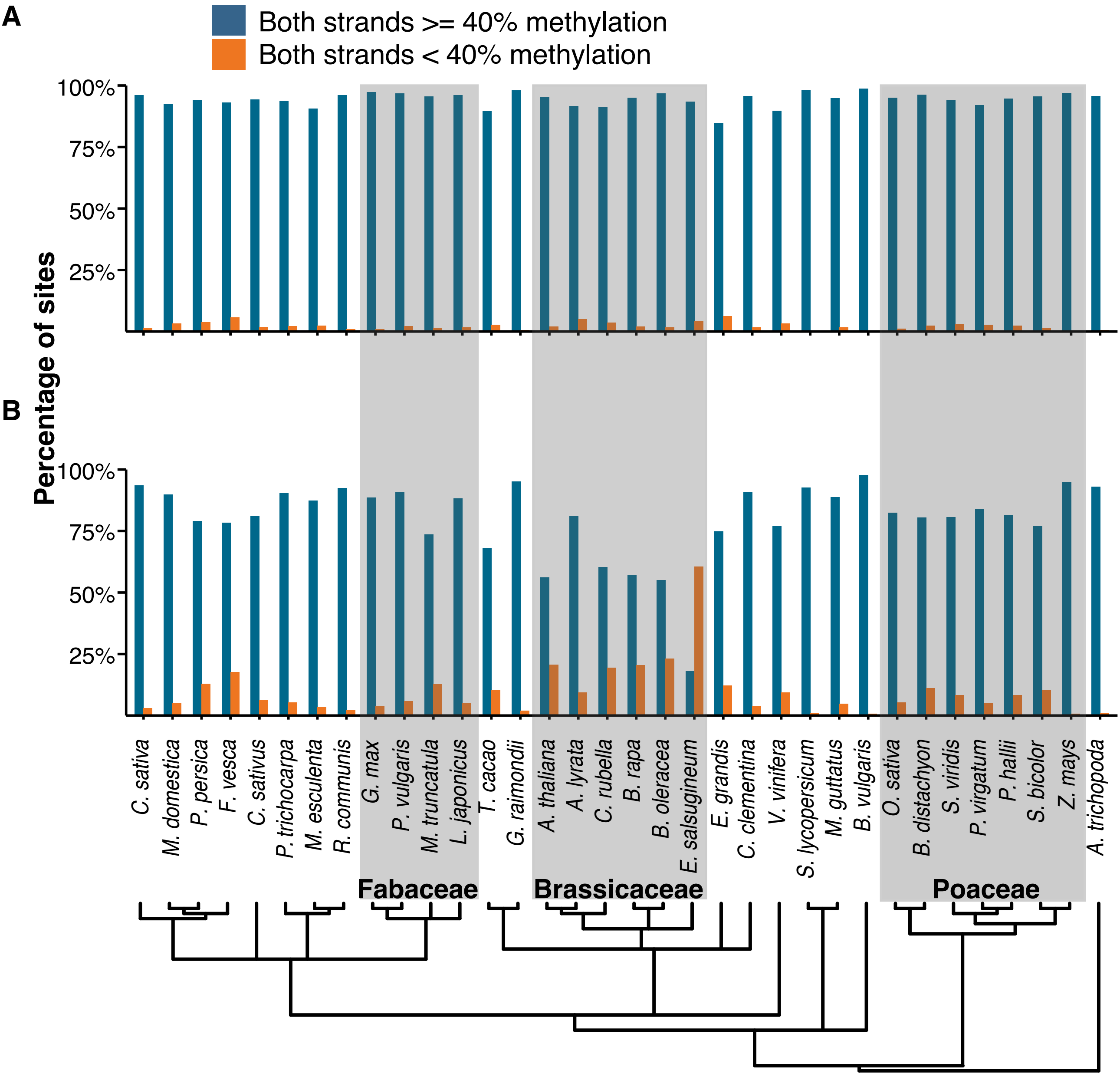

Figure S5: Distribution of between species genome-wide weighted methylation levels and variation within a species (*A. thaliana*) for mCG (blue), mCHG (green), and mCHH (maroon).

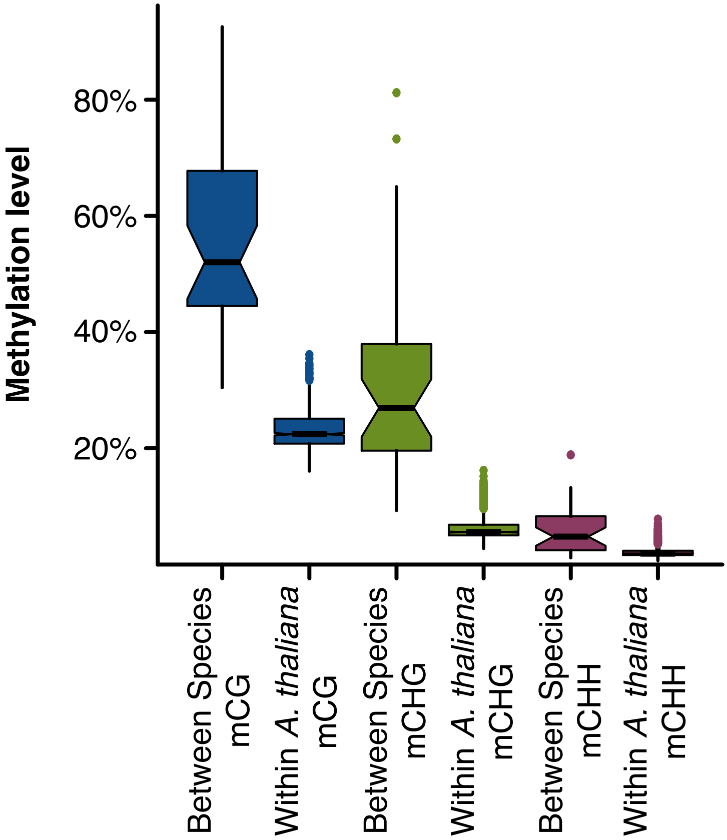

Figure S6: Non-Poaceae species ordered by mCHH levels, lowest to highest shows species with history of clonal propagation typically have lower mCHH

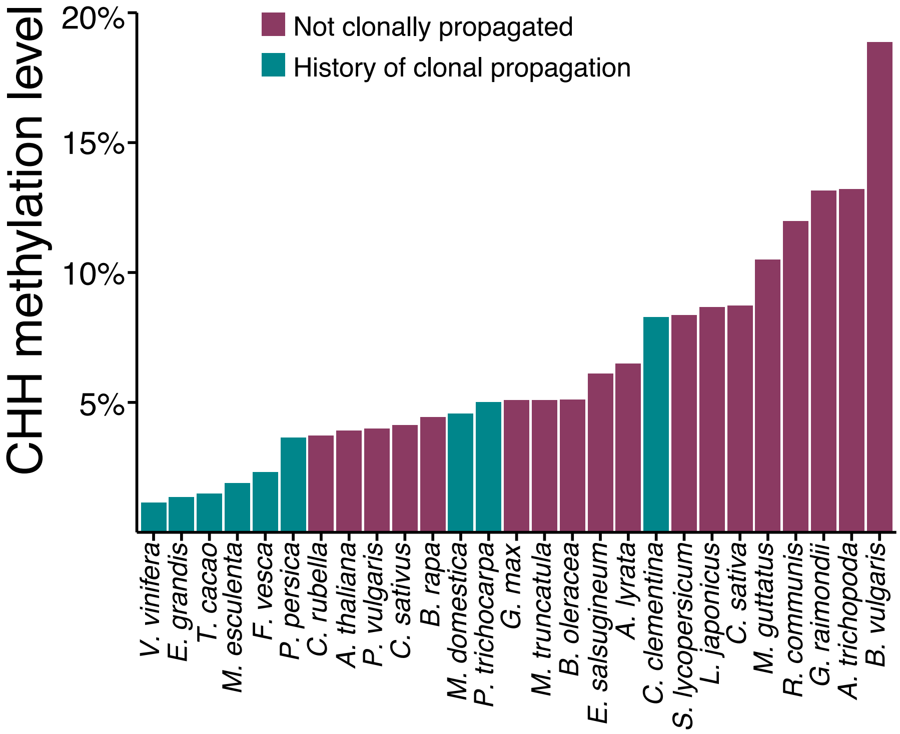

Figure S7: Genome-wide methylation levels for mCG, mCHG, and mCHH for:
(A) Clonally propagated *M. esculenta* parent and its offspring grown from seed
(B) *F. vesca* plant micro-propagated for four generations versus *F. vesca* plants grown from seed form the micro-propagated plants.

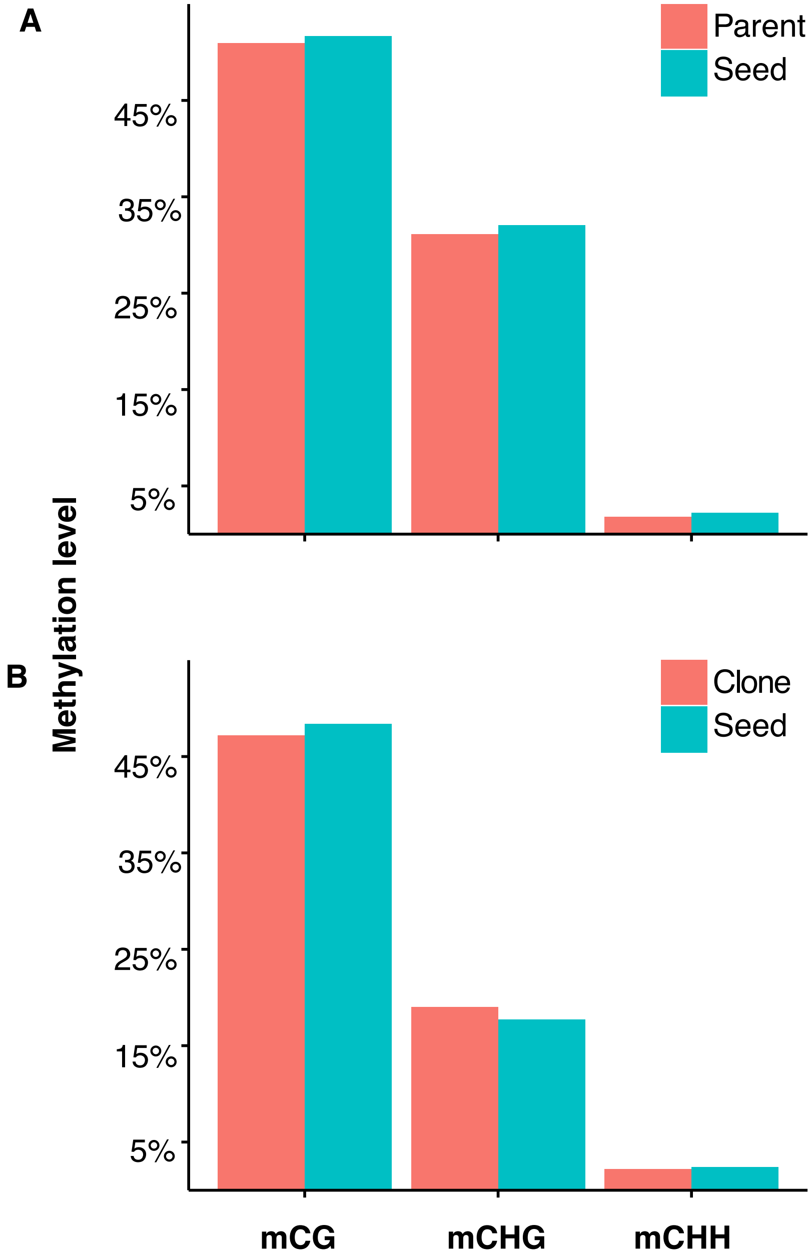

Table S3: Summary statistics of phylogenetic generalized least squares analysis for trait comparisons.

Trait 1	Trait 2	# Species	intercept	slope	p.value	Bonferonni corrected p.value
mCG	Genome Size	32	48.7504943064	0.0169713803	0.000006293281	0.000176211868
mCHG	Genome Size	32	19.7845475134572	0.0235156619966	0.0000000009482401	0.0000000265507228
mCHH	Genome Size	32	5.1366463153	0.0018633639	0.034725870994	0.972324387832
mCG no Z. mays	Genome Size no Z. mays	31	48.170625389	0.018085182	0.00952180922	0.26661065816
mCHG no Z. mays	Genome Size no Z. mays	31	19.626703378	0.023818846	0.00021896888	0.00613112864
mCHH no Z. mays	Genome Size no Z. mays	31	5.056775629	0.002016778	0.26311012732	1
mCG	Total Repeats	32	53.8018748258	0.000011465093	0.00108010637242	0.03024297842776
mCHG	Total Repeats	32	26.565366372543	0.000016365962	0.00001770899494	0.00049585185832
mCHH	Total Repeats	32	0.0000015241411	5.5704888631921	0.046973149450488	1
CDS mCG	Genome Size	32	31.518221381	0.007101638	0.06589852457	1
CDS mCHG	Genome Size	32	1.923988088448	0.011242486855	0.00000001022902	0.00000028641256
CDS mCHH	Genome Size	32	0.74579012384	0.00170169634	0.0000001573593	0.0000044060604
CDS mCG no Z. mays	Genome Size no Z. mays	31	38.68934257	-0.006672524	0.36419078659	1
CDS mCHG no Z. mays	Genome Size no Z. mays	31	5.787467575	0.003821584	0.14873836173	1
CDS mCHH no Z. mays	Genome Size no Z. mays	31	1.3391406289	0.0005619992	0.238485483242	1
CDS mCG	Total Repeats	32	34.527270063883	0.000002830528	0.40781587517441	1
CDS mCHG	Total Repeats	32	5.498494969133	0.000007093375	0.00031226428378	0.00874339994584
CDS mCHH	Total Repeats	32	1.2794580040238	0.0000010898888	0.000564409214761	0.015803458013308
CDS mCG	Percent Genic Repeats	32	28.067794	21.843326	0.05910463	1
CDS mCHG	Percent Genic Repeats	32	-0.748381503	26.714156372	0.00006036854	0.00169031912
CDS mCHH	Percent Genic Repeats	32	0.281218793	4.212898731	0.00007488527	0.00209678756
Percent Genic Repeats	Total Repeats	32	25.124029954336	0.000022730297	0.000000085798	0.000002402344
Percent Upstream CHH island	Total Repeats	32	35.121761749882	0.000003881292	0.42764391209097	1
Percent Downstream CHH island	Total Repeats	32	25.45655333597	0.00000461455	0.2394600490094	1
Percent Upstream Repeats	Total Repeats	32	44.087152442562	0.000018481661	0.00048329191607	0.01353217
Percent Downstream Repeats	Total Repeats	32	37.194219528330	0.000018383558	0.00006012372206	0.001683464
Percent Upstream CHH island	Percent Upstream Repeats	32	8.244775506	54.559977873	0.00005851275	0.000045878168
Percent Downstream CHH island	Percent Downstream Repeats	32	4.533068496	50.533338311	0.00003336713	0.0009342796

Figure S8: (A) Phylogenetic species tree constructed from 50 single copy loci. *C. sativa* was excluded due to insufficient data from these loci. **(B)** Phylogenetic species tree used for phylogenetic generalized least squares. A. trichopoda was excluded due to incorrect placement in the original species tree.

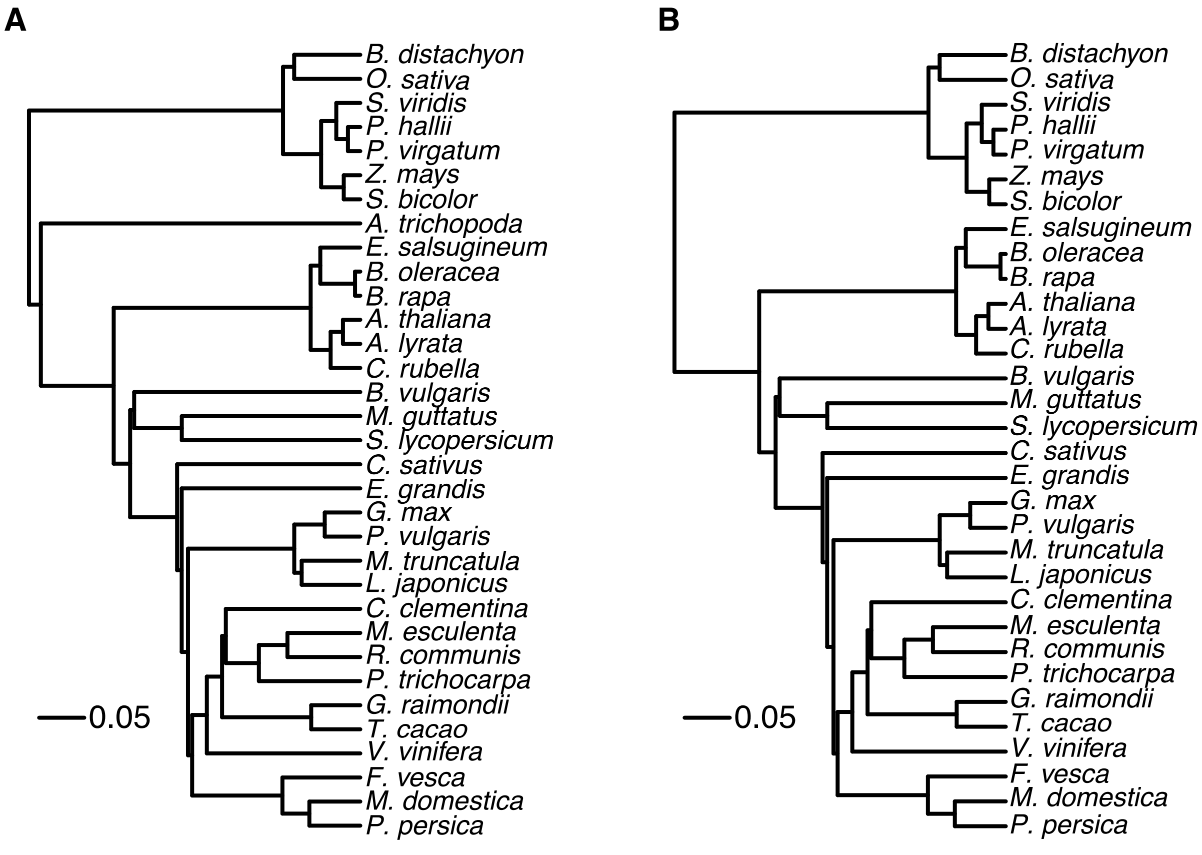

Figure S9: (A) Genome-wide mCG (blue), mCHG (green), mCHH (maroon) levels plotted against genome size with *Z. mays* removed. **(B)** mCG (blue), mCHG (green), mCHH (maroon) levels within coding sequences (CDS) plotted against genome size with *Z. mays* removed.

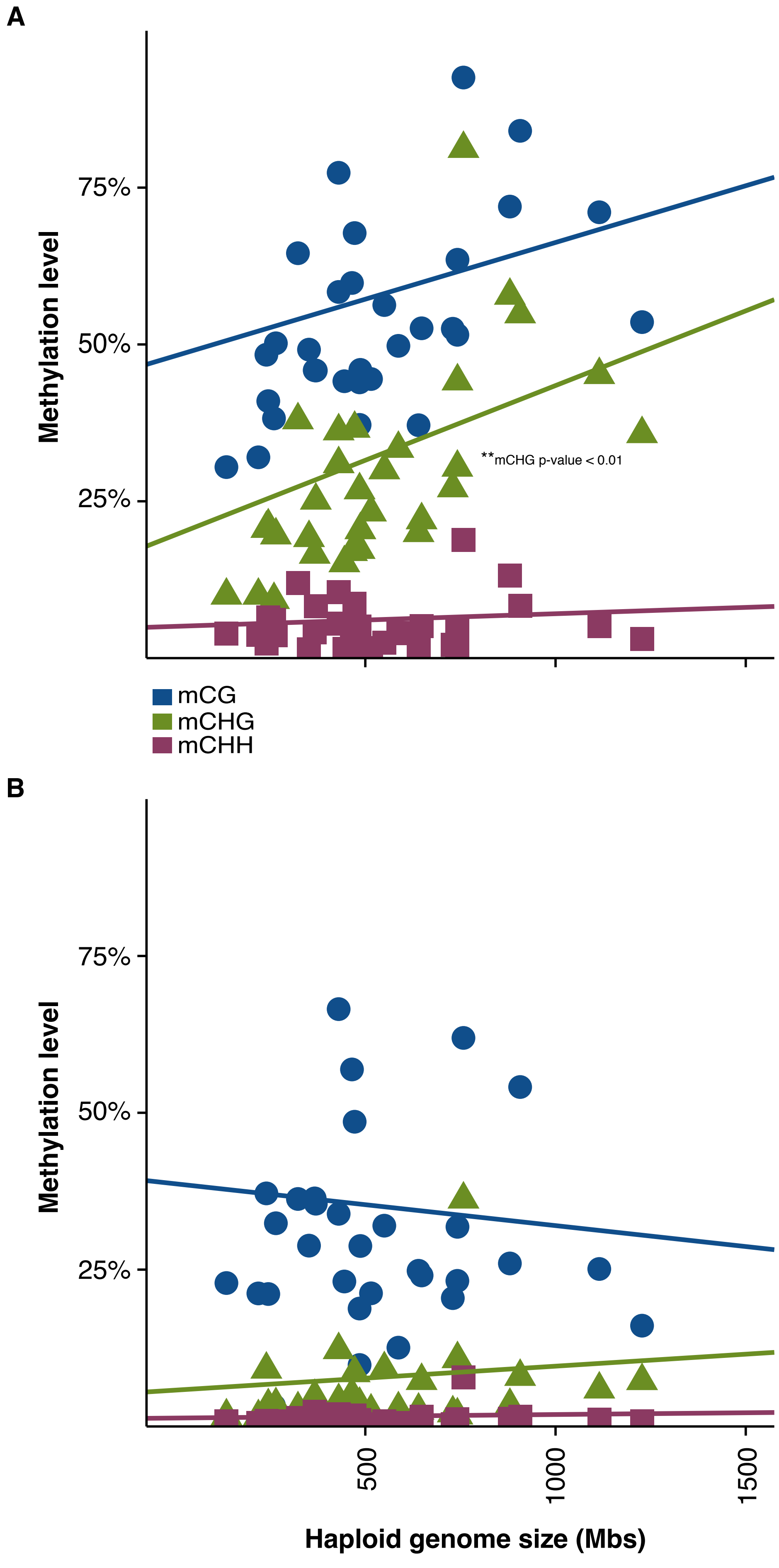

Figure S10: Methylation profile and gene distribution for chromosome 1, scaffold 1 or largest scaffold for each species. For scaffolds <10Mbs,window size is 10kbs, all others are 100 kbs windows.

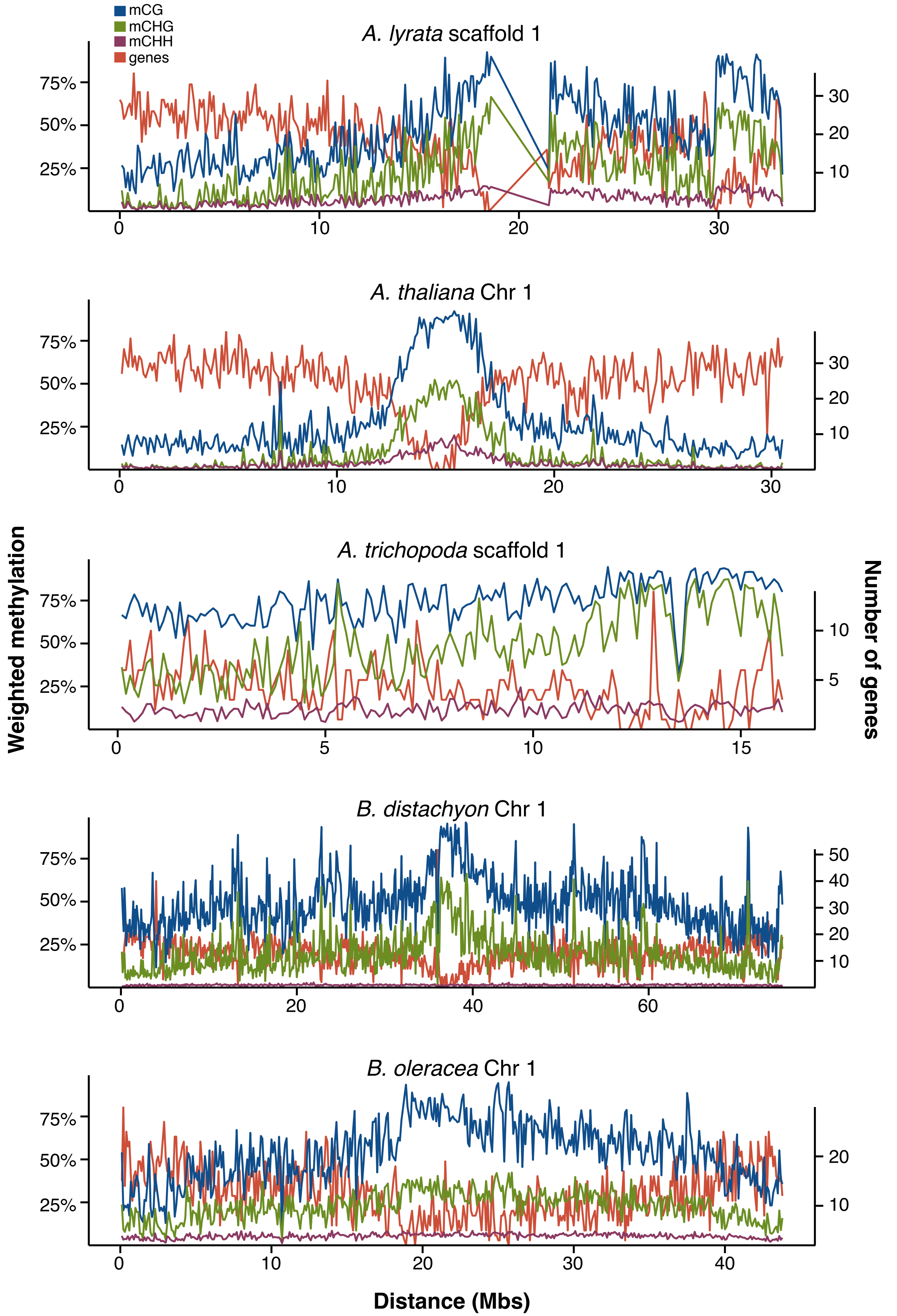

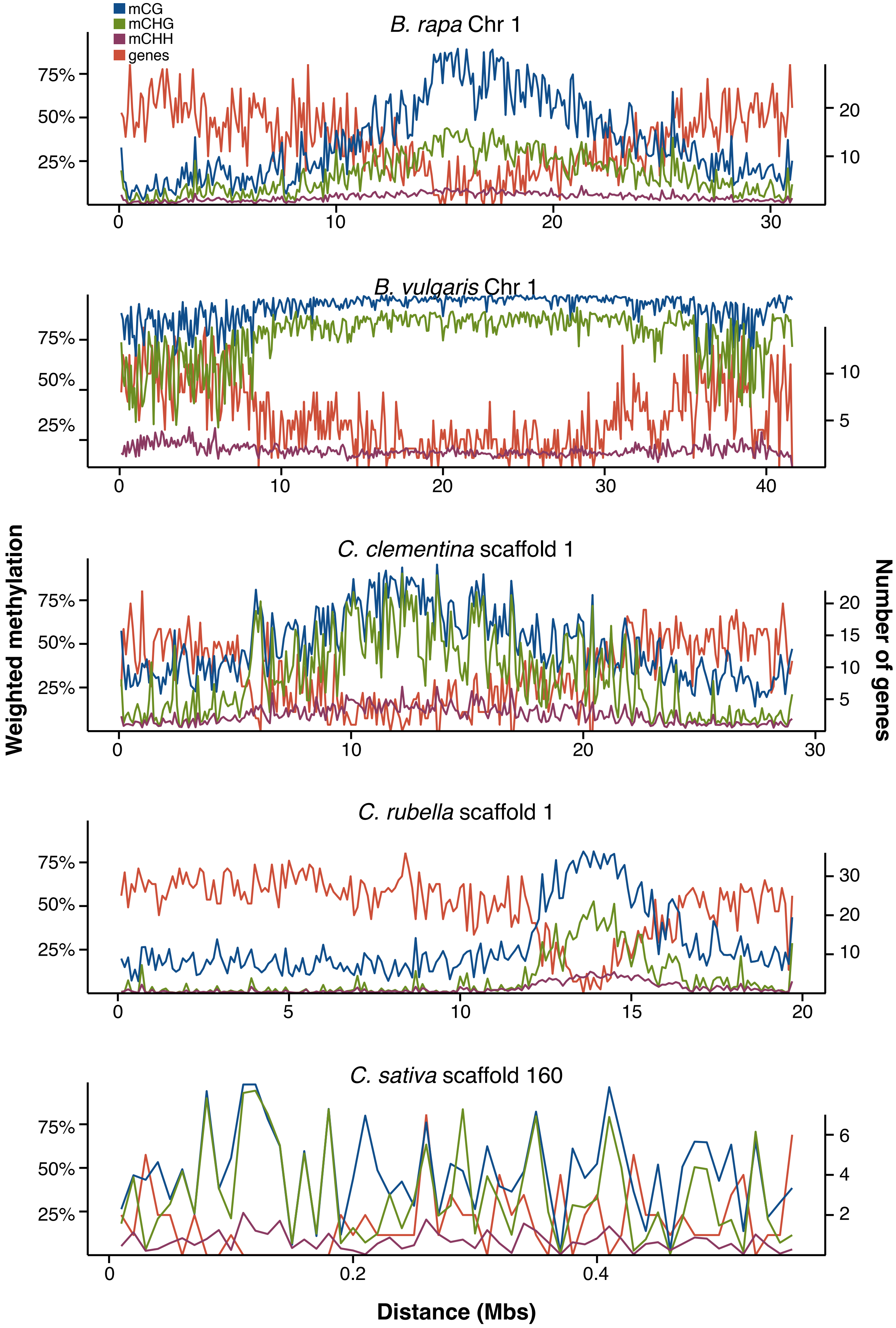

mCG
mCHG
mCHH
genes
B. rapa Chr 1
B. vulgaris Chr 1
C. clementina scaffold 1
C. rubella scaffold 1
C. sativa scaffold 160
Weighted methylation
Number of genes
Distance (Mbs)

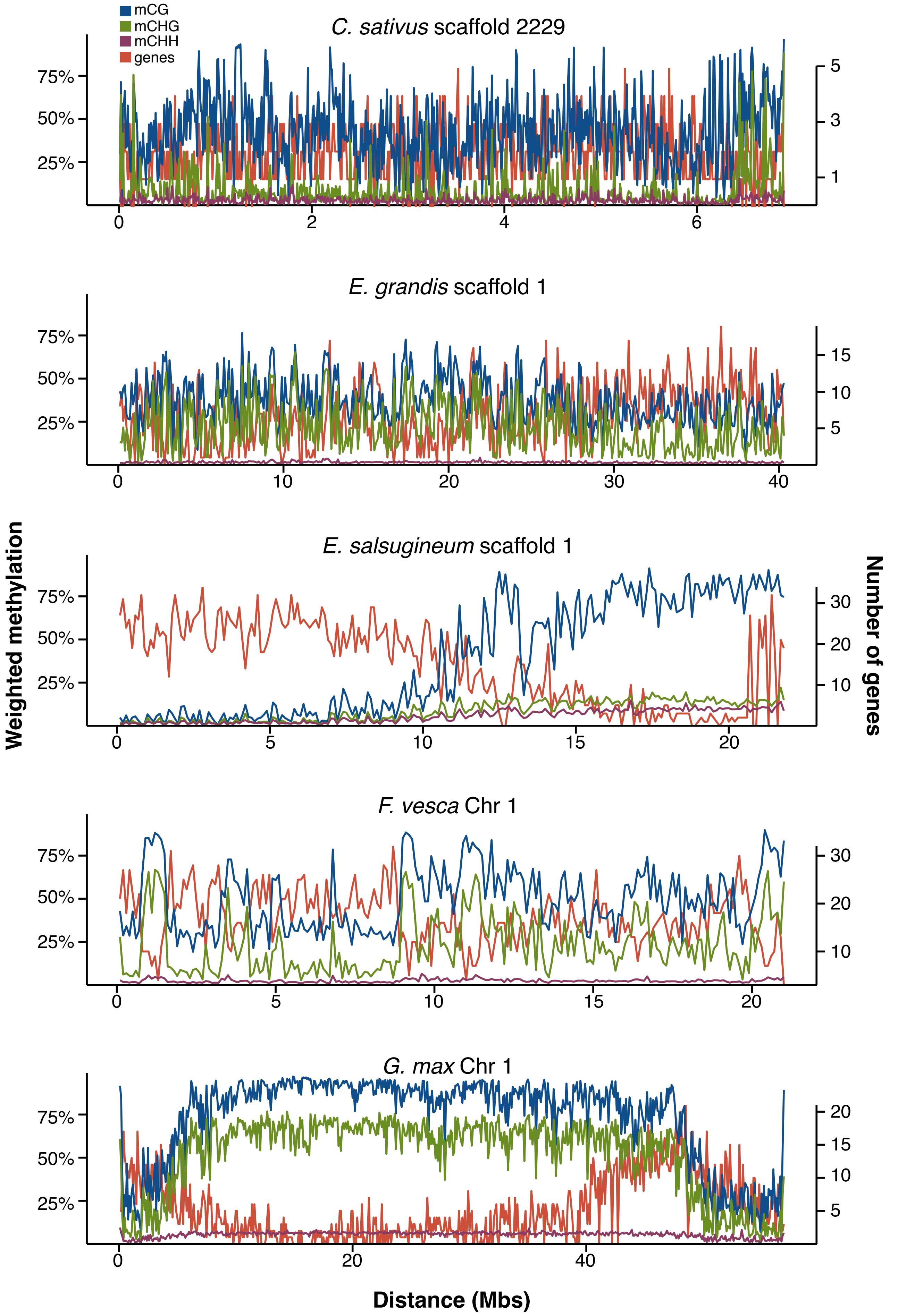
mCG
mCHG
mCHH
genes
C. sativus scaffold 2229
E. grandis scaffold 1
E. salsugineum scaffold 1
F. vesca Chr 1
G. max Chr 1
Weighted methylation
Number of genes
Distance (Mbs)
75%
50%
25%

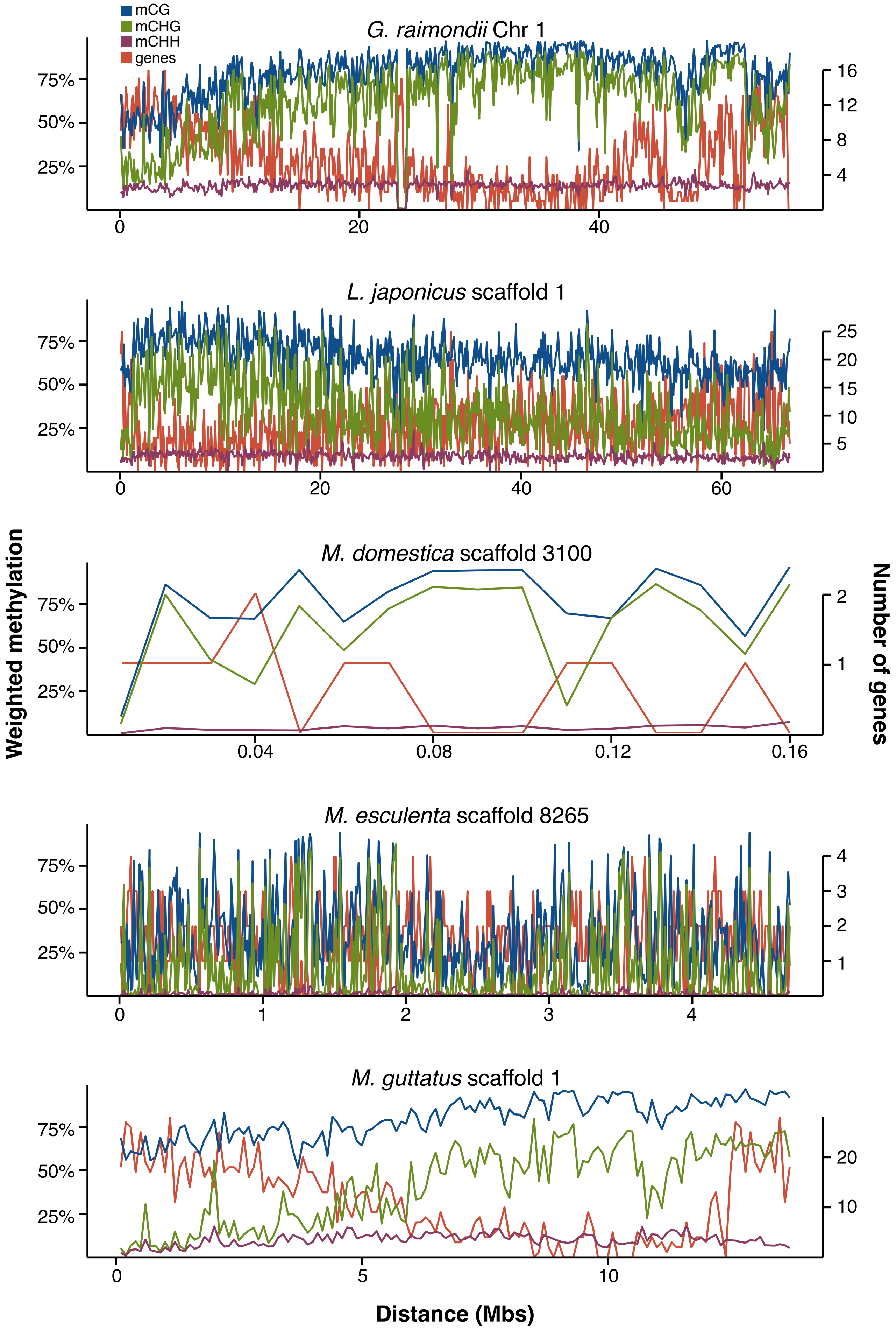
mCG
mCHG
mCHH
genes
G. raimondii Chr 1
L. japonicus scaffold 1
M. domestica scaffold 3100
M. esculenta scaffold 8265
M. guttatus scaffold 1
Weighted methylation
Number of genes
Distance (Mbs)

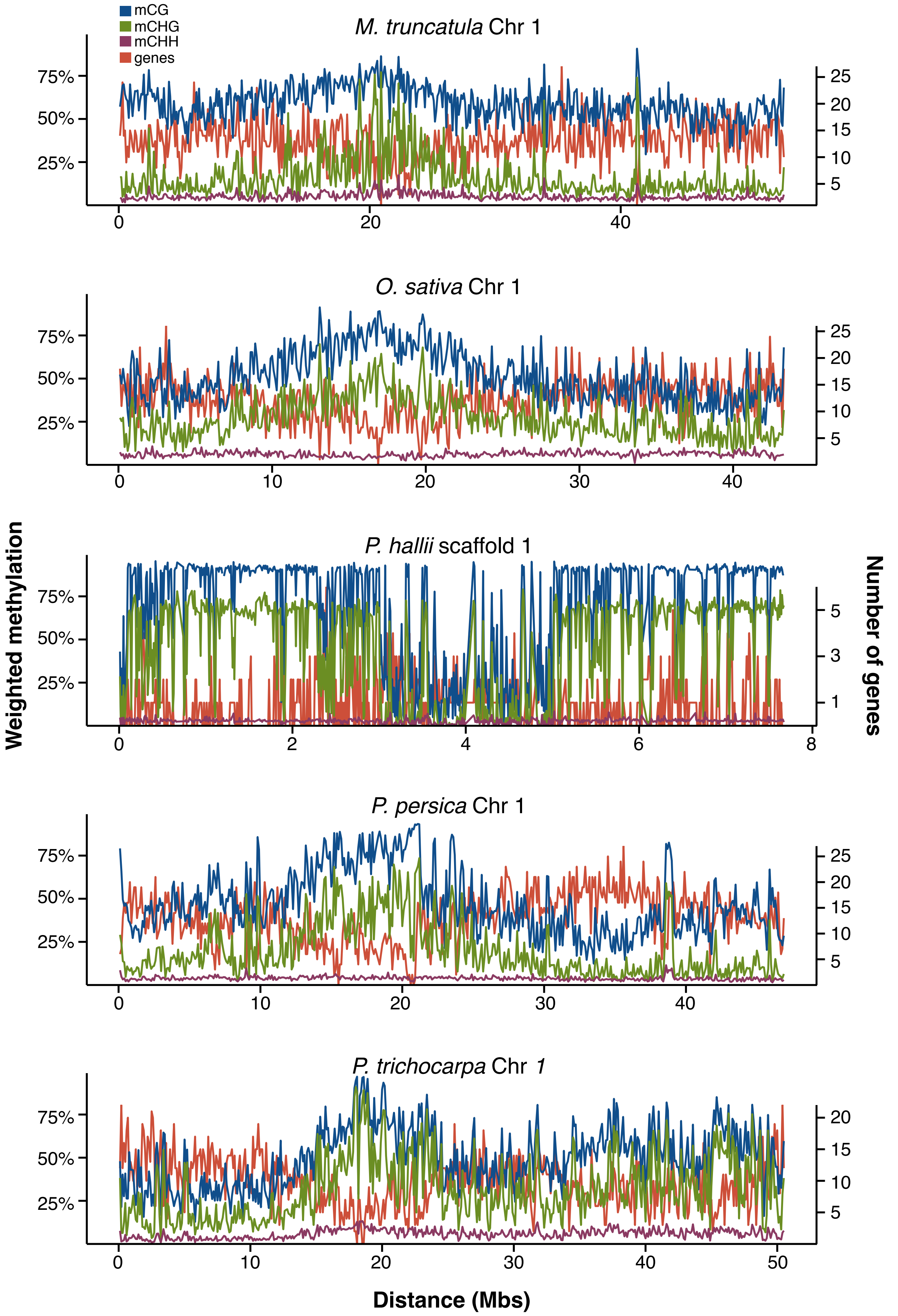
mCG
mCHG
mCHH
genes
M. truncatula Chr 1
O. sativa Chr 1
P. hallii scaffold 1
P. persica Chr 1
P. trichocarpa Chr 1
Weighted methylation
Number of genes
Distance (Mbs)

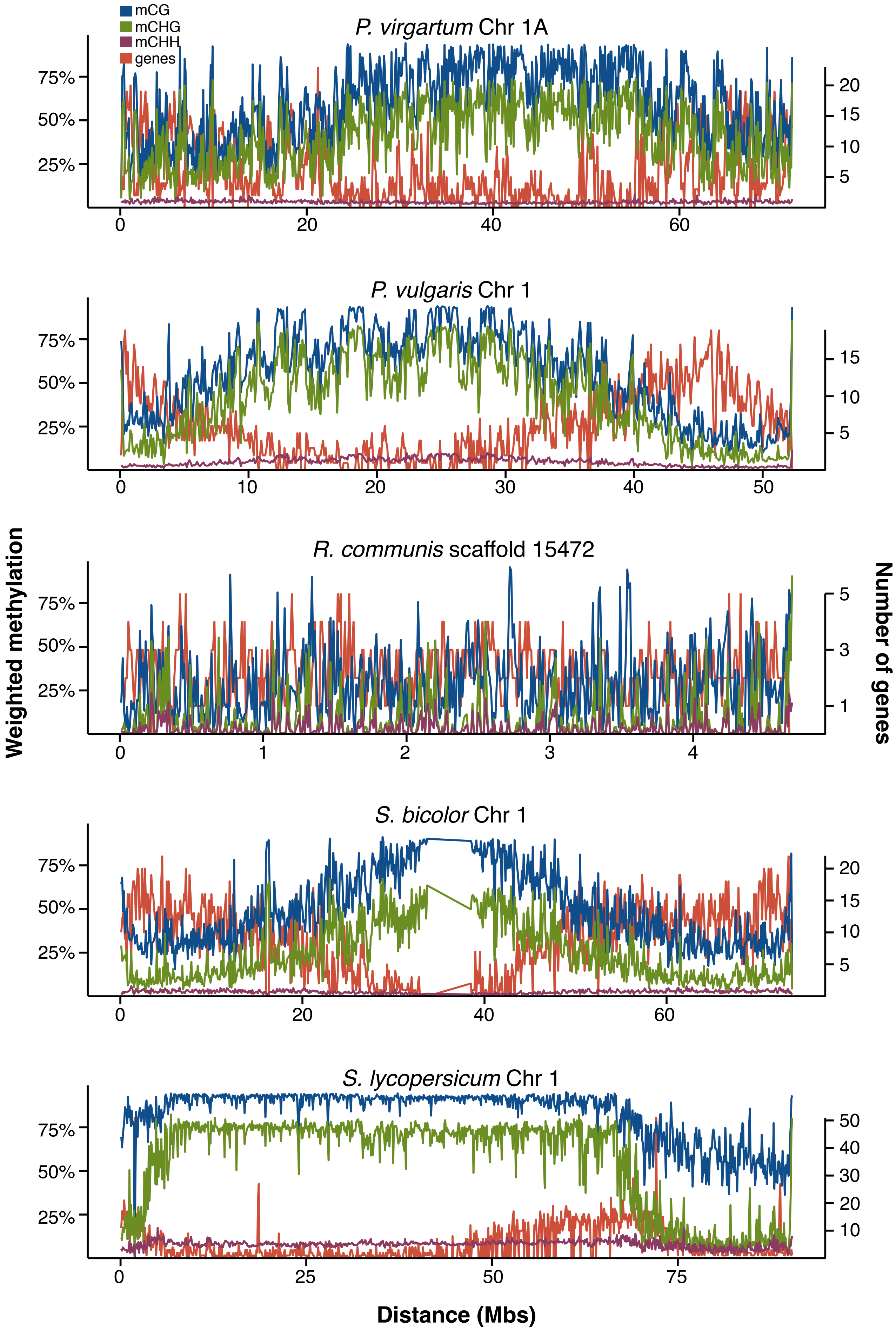

mCG
mCHG
mCHH
genes
P. virgartum Chr 1A
P. vulgaris Chr 1
R. communis scaffold 15472
S. bicolor Chr 1
S. lycopersicum Chr 1
Weighted methylation
Number of genes
Distance (Mbs)

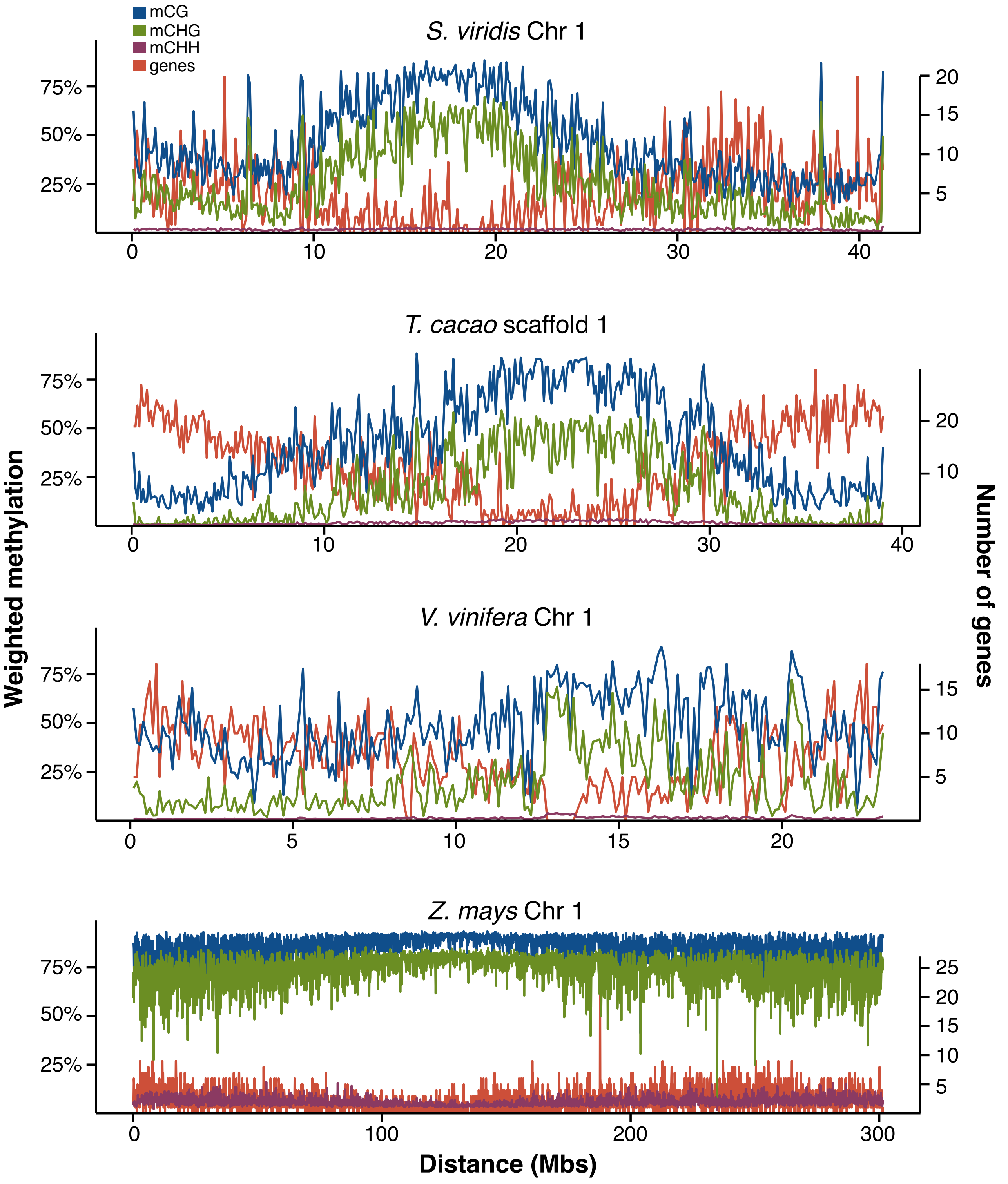
mCG
mCHG
mCHH
genes
S. viridis Chr 1
T. cacao scaffold 1
V. vinifera Chr 1
Z. mays Chr 1
Weighted methylation
Number of genes
Distance (Mbs)
75%
50%
25%
0
10
20
30
40
5
15
100
200
300
