# Supplementary material for: Widespread natural variation of DNA methylation within angiosperms
Source: Genome Biol. 2016 Sep 27;17:194. doi: 10.1186/s13059-016-1059-0 (PMC5037628; doi:10.1186/s13059-016-1059-0)

Figure S11: Methylation level and length in base pairs for **(A)** mCG regions, **(B)** mCHG regions, **(C)** and mCHH regions.

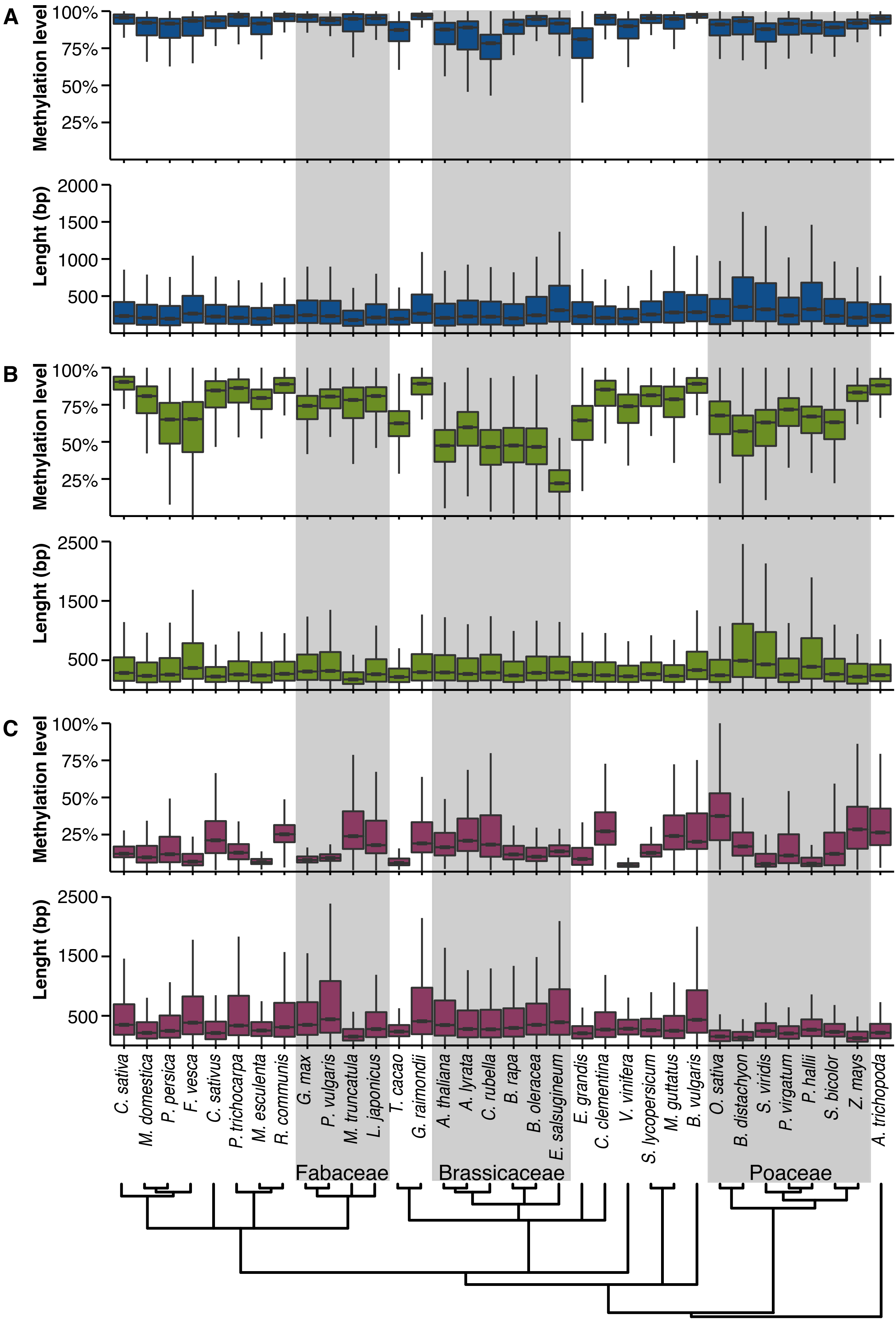

Figure S12: Size in base pairs of mCG, mCHG, and mCHH regions for each species based on the methylation levels of those regions: < 5%, 5-15%, 15-25%, > 25%.

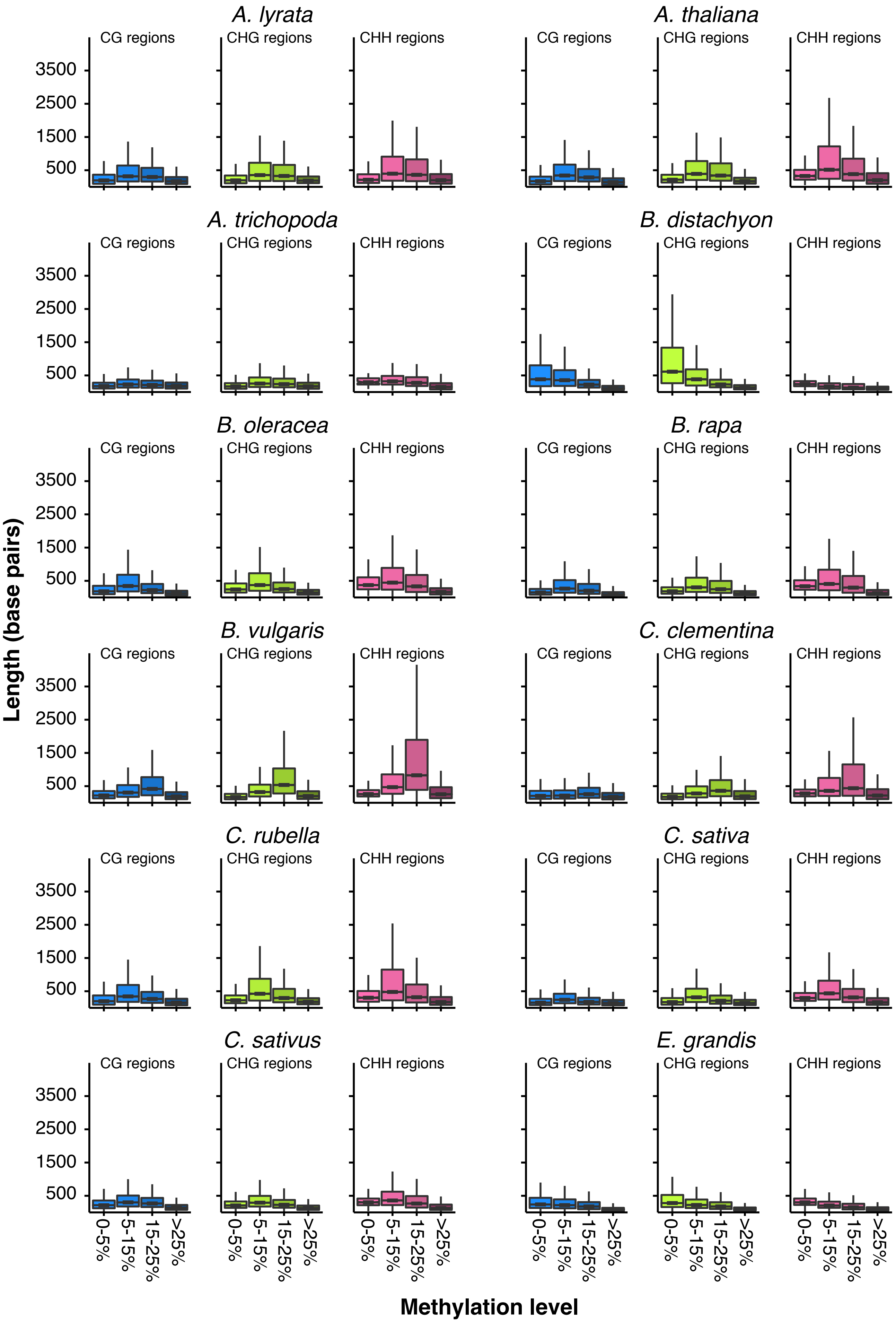

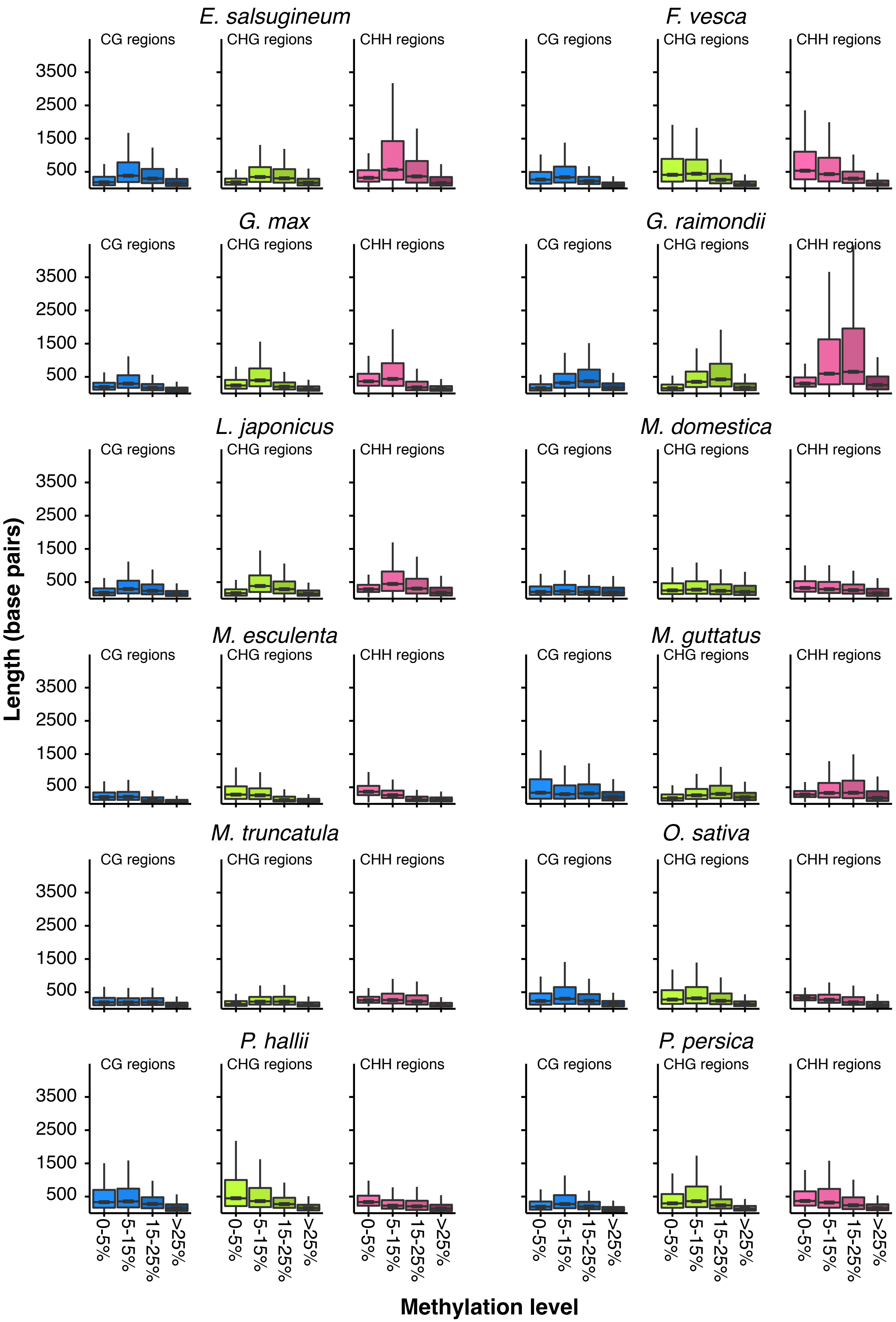
E. salsugineum
F. vesca
G. max
G. raimondii
L. japonicus
M. domestica
M. esculenta
M. guttatus
M. truncatula
O. sativa
P. hallii
P. persica
CG regions
CHG regions
CHH regions
3500
2500
1500
500
0-5%
5-15%
15-25%
>25%
Length (base pairs)
Methylation level

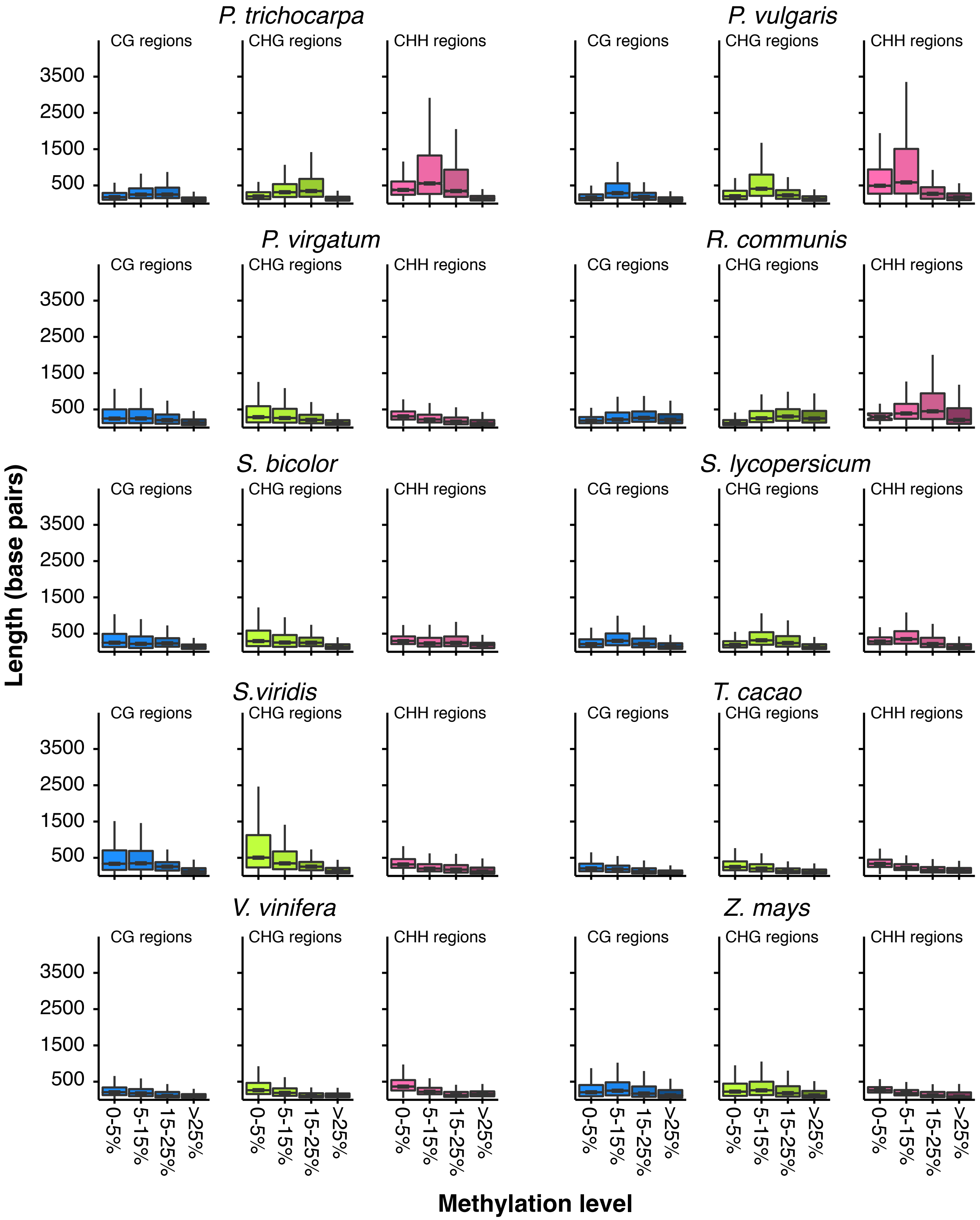
P. trichocarpa
P. vulgaris
P. virgatum
R. communis
S. bicolor
S. lycopersicum
S.viridis
T. cacao
V. vinifera
Z. mays
CG regions
CHG regions
CHH regions
3500
2500
1500
500
Length (base pairs)
0-5%
5-15%
15-25%
>25%
Methylation level

Figure S13: sRNA levels for each sRNA size class in mCHH regions for each species with sRNA data. mCHH regions are broken into four groups based on methylation level of that region: < 5% mCHH, 5-15% mCHH, 15-25% mCHH, > 25% mCHH.

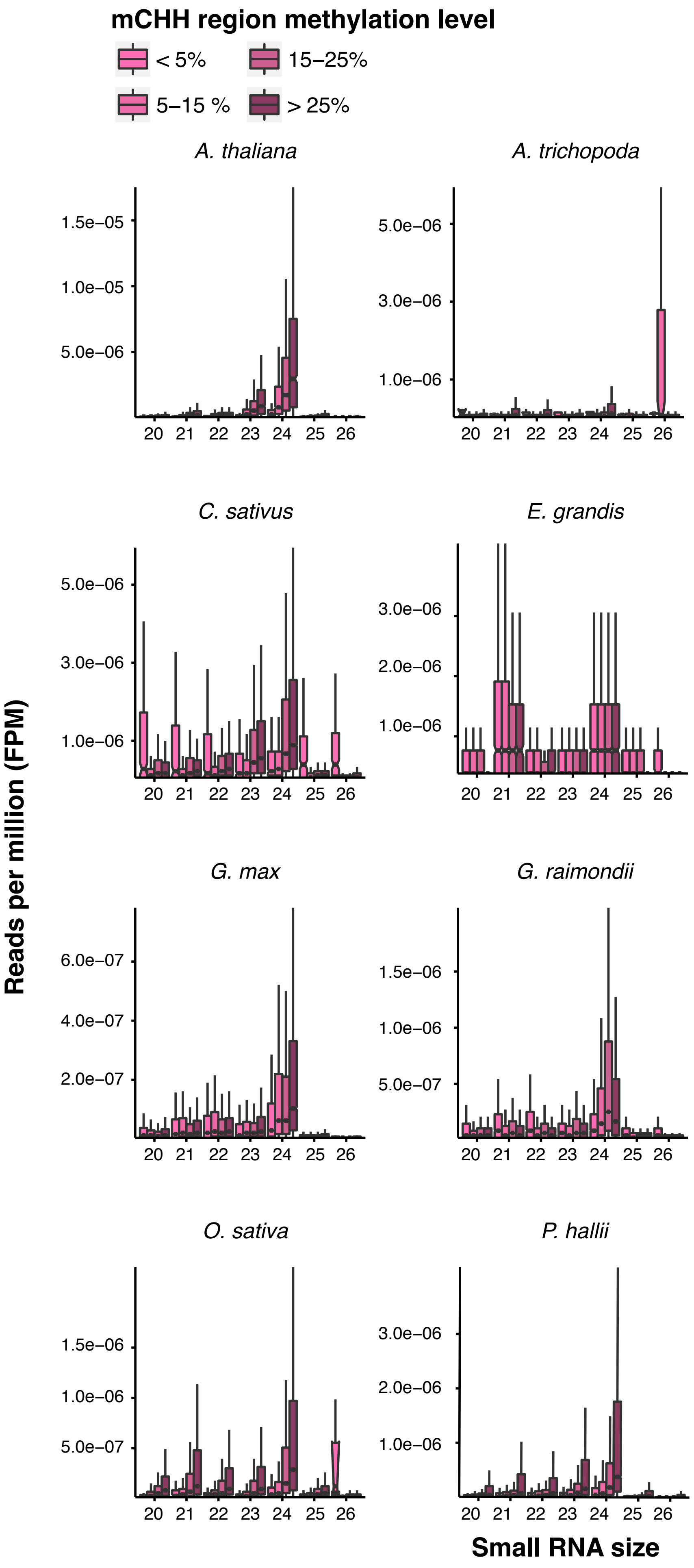

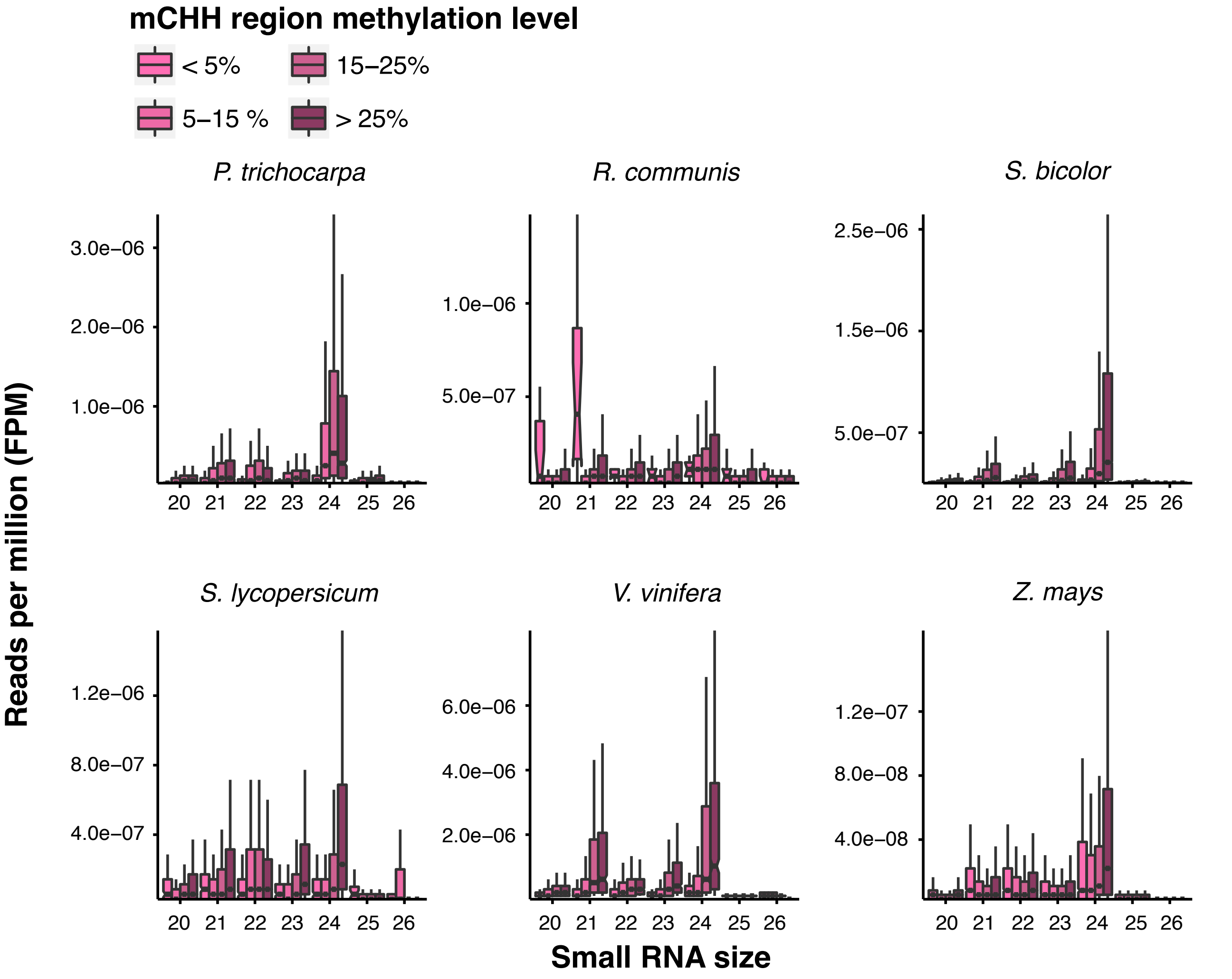
mCHH region methylation level
< 5%
15–25%
5–15 %
> 25%
P. trichocarpa
R. communis
S. bicolor
S. lycopersicum
V. vinifera
Z. mays
Reads per million (FPM)
Small RNA size
20
21
22
23
24
25
26
3.0e−06
2.0e−06
1.0e−06
5.0e−07
2.5e−06
1.5e−06
1.2e−06
8.0e−07
4.0e−07
6.0e−06
4.0e−06
2.0e−06
1.2e−07
8.0e−08
4.0e−08

Figure S14: mCHH regions were assigned to quartiles based on 24nt siRNA levels and the distribution of their lengths plotted. 1st quartile = lowest 24nt siRNA level, 4th quartile = highest 24nt siRNA levels.

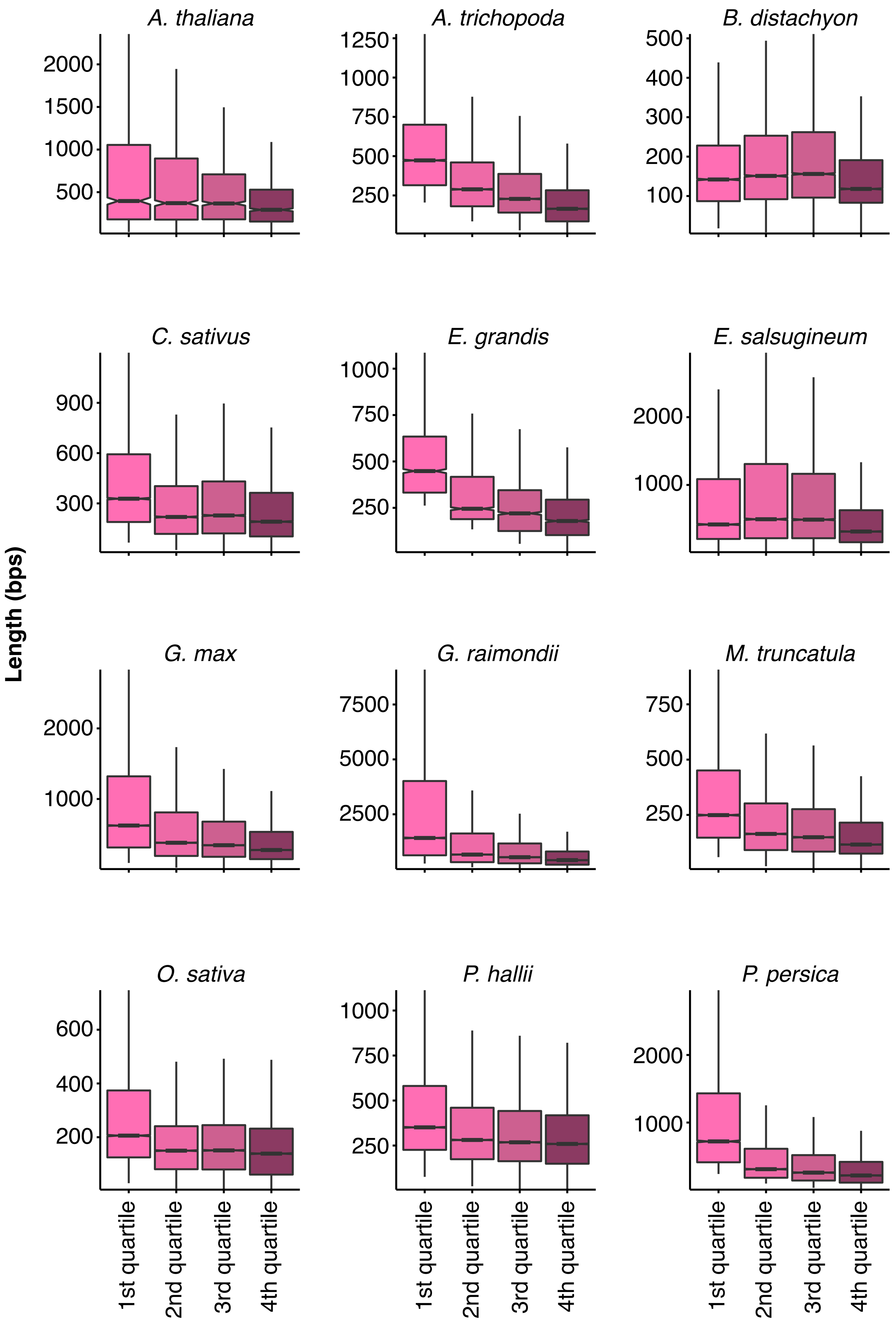

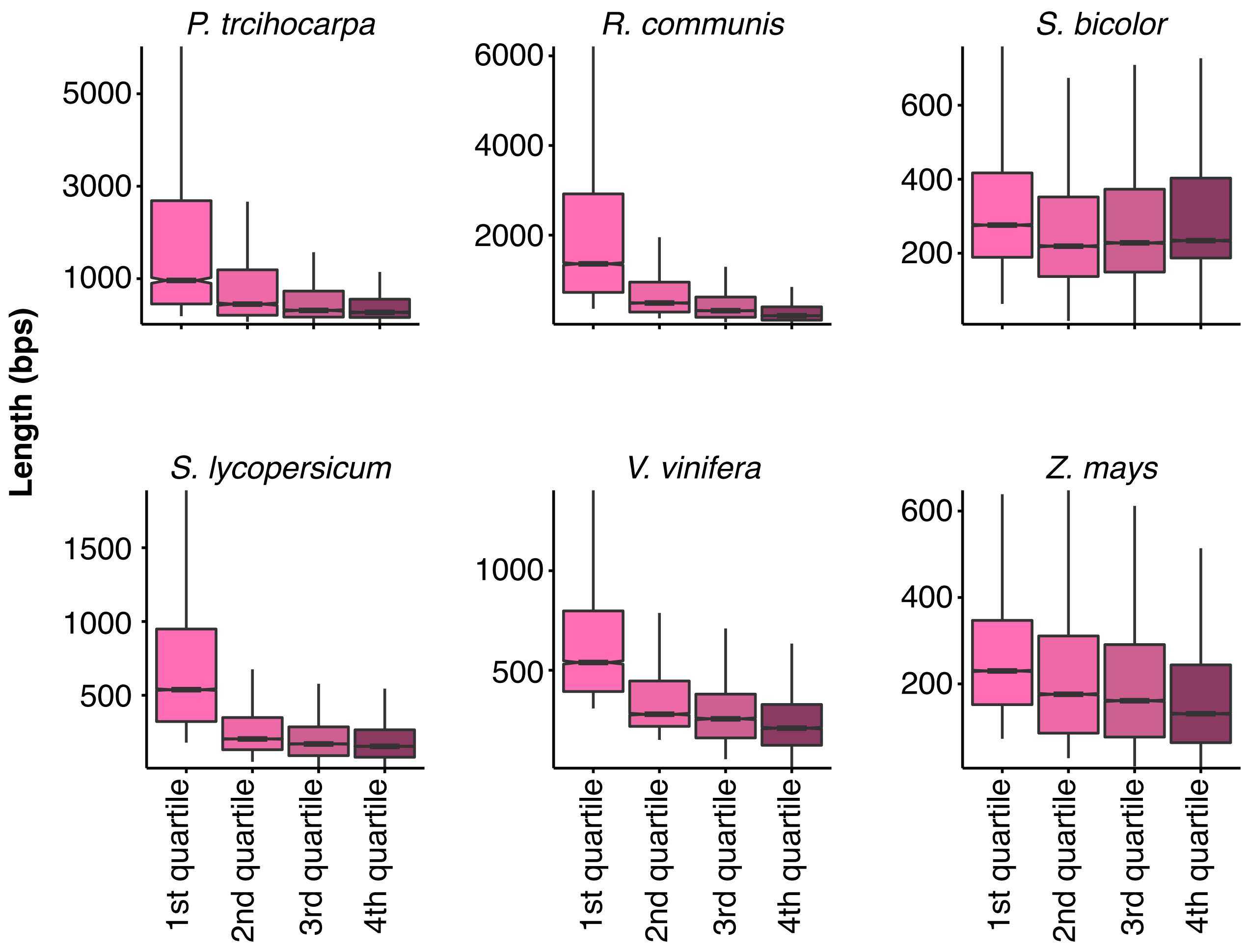

P. trcihocarpa
R. communis
S. bicolor
S. lycopersicum
V. vinifera
Z. mays
Length (bps)
5000
3000
1000
6000
4000
2000
600
400
200
1500
1000
500
1000
500
600
400
200
1st quartile
2nd quartile
3rd quartile
4th quartile

Figure S15: (A) mCG (blue), mCHG (green), mCHH (maroon) levels within CDS plotted against total number of repeats in the genome. **(B)** mCG (blue), mCHG (green), mCHH (maroon) levels within coding sequences (CDS) plotted against the percentage of genes with repeats within genes (UTR, CDS, and intron). **(C)** Percentage of genes with repeats plotted against total number of repeats in the genome.

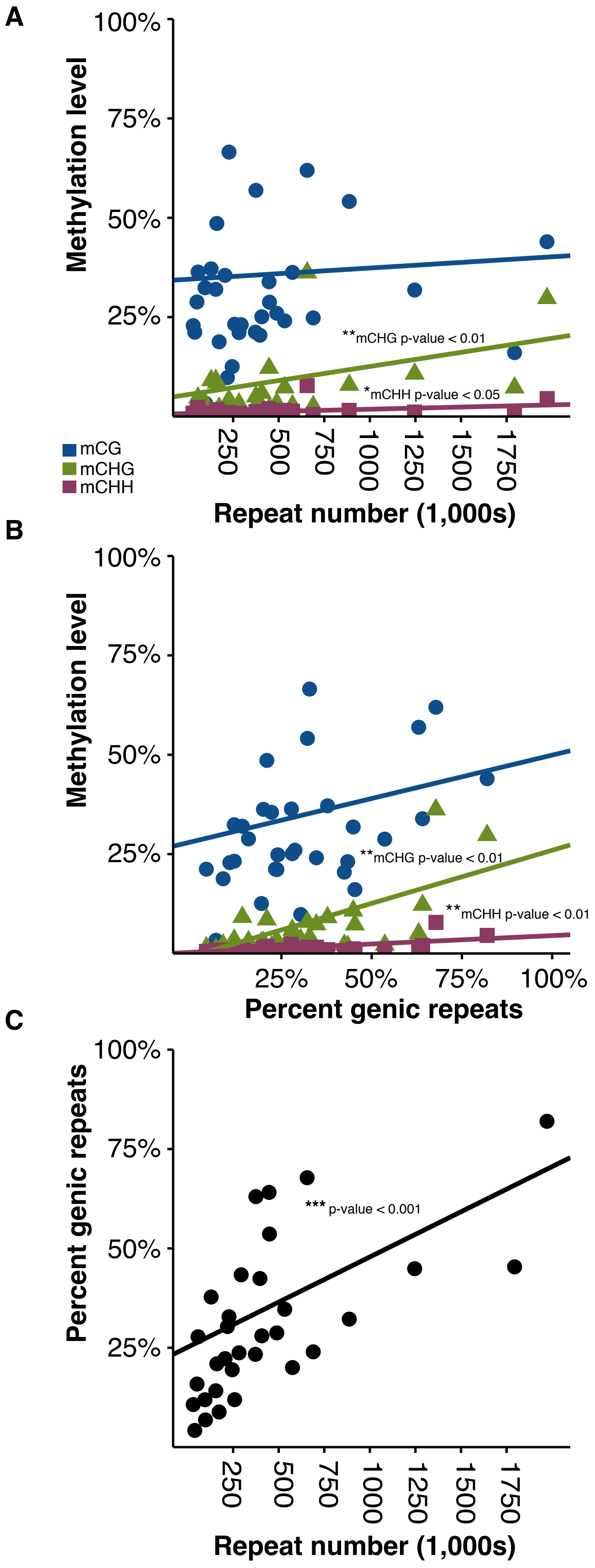

Figure S16: Methylation levels for mCG (blue), mCHG (green), and mCHH (maroon) upstream, across, and downstream of repeat sequences were plotted for all species with annotated repeats.

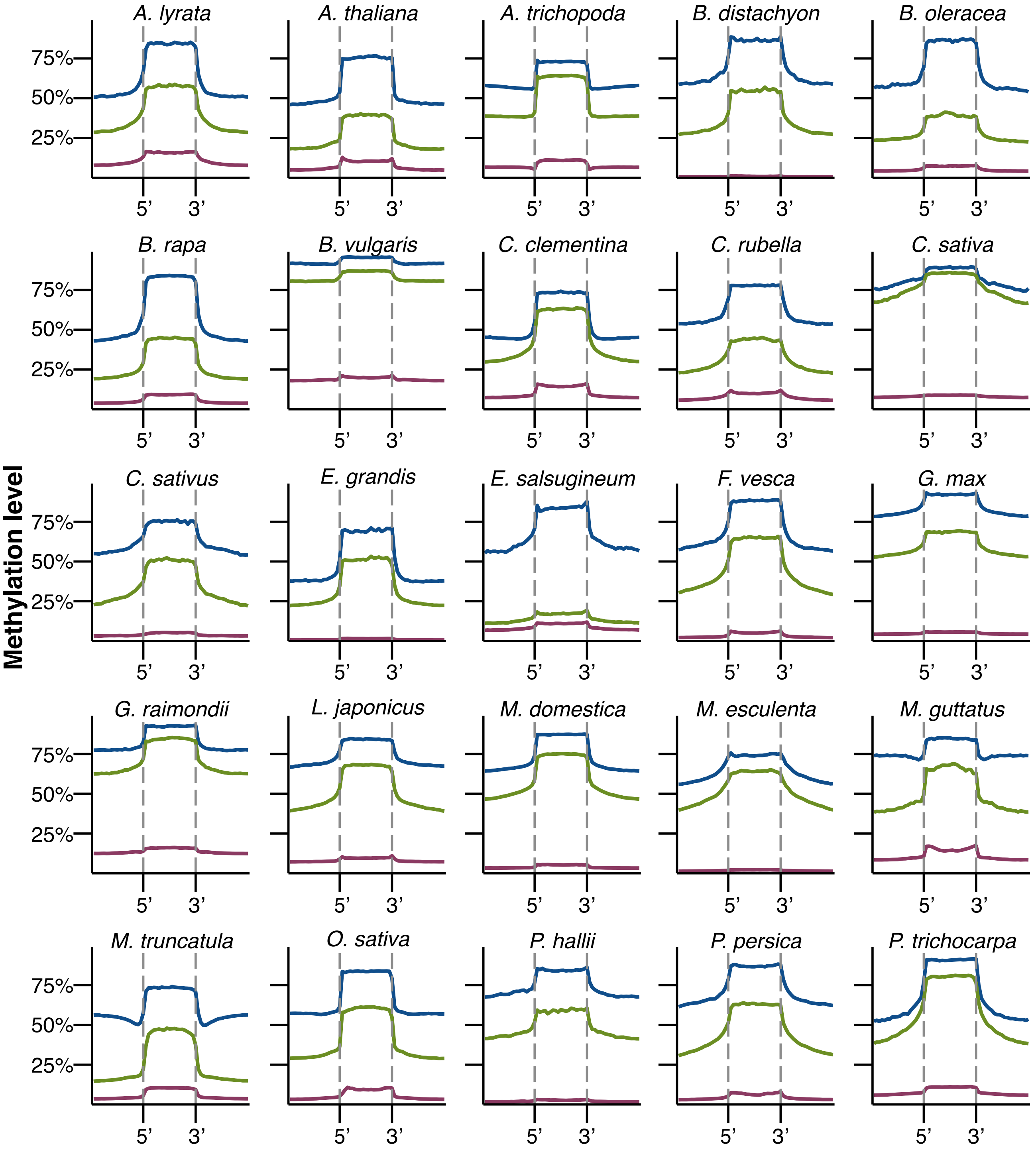

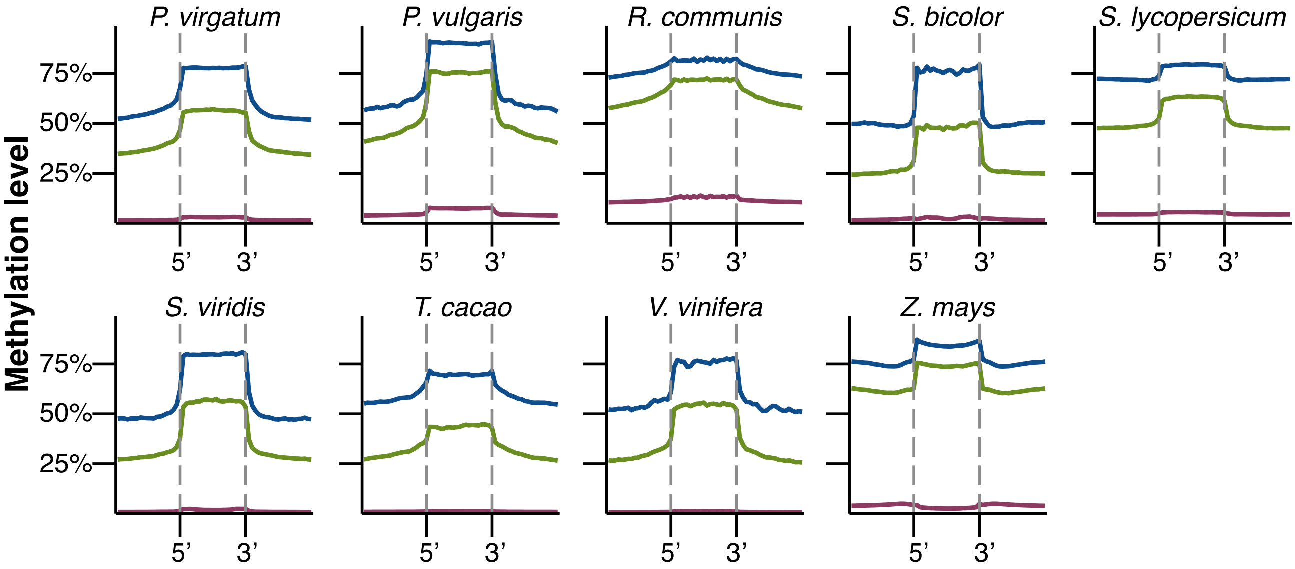

Methylation level
P. virgatum
P. vulgaris
R. communis
S. bicolor
S. lycopersicum
S. viridis
T. cacao
V. vinifera
Z. mays
75%
50%
25%
5'
3'

Figure S17: A binomial test was used to determine if a statistically significant number of methylated site within coding regions for each sequence context. Depending upon which contexts were methylated, genes were classified into one of four methylation classes. Browser screen shots of example genes from *F. vesca* are shown.

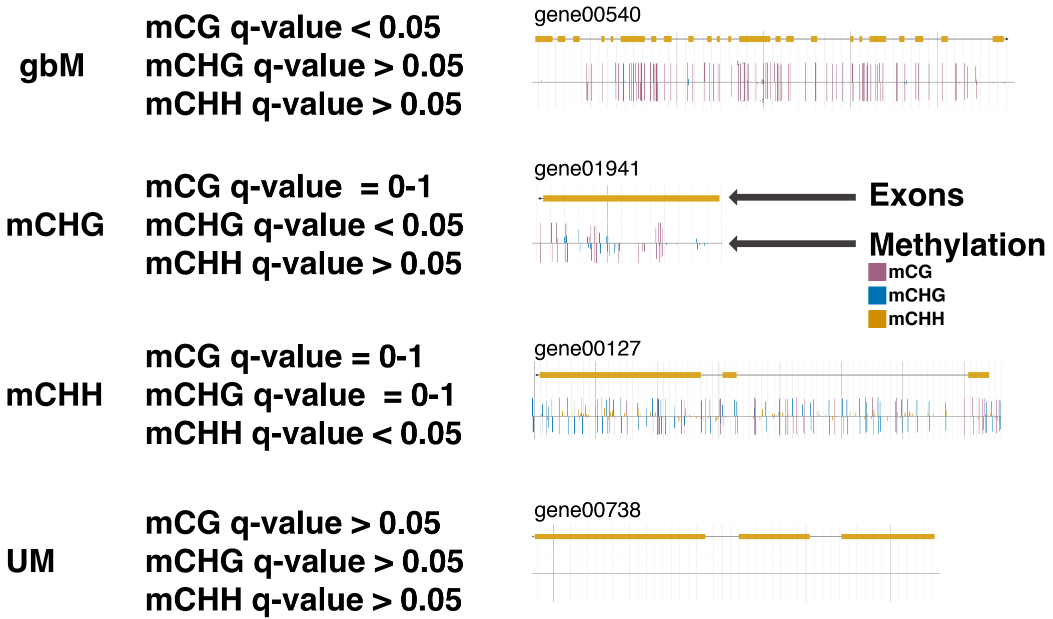

Figure S18: Methylation levels for mCG (blue), mCHG (green), and mCHH (maroon), upstream, across, and downstream for all genes, CG gbM genes, mCHG genes, and mCHH genes for all species.

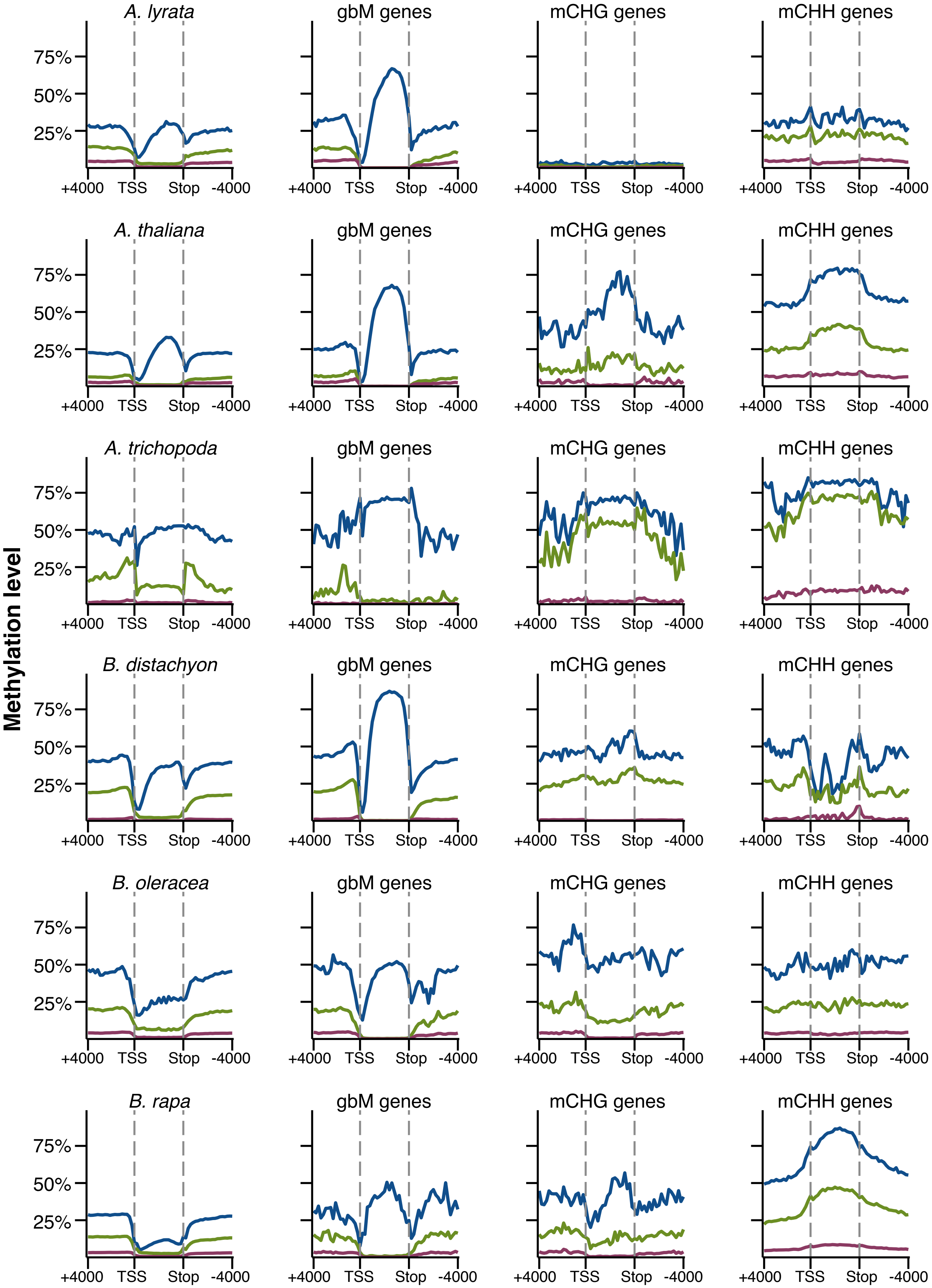

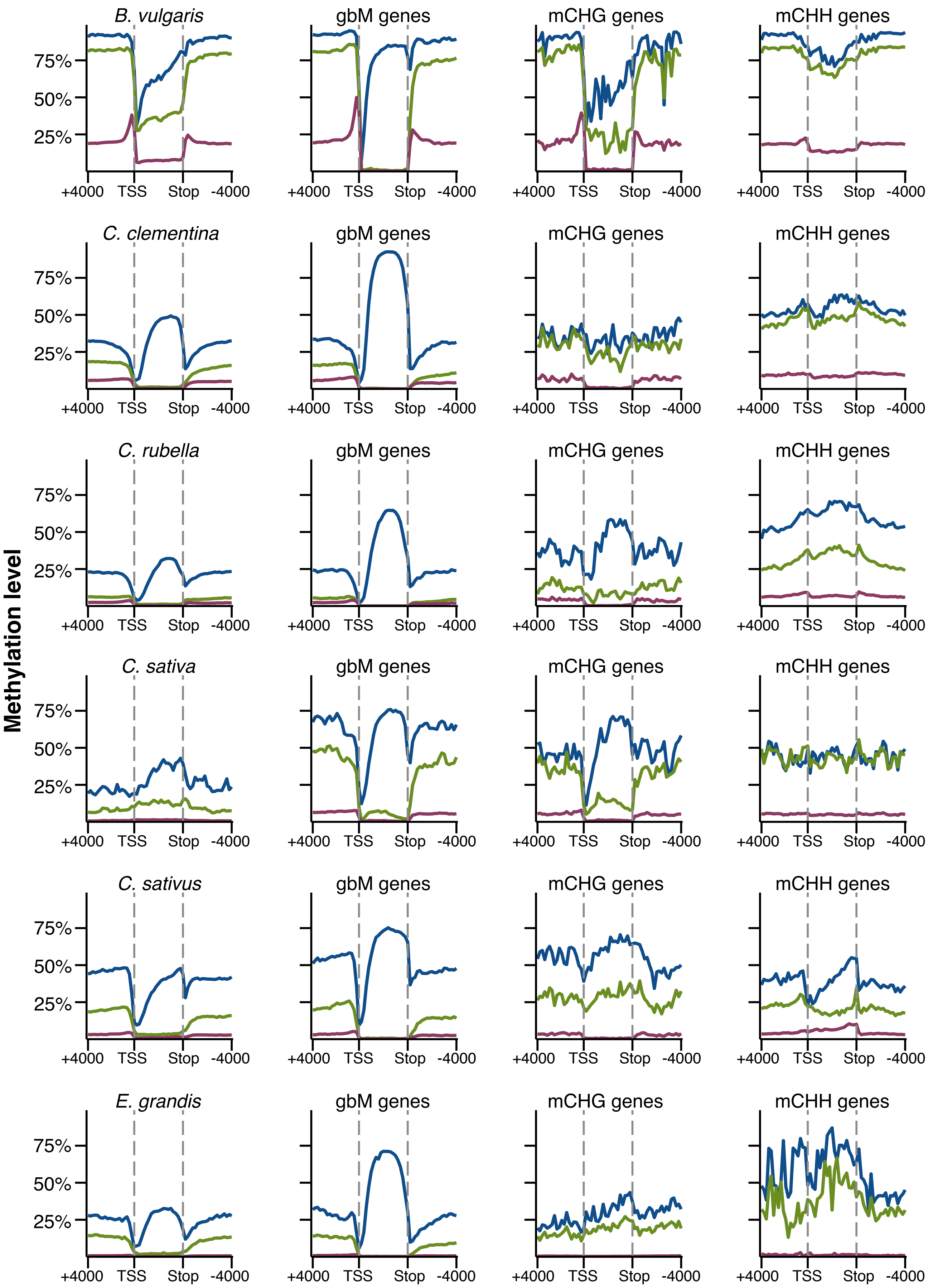
Methylation level
B. vulgaris
C. clementina
C. rubella
C. sativa
C. sativus
E. grandis
gbM genes
mCHG genes
mCHH genes
75%
50%
25%
+4000
TSS
Stop
-4000

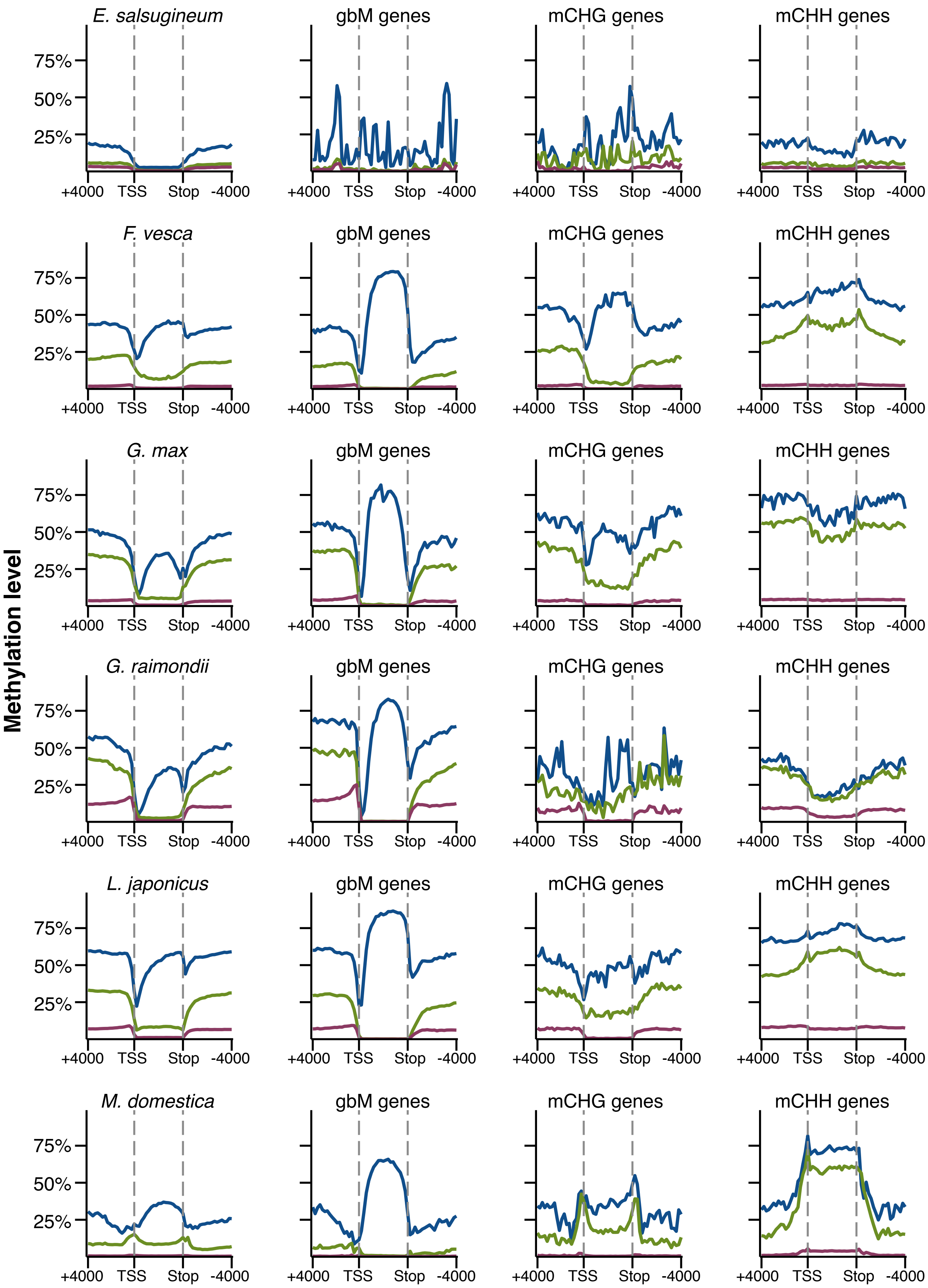
E. salsugineum
F. vesca
G. max
G. raimondii
L. japonicus
M. domestica
gbM genes
mCHG genes
mCHH genes
Methylation level
75%
50%
25%
+4000
TSS
Stop
-4000

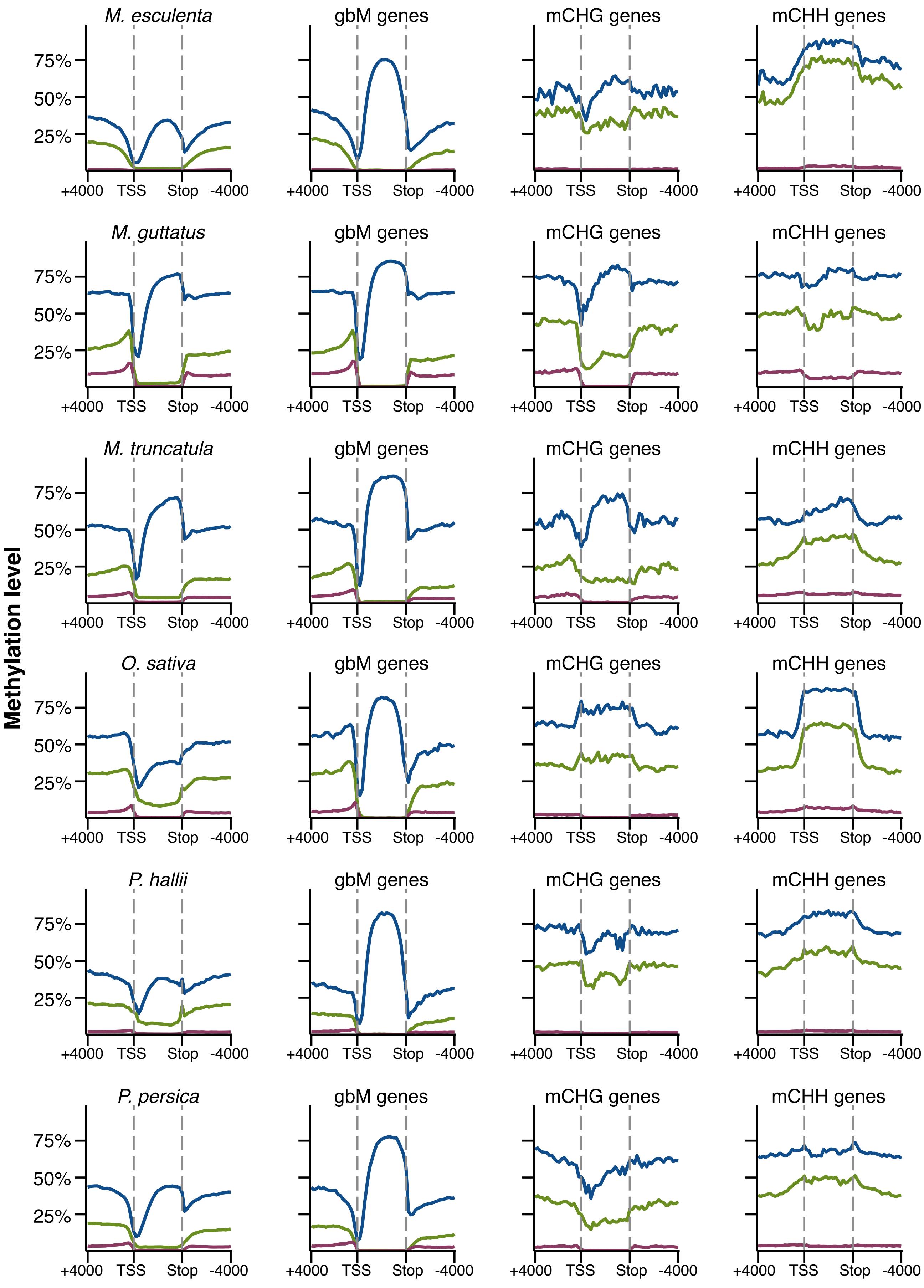

Methylation level
M. esculenta
M. guttatus
M. truncatula
O. sativa
P. hallii
P. persica
gbM genes
mCHG genes
mCHH genes
75%
50%
25%
+4000
TSS
Stop
-4000

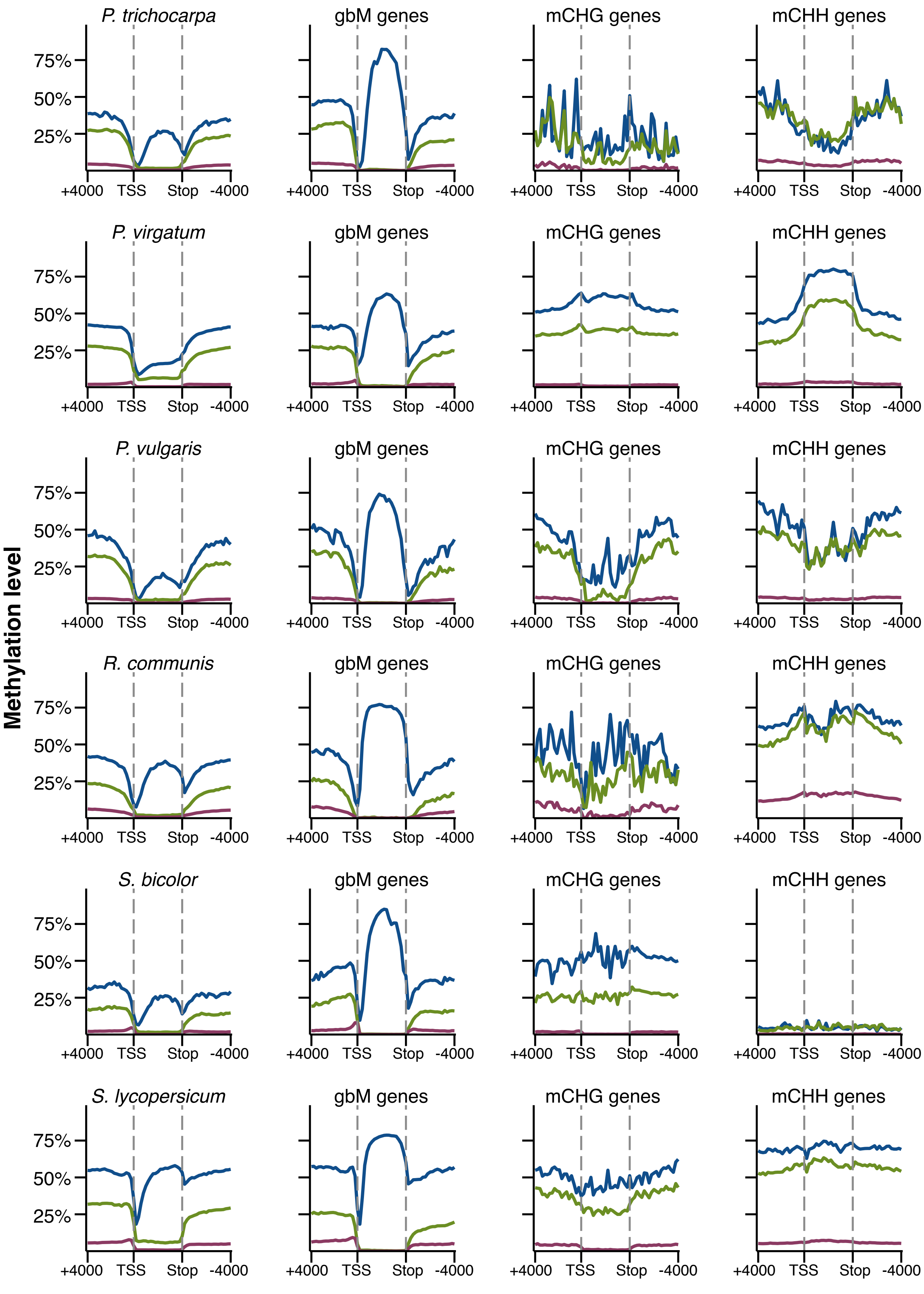

P. trichocarpa
P. virgatum
P. vulgaris
R. communis
S. bicolor
S. lycopersicum
gbM genes
mCHG genes
mCHH genes
Methylation level
75%
50%
25%
+4000
TSS
Stop
-4000

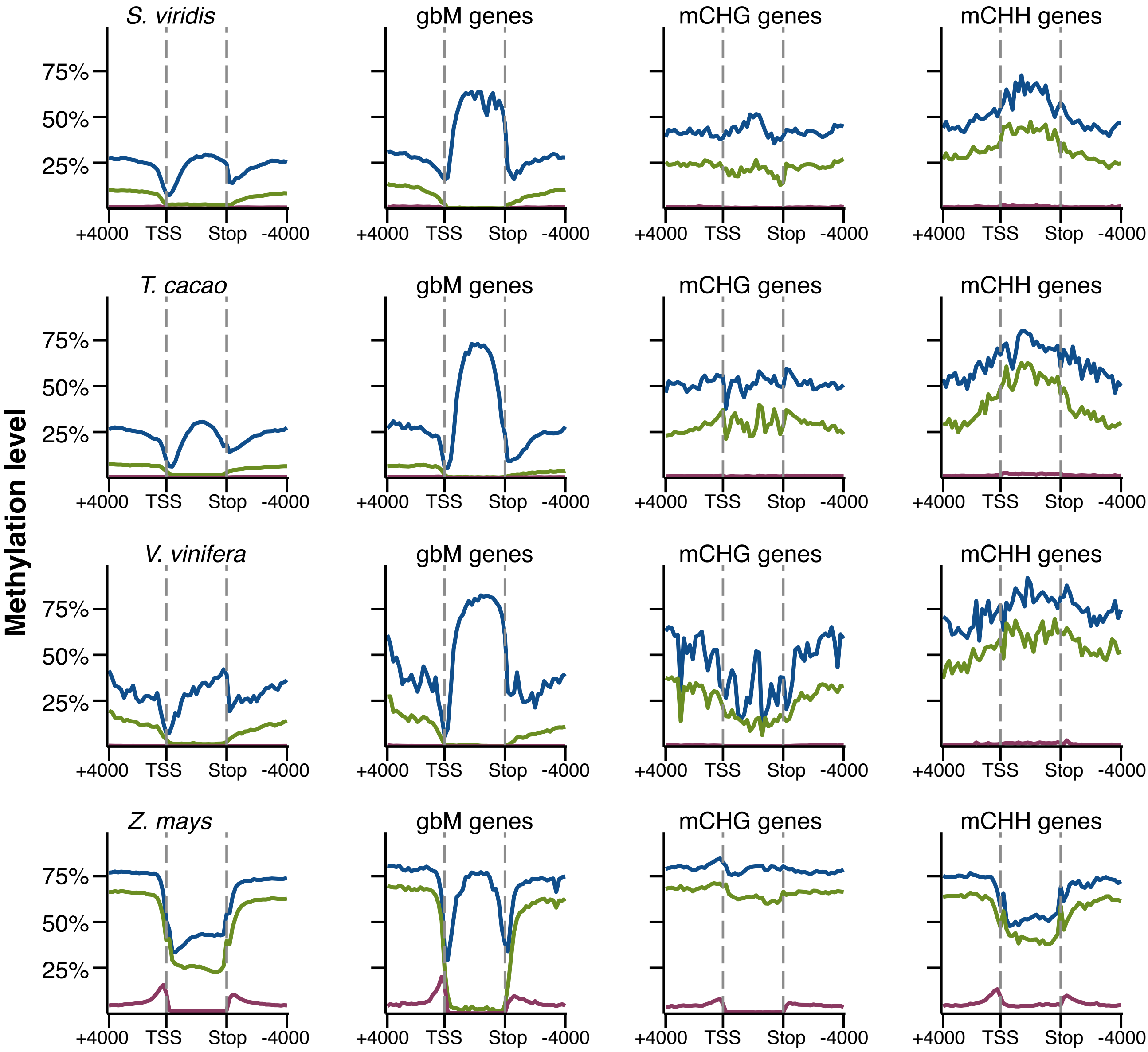
S. viridis
T. cacao
V. vinifera
Z. mays
gbM genes
mCHG genes
mCHH genes
Methylation level
75%
50%
25%
+4000
TSS
Stop
-4000

Figure S19: Gene expression levels (FPKM) for all genes, CG gbM genes, mCHG genes, mCHH, and mCG-TSS genes for all species with RNA-seq data.

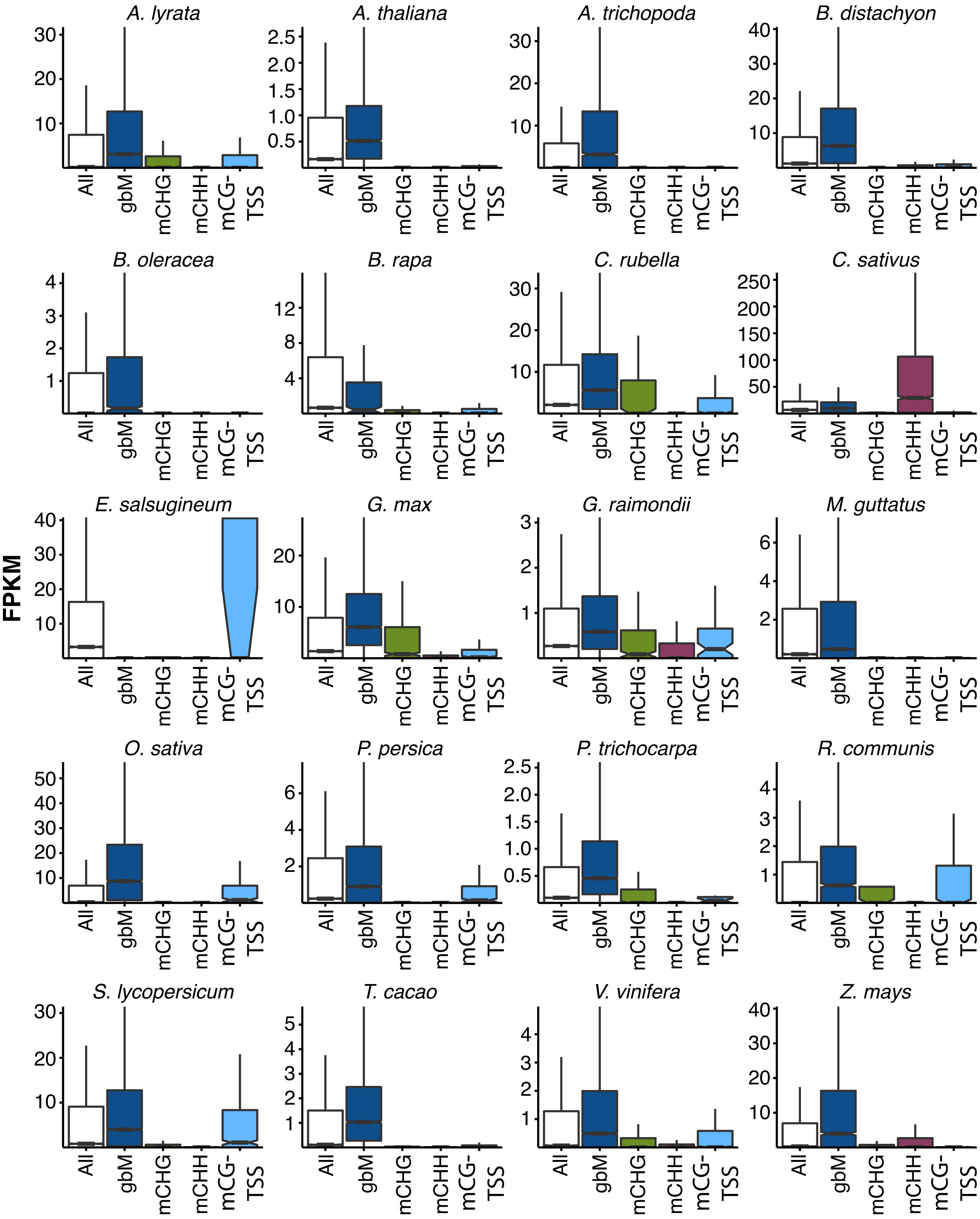

Table S5: Physical characteristics of CG gbM genes versus UM genes: gene length, exon number, and [O/E] observed number of CG dinucleotides versus expected number given GC content of the gene. The molecular evolution of CG gbM genes versus UM genes was determined using either *A. thaliana* or *O. sativa* as an outgroup. Molecular evolution characteristics showing non-synonymous mutations (dN), synonymous mutations (dS) and and the ratio of dN/dS (ω). Those in red do not conform to prediction.

	Physical characteristics			Molecular evolution characteristics			
	Length	Exon #	[O/E]	Outgroup	*dN*	*dS*	ω
C. sativa	gbM>UM	gbM>UM	gbM~UM	*A. thaliana*	gbM~UM	gbM~UM	gbM~UM
M. domestica	gbM>UM	gbM>UM	gbM>UM	*A. thaliana*	gbM~UM	gbM<UM	gbM~UM
P. persica	gbM>UM	gbM>UM	gbM<UM	*A. thaliana*	gbM<UM	gbM<UM	gbM~UM
F. vesca	gbM>UM	gbM>UM	gbM<UM	*A. thaliana*	gbM~UM	gbM<UM	gbM~UM
C. sativus	gbM>UM	gbM>UM	gbM<UM	*A. thaliana*	gbM<UM	gbM<UM	gbM~UM
P. trichocarpa	gbM>UM	gbM>UM	gbM<UM	*A. thaliana*	gbM<UM	gbM<UM	gbM~UM
M. esculenta	gbM>UM	gbM>UM	gbM<UM	*A. thaliana*	gbM<UM	gbM<UM	gbM<UM
R. communis	gbM>UM	gbM>UM	gbM>UM	*A. thaliana*	gbM~UM	gbM<UM	gbM~UM
G. max	gbM>UM	gbM>UM	gbM<UM	*A. thaliana*	gbM~UM	gbM<UM	gbM~UM
P. vulgaris	gbM>UM	gbM>UM	gbM<UM	*A. thaliana*	gbM~UM	gbM<UM	gbM~UM
M. truncatula	gbM>UM	gbM>UM	gbM>UM	*A. thaliana*	gbM~UM	gbM<UM	gbM~UM
L. japonicus	gbM>UM	gbM>UM	gbM<UM	*A. thaliana*	gbM<UM	gbM<UM	gbM~UM
T. cacao	gbM>UM	gbM>UM	gbM<UM	*A. thaliana*	gbM<UM	gbM<UM	gbM~UM
G. raimondii	gbM>UM	gbM>UM	gbM<UM	*A. thaliana*	gbM<UM	gbM<UM	gbM~UM
A. thaliana	gbM>UM	gbM>UM	gbM<UM	--------------	-------------	-------------	-------------
A. lyrata	gbM>UM	gbM>UM	gbM<UM	*A. thaliana*	gbM<UM	gbM<UM	gbM<UM
C. rubella	gbM>UM	gbM>UM	gbM<UM	*A. thaliana*	gbM<UM	gbM<UM	gbM<UM
B. rapa	gbM>UM	gbM>UM	gbM~UM	*A. thaliana*	gbM~UM	gbM<UM	gbM~UM
B. oleracea	gbM>UM	gbM>UM	gbM<UM	*A. thaliana*	gbM<UM	gbM<UM	gbM<UM
E. salsugineum	gbM<UM	gbM<UM	gbM<UM	*A. thaliana*	-------------	-------------	-------------
E. grandis	gbM>UM	gbM>UM	gbM<UM	*A. thaliana*	gbM~UM	gbM<UM	gbM>UM
C. clementina	gbM>UM	gbM>UM	gbM<UM	*A. thaliana*	gbM<UM	gbM<UM	gbM<UM
V. vinifera	gbM>UM	gbM>UM	gbM<UM	*A. thaliana*	gbM<UM	gbM<UM	gbM~UM
S. lycopersicum	gbM>UM	gbM>UM	gbM<UM	*A. thaliana*	gbM~UM	gbM<UM	gbM<UM
M. guttatus	gbM>UM	gbM>UM	gbM>UM	*A. thaliana*	gbM<UM	gbM<UM	gbM>UM
B. vulgaris	gbM>UM	gbM>UM	gbM<UM	*A. thaliana*	gbM<UM	gbM~UM	gbM~UM
O. sativa	gbM>UM	gbM>UM	gbM<UM	--------------	-------------	-------------	-------------
B. distachyon	gbM>UM	gbM>UM	gbM<UM	*O. sativa*	gbM>UM	gbM<UM	gbM>UM
S. viridis	gbM>UM	gbM>UM	gbM<UM	*O. sativa*	gbM~UM	gbM<UM	gbM>UM
P. virgatum	gbM>UM	gbM>UM	gbM<UM	*O. sativa*	gbM>UM	gbM<UM	gbM>UM
P. hallii	gbM>UM	gbM>UM	gbM<UM	*O. sativa*	gbM>UM	gbM<UM	gbM>UM
S. bicolor	gbM>UM	gbM>UM	gbM<UM	*O. sativa*	gbM>UM	gbM<UM	gbM>UM
Z. mays	gbM>UM	gbM>UM	gbM<UM	*O. sativa*	gbM>UM	gbM<UM	gbM~UM
A. trichopoda	gbM>UM	gbM>UM	gbM>UM	*A. thaliana*	gbM~UM	gbM<UM	gbM~UM

Figure S20: Gene expression levels (FPKM) for all genes, CG gbM genes, mCHG genes, mCHH, and TSS-mCG genes for second replicate of C. sativus.

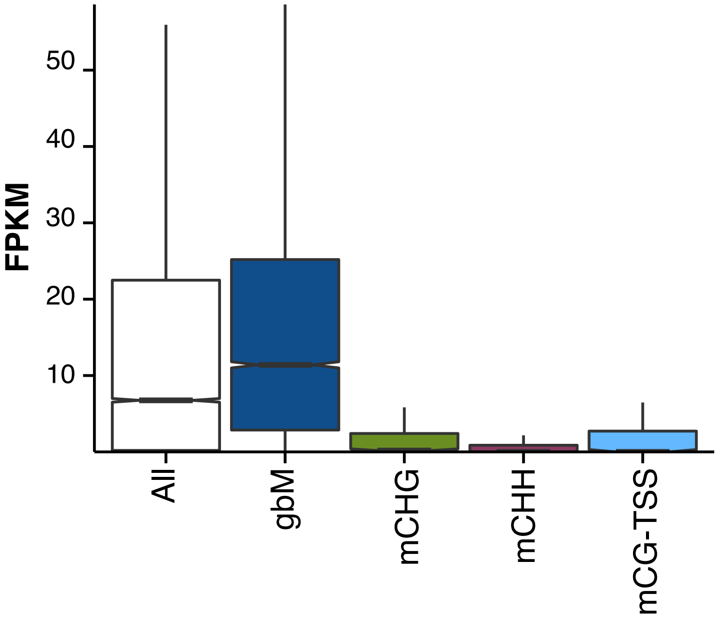

Figure S21: Methylation states (CG gbM = blue, mCHG = green, mCHH = maroon, UM = white) of genes (horizontal axis) orthologous to mCHH genes in **(A)** *L. japonicus*, **(B)** *A. lyrata,* and **(C)** *Z. mays.*

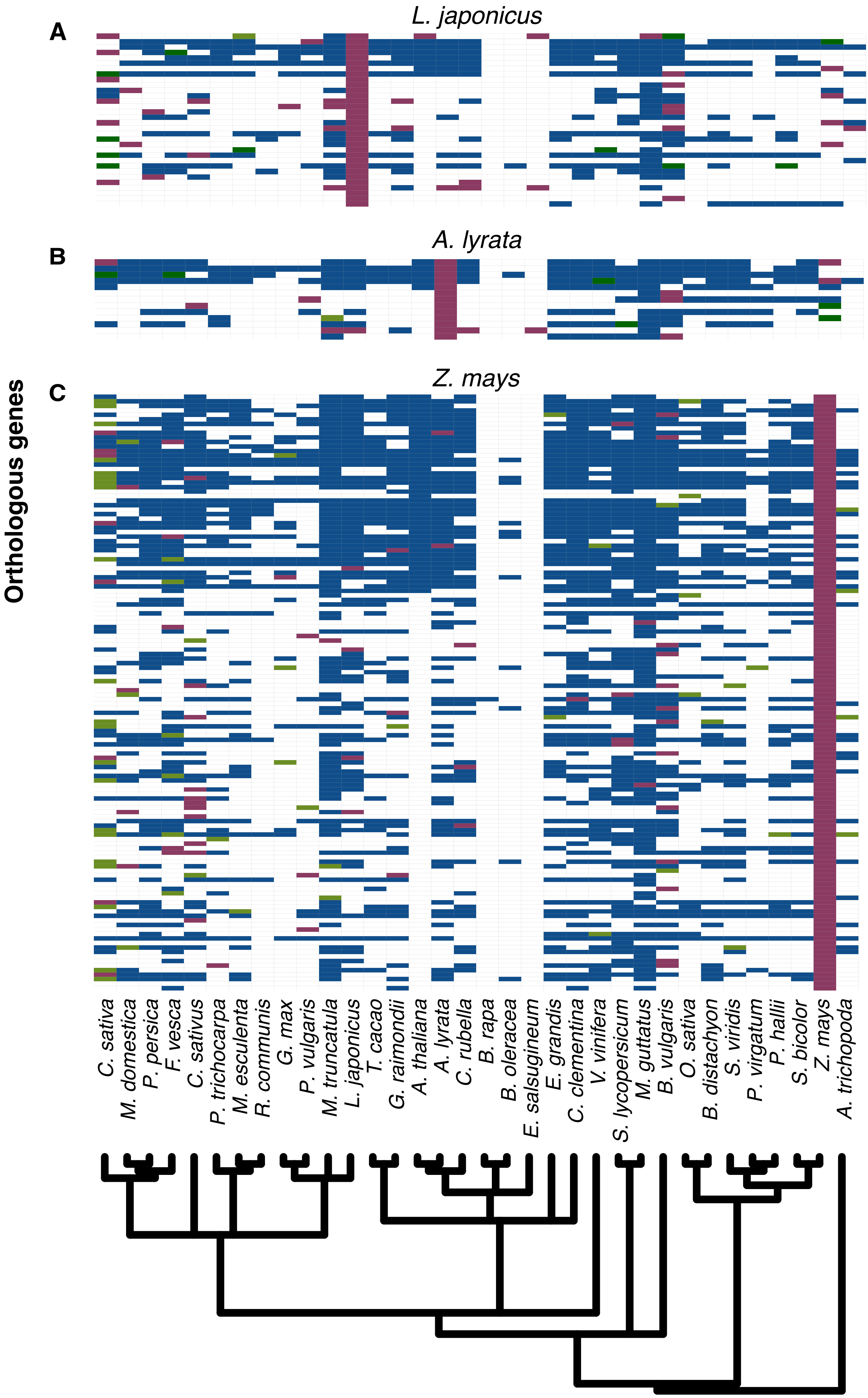

Figure S22: GO term enrichment was performed for each gene class for each species. The most commonly enriched GO terms (horizontal axis) for the Biological Process (first panel), Molecular Function (second panel) and Cellular Component (third panel) are shown for **(A)** CG gbM genes, **(B)** mCHG genes, and **(C)** mCHH genes.

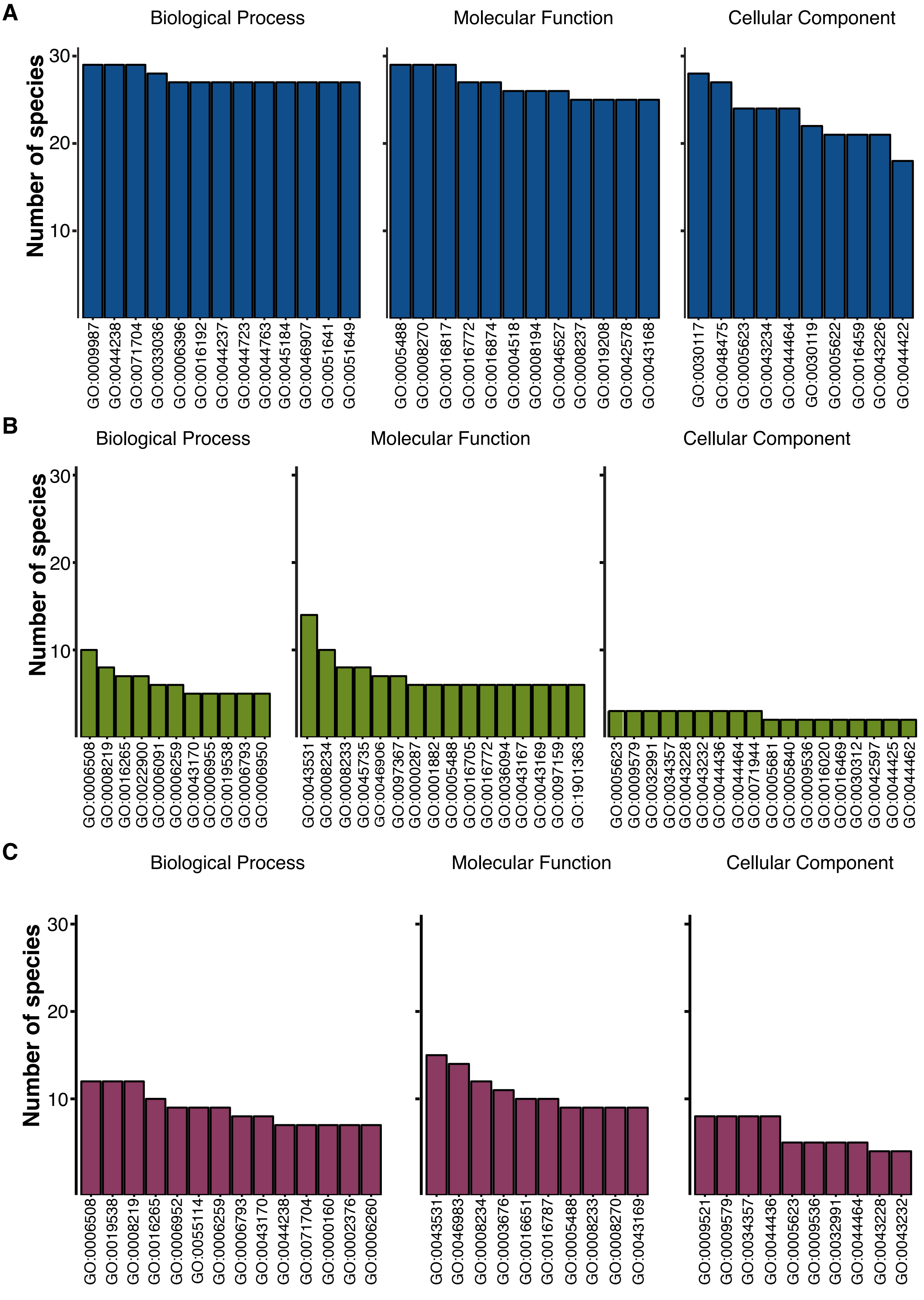

Figure S23: Association of transposable elements (TEs) 2000 bps upstream, within gene bodies, and 2000 bps downstream with gbM genes (blue), mCHG genes (green), mCHH genes (maroon), and all genes (grey). Enrichment of each TEs for each set of genes is indicated by asterisk (* p-value < 0.05, ** p-value < 0.01, *** p-value < 0.001).

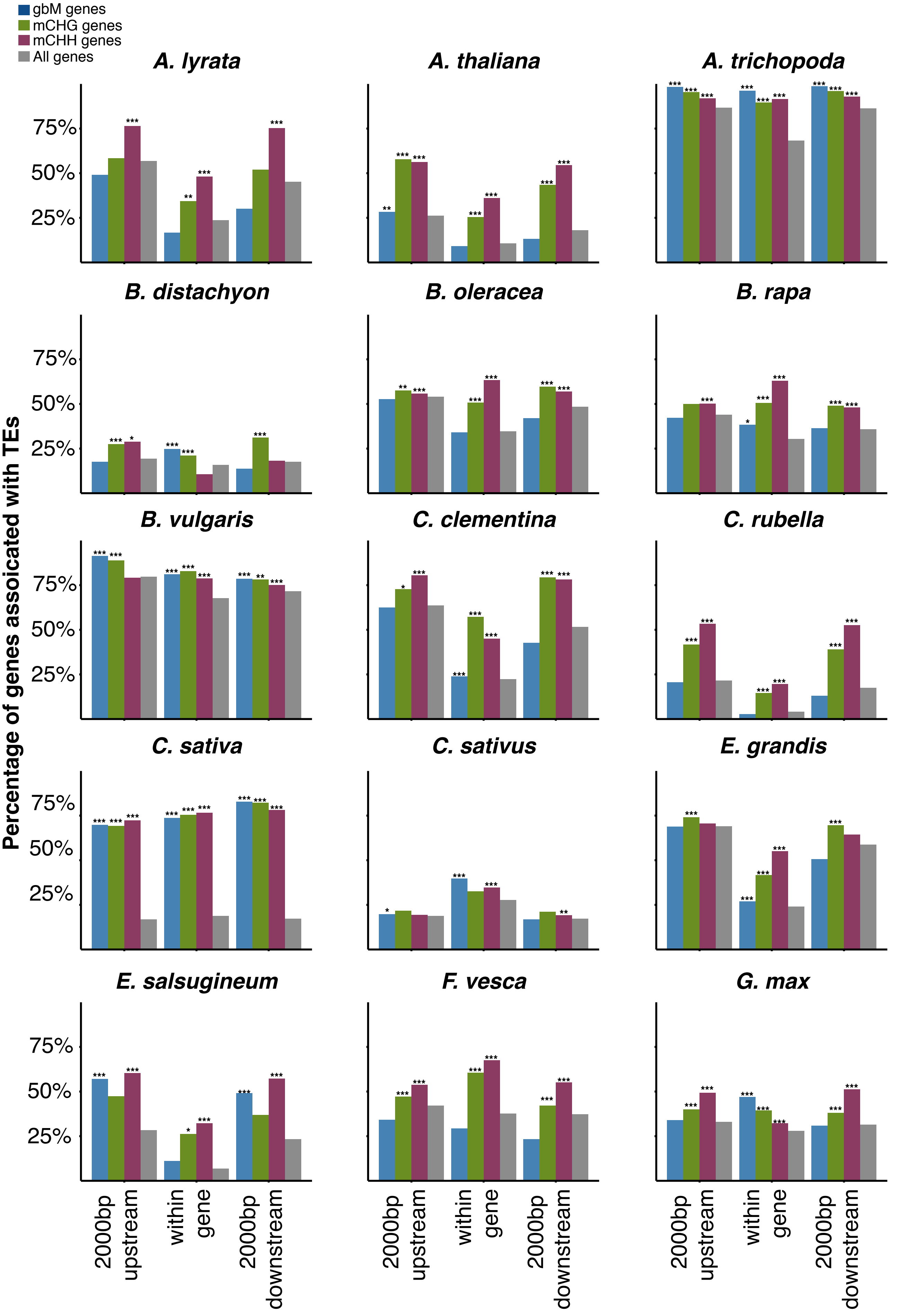

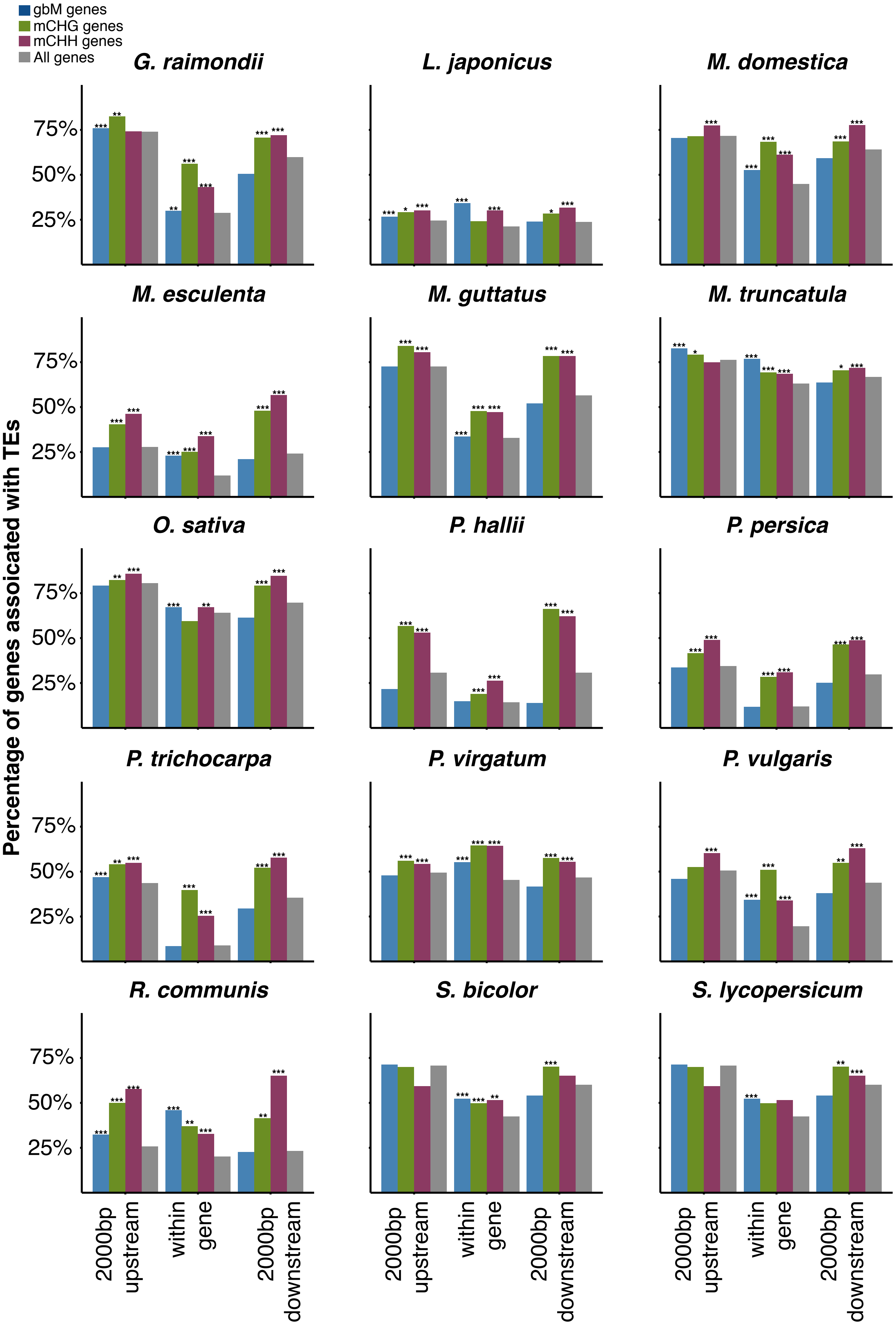

gbM genes
mCHG genes
mCHH genes
All genes
Percentage of genes assoicated with TEs
G. raimondii
L. japonicus
M. domestica
M. esculenta
M. guttatus
M. truncatula
O. sativa
P. hallii
P. persica
P. trichocarpa
P. virgatum
P. vulgaris
R. communis
S. bicolor
S. lycopersicum
75%
50%
25%
2000bp upstream
within gene
2000bp downstream

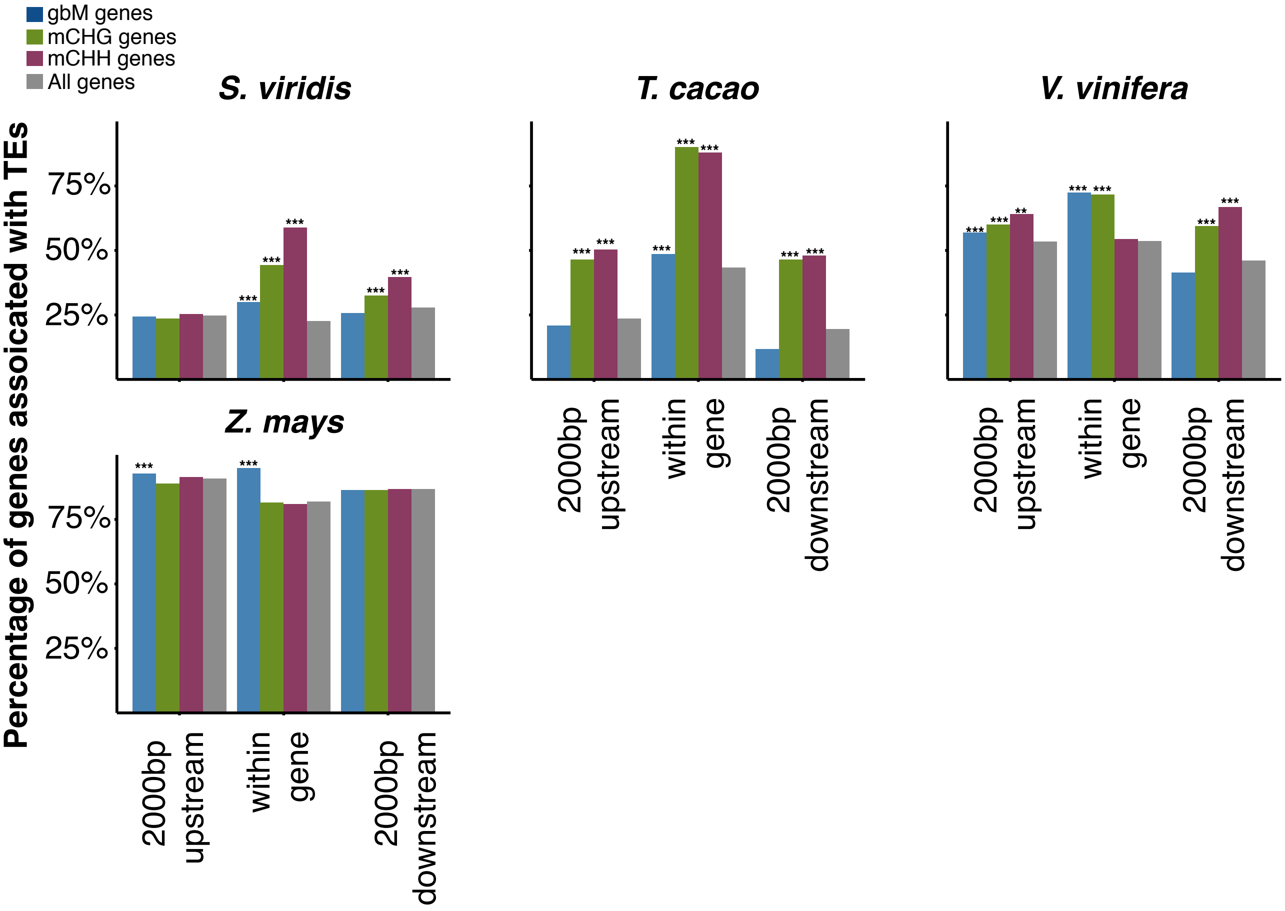

gbM genes
mCHG genes
mCHH genes
All genes
S. viridis
T. cacao
V. vinifera
Z. mays
Percentage of genes assoicated with TEs
75%
50%
25%
***
**
2000bp upstream
within gene
2000bp downstream

Figure S24: Methylation levels for mCG (blue), mCHG (green), and mCHH (maroon), upstream, across, and downstream for all conserved non-coding sequences (CNS), CNS proximally located to genes (1 < 1kb), CNS distally located to genes (> 1kb), and CNS located within utr and intronic sequences.

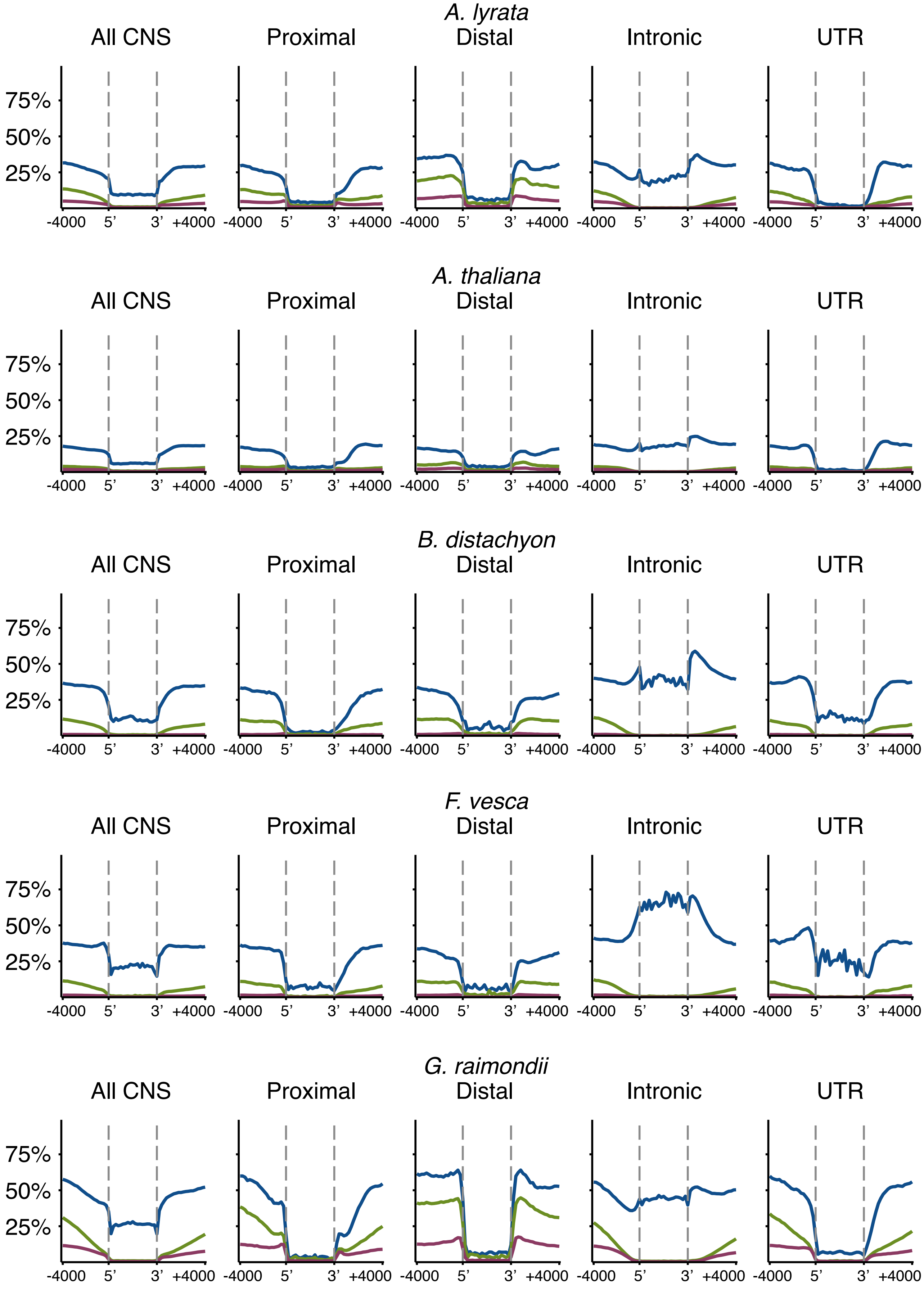

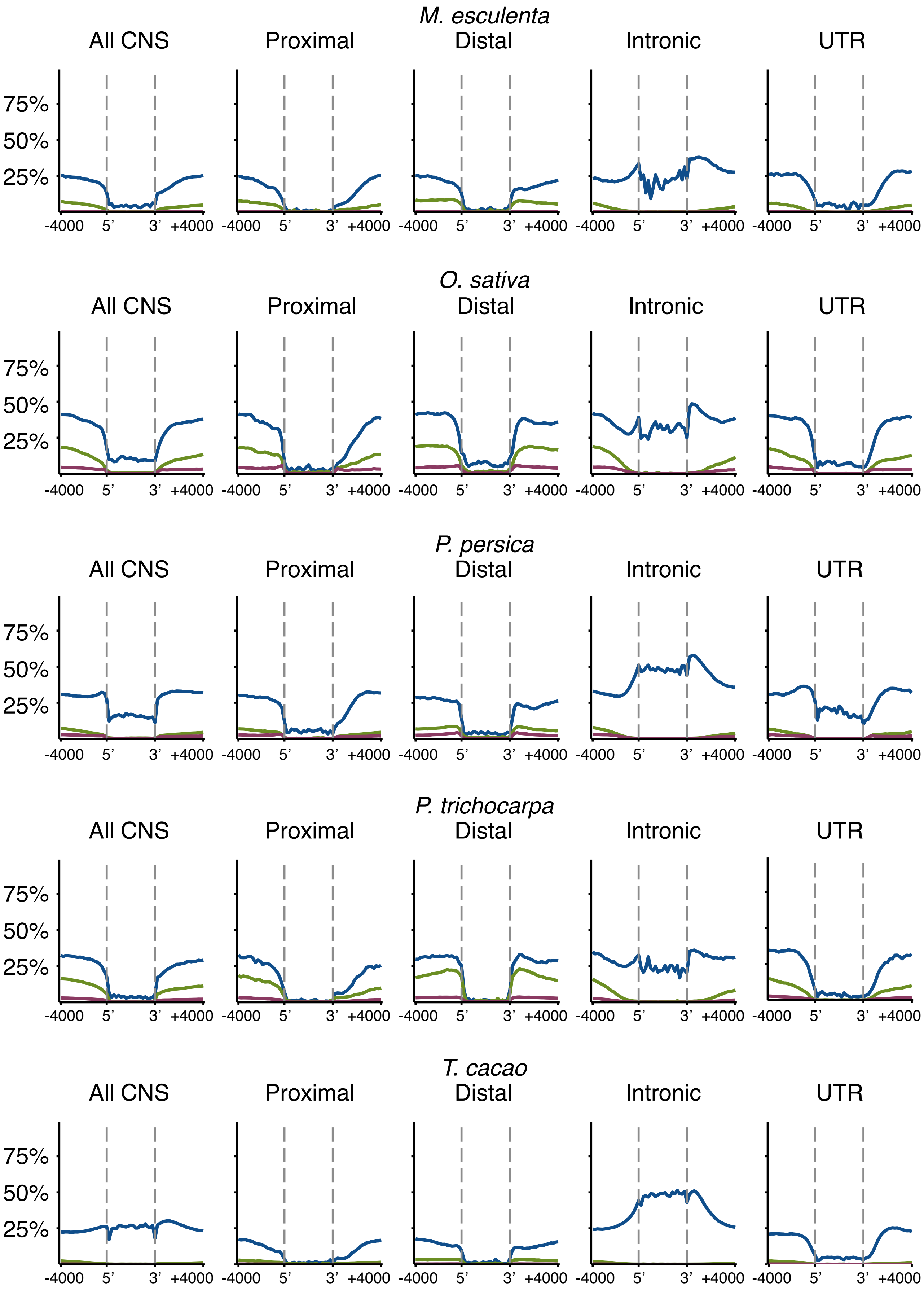
M. esculenta
O. sativa
P. persica
P. trichocarpa
T. cacao
All CNS
Proximal
Distal
Intronic
UTR
75%
50%
25%
-4000
5’
3’
+4000

Figure S20: Association of upstream and downstream mCHH islands and gene expression. Genes are classified as NE = not expressed, 1 = first (lowest) quartile of expression, 2 = second quartile of expression, 3 = third quartile of expression, 4 = fourth (highest) quartile of expression. ** indicates a difference in proportion from the fourth quartile at $p < 0.01$.

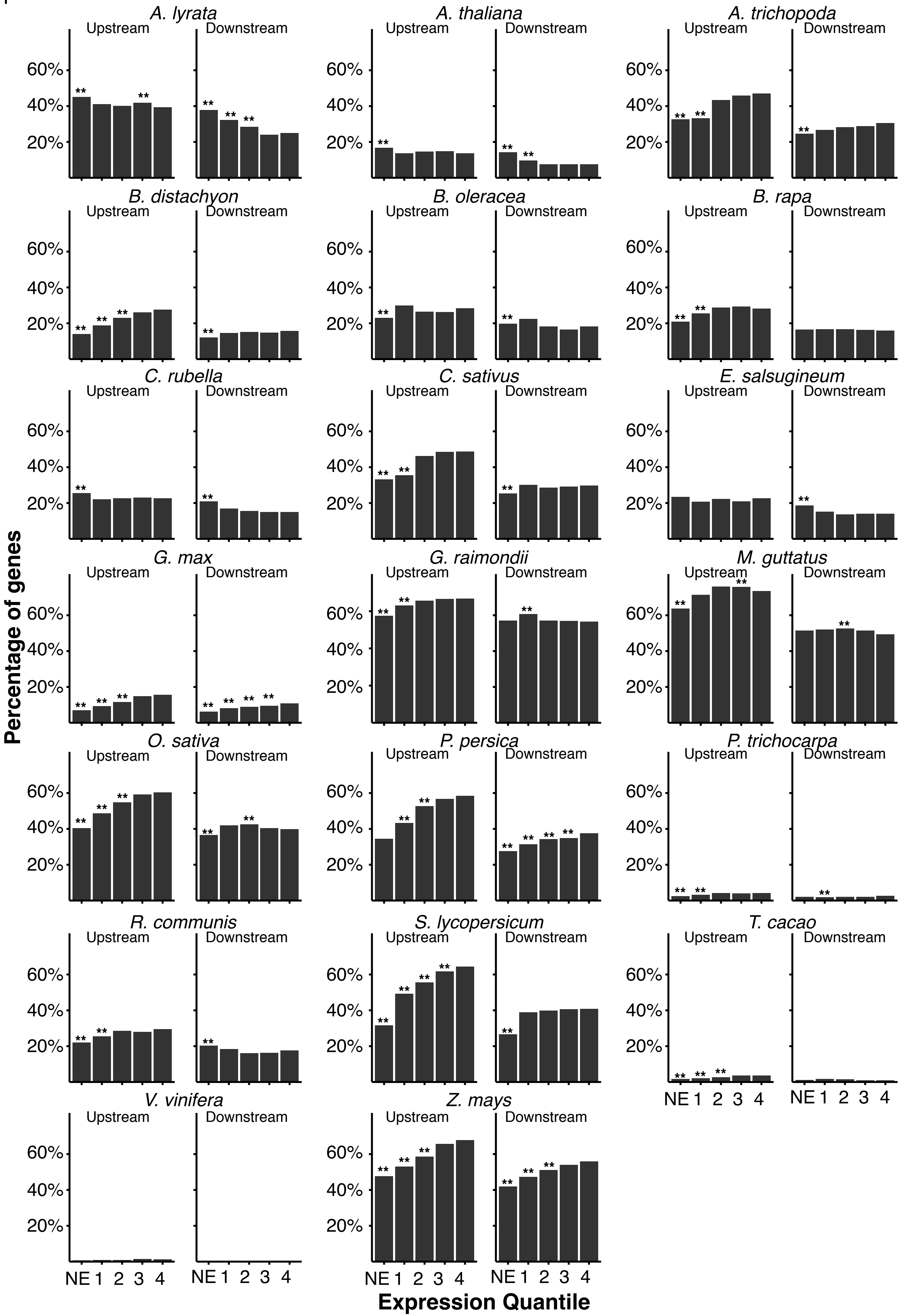

Figure S26: Methylation patterns of mCHH islands for all species. Methylation levels for mCG (blue), mCHG (green), and mCHH (maroon) were plotted for 100bp windows centered on the peak level of mCHH CHH islands found upstream of genes.

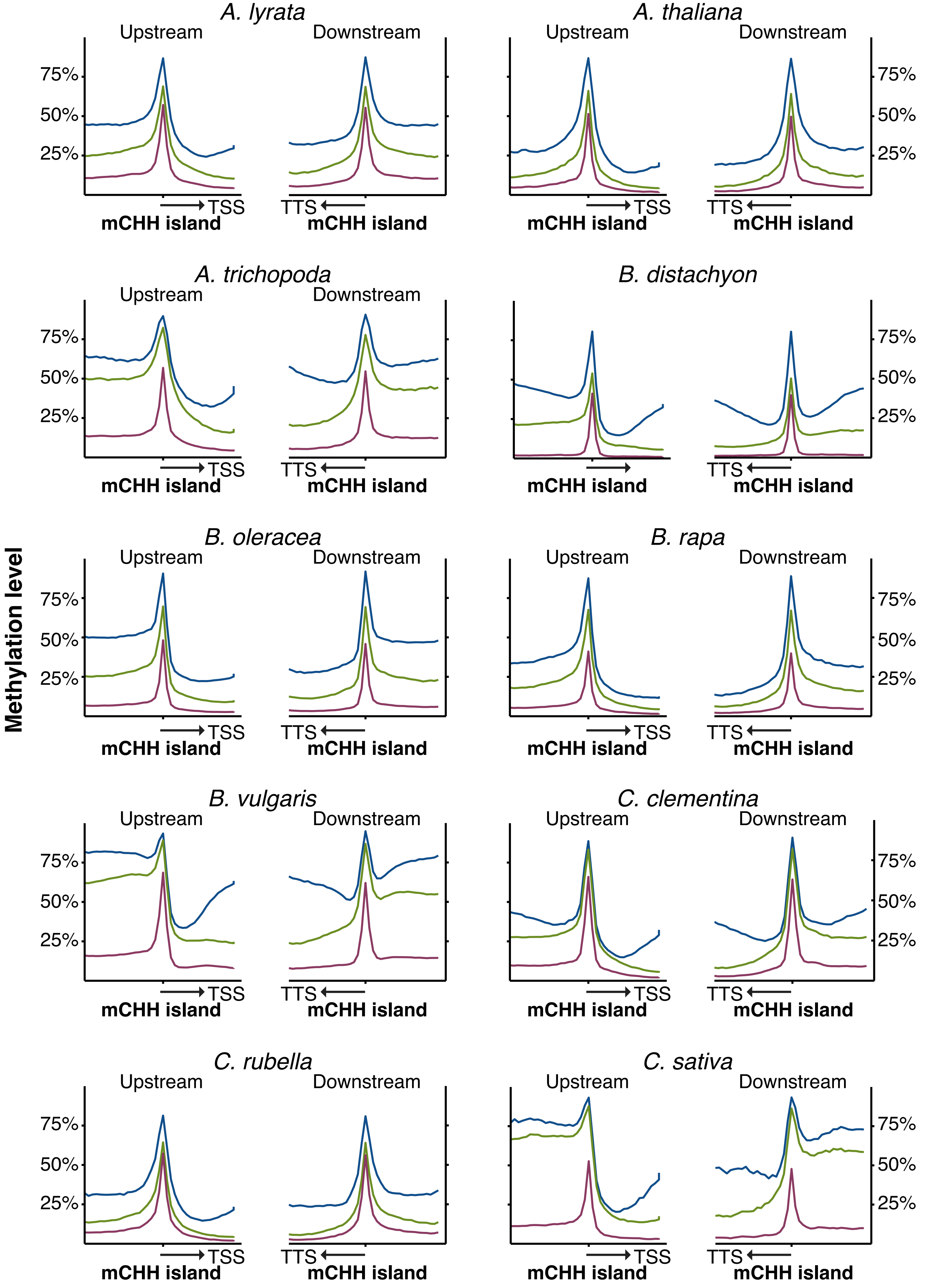

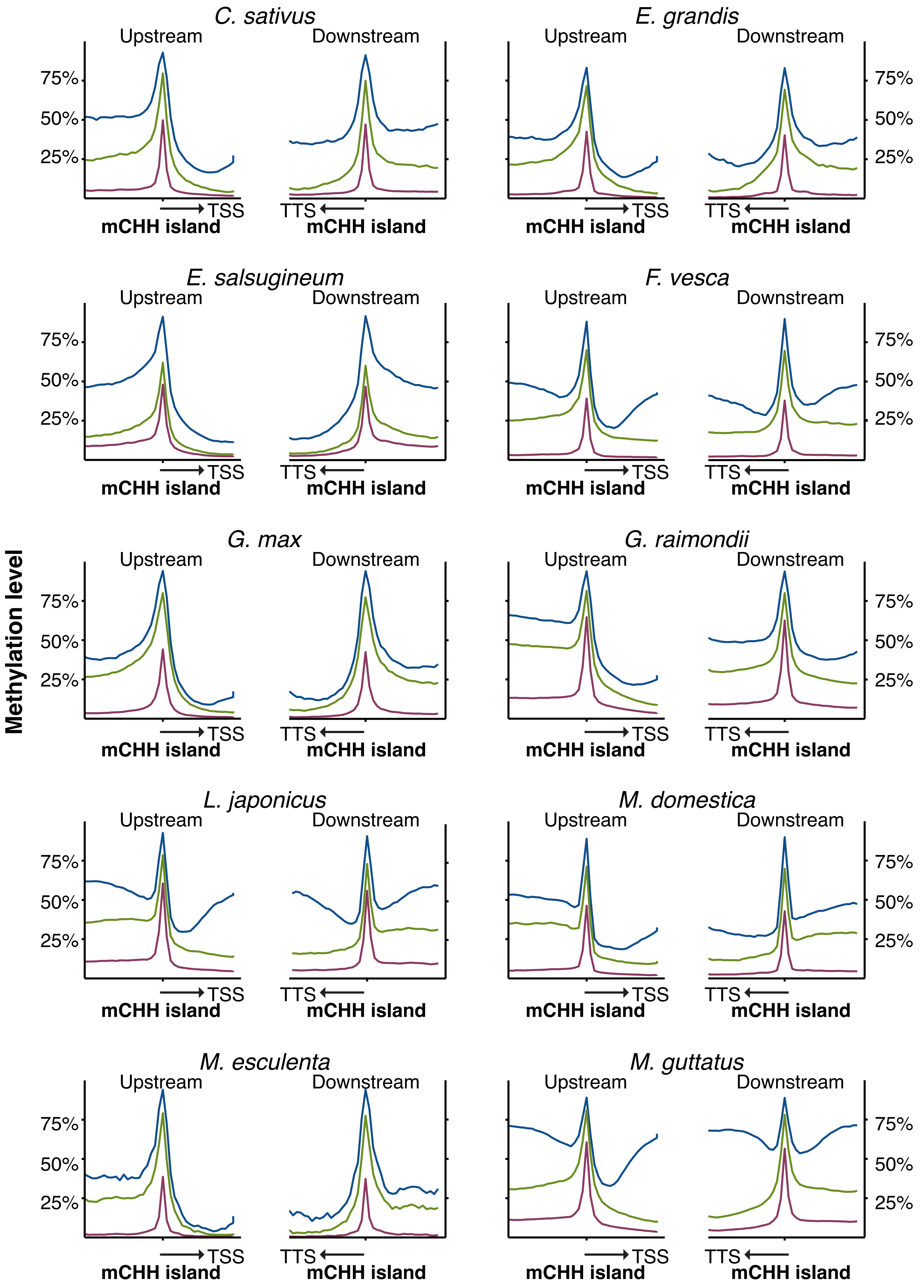
Methylation level
C. sativus
E. grandis
E. salsugineum
F. vesca
G. max
G. raimondii
L. japonicus
M. domestica
M. esculenta
M. guttatus
Upstream
Downstream
75%
50%
25%
TSS
TTS
mCHH island

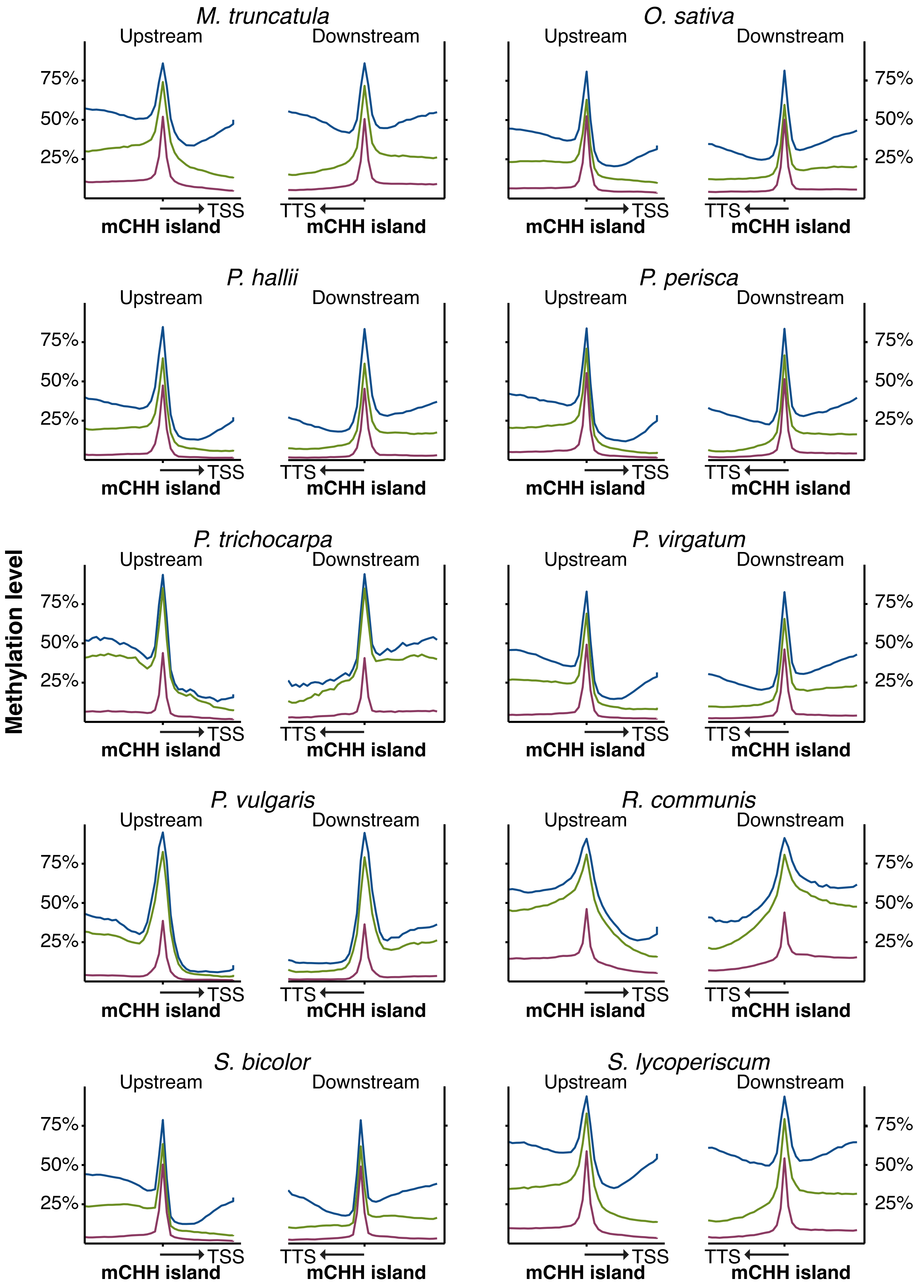
Methylation level
M. truncatula
O. sativa
P. hallii
P. perisca
P. trichocarpa
P. virgatum
P. vulgaris
R. communis
S. bicolor
S. lycoperiscum
Upstream
Downstream
75%
50%
25%
TSS
TTS
mCHH island

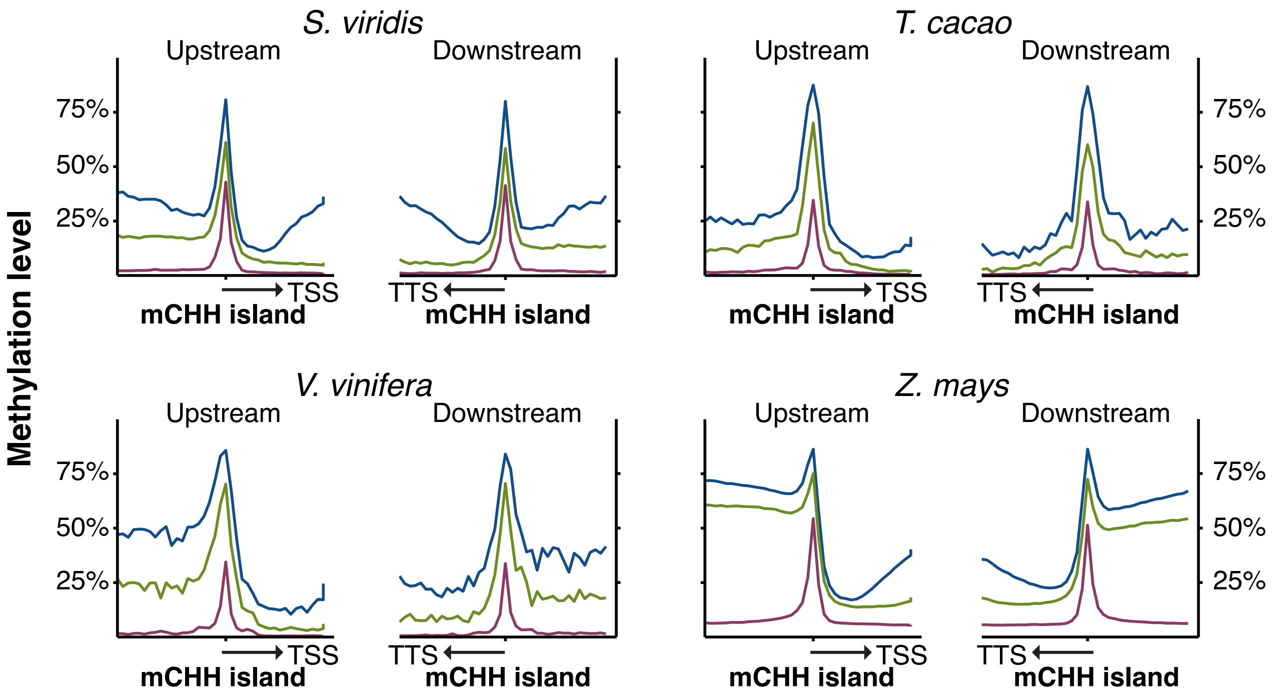
Methylation level
S. viridis
T. cacao
V. vinifera
Z. mays
Upstream
Downstream
75%
50%
25%
TSS
TTS
mCHH island

Figure S27: (A) Percentage of genes with upstream (blue) or downstream (orange) mCHH-islands plotted against total number of repeats. **(B)** Upstream and downstream mCHH islands are correlated with percentage upstream (blue) and percentage of downstream repeats (orange). **(C)** Percentage of genes with upstream (blue) or downstream (orange) repeats plotted against total number of repeats.

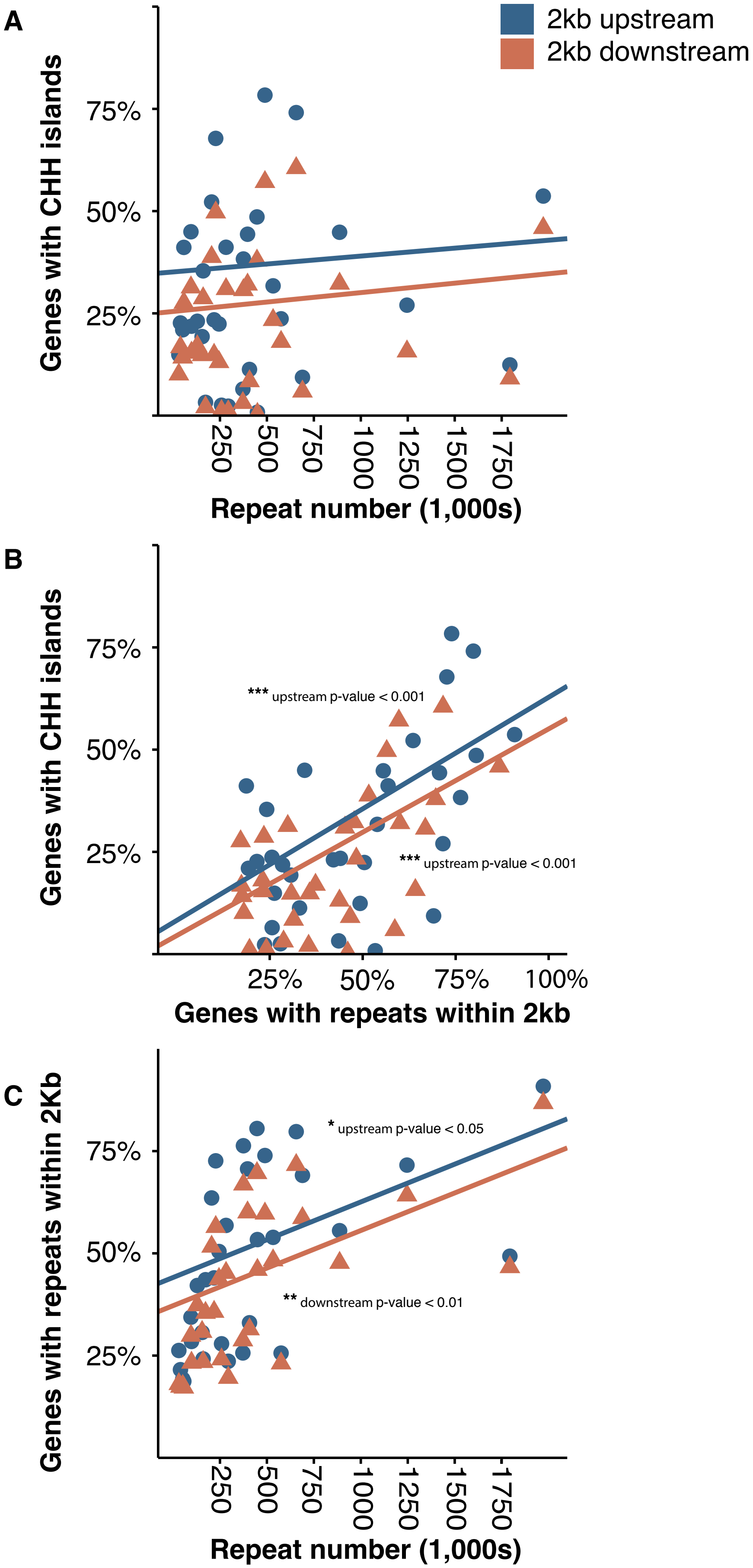

Supplement: Additional file 3: — Supplemental Table and Figures. Table S5 and Figures S11-27 [file 13059_2016_1059_MOESM3_ESM.pdf]
